# Proton-coupled electron transfer reactivities of electronically divergent heme superoxide intermediates: a kinetic, thermodynamic, and theoretical study†

**DOI:** 10.1039/d1sc01952j

**Published:** 2021-05-27

**Authors:** Pritam Mondal, Izumi Ishigami, Emilie F. Gérard, Chaeeun Lim, Syun-Ru Yeh, Sam P. de Visser, Gayan B. Wijeratne

**Affiliations:** Department of Chemistry, University of Alabama at Birmingham Birmingham AL 35205 USA wijeratne@uab.edu; Department of Physiology and Biophysics, Albert Einstein College of Medicine The Bronx New York 10461 USA syun-ru.yeh@einsteinmed.org; Manchester Institute of Biotechnology, Department of Chemical Engineering and Analytical Science, The University of Manchester 131 Princess Street Manchester M1 7DN UK sam.devisser@manchester.ac.uk

## Abstract

Heme superoxides are one of the most versatile metallo-intermediates in biology, and they mediate a vast variety of oxidation and oxygenation reactions involving O_2(g)_. Overall proton-coupled electron transfer (PCET) processes they facilitate may proceed *via* several different mechanistic pathways, attributes of which are not yet fully understood. Herein we present a detailed investigation into concerted PCET events of a series of geometrically similar, but electronically disparate synthetic heme superoxide mimics, where unprecedented, PCET feasibility-determining electronic effects of the heme center have been identified. These electronic factors firmly modulate both thermodynamic and kinetic parameters that are central to PCET, as supported by our experimental and theoretical observations. Consistently, the most electron-deficient superoxide adduct shows the strongest driving force for PCET, whereas the most electron-rich system remains unreactive. The pivotal role of these findings in understanding significant heme systems in biology, as well as in alternative energy applications is also discussed.

## Abstract 2

Electronic characteristics of heme significantly influence the feasibility of hydrogen atom abstraction by synthetic heme superoxide moieties, shedding new light on analogous scenarios implicated in both biological and alternate energy applications.

## Graphical abstract

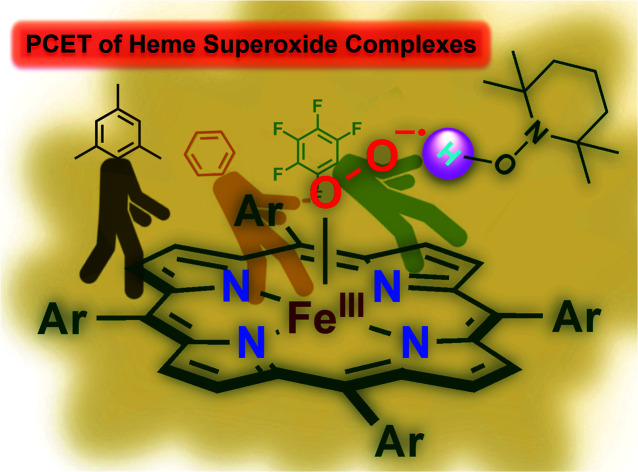

## Introduction

**Chart 1:**
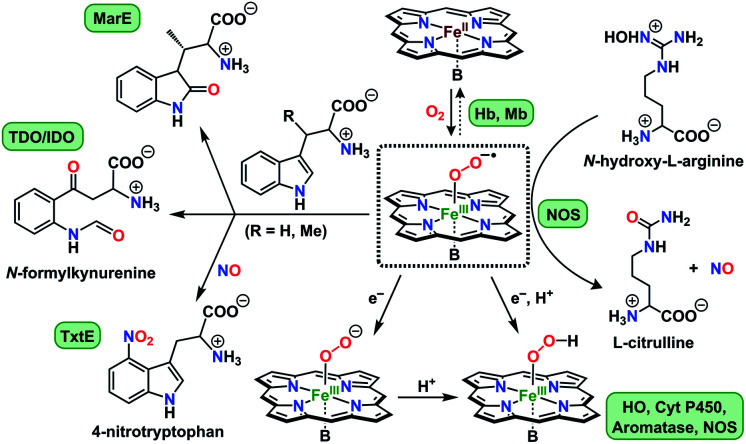
Versatile biological reactivity pathways of heme superoxo/heme oxy intermediates (shown in the center); B = axially coordinating amino acid side chain at the heme proximal site.

Activation of dioxygen by heme proteins plays a pivotal role in metalloenzyme-mediated oxidation, oxygenation, and dioxygen reduction reactions in biology.^1^ The central paradigm of this dioxygen binding and activation process by heme centers embodies a distinctive panel of intermediates, where the stepwise reduction of O_2_ leading up to O–O bond cleavage occurs in parallel to oxidation of the heme iron center. The initial heme–dioxygen adduct (*i.e.*, heme–superoxo (Fe^III^–O_2_^−^˙) or heme–oxy (Fe^II^–O_2_) species) is common to all dioxygen activating heme enzymes, and exhibits a remarkably divergent reactivity profile mainly depending upon the intricate structural tuning within a given active site. Specifically, the identity and properties of the amino acid side chain ligating at the heme proximal site, distal and proximal non-covalent interactions about the heme center, and electronic properties of the heme ligand itself,^1*a*,2^ all of which, in concert choreograph the specific biological role of a heme superoxide intermediate. These include implications in (Chart 1): (i) reversible dioxygen binding in hemoglobin (Hb) and myoglobin (Mb);^3^ (ii) indole dioxygenation reactivity in tryptophan and indoleamine 2,3-dioxygenases (TDO/IDO);^4^ (iii) indole monooxygenation by MarE;^5^ (iv) interaction with physiologically present nitric oxide (˙NO_(g)_) to generate heme peroxynitrite (Fe^III^–OONO) species in tryptophan nitrating TxtE^6^ and other proteins;^7^ (v) reactivity with electrons and/or protons in native mechanisms of heme oxygenase (HO),^1*b*,8^ cytochrome P450 (Cyt P450),^9^ aromatase,^10^ and nitric oxide synthase (NOS);^11^ (vi) abstraction of a hydrogen atom in one of the proposed mechanisms of nitric oxide synthase.^1*a*,12^ Particularly, heme superoxide reactivities with exogenous electrons and/or electrons and protons are central to an array of heme enzymes, where the site of reduction and/or protonation is critical for the overall outcome of the biological function. For example, reduction followed by protonation of the distal oxygen atom with respect to the iron center gives rise to the corresponding heme hydroperoxide species (*e.g.*, Cyt. P450),^9*d*^ whereas the protonation at the proximal oxygen has been often shown to liberate protonated superoxide, as in the case of unproductive degradation of oxyhemoglobin to met-hemoglobin.^13^

Detailed physicochemical elucidation of reduction–protonation chemistries of heme superoxide adducts is also of supreme interest with regard to the oxygen reduction reaction (ORR), a cornerstone in numerous critical biological (*e.g.*, cellular respiration and oxidative phosphorylation) and industrial (*e.g.*, synthetic catalysis and batteries) processes.^14^ In that, whether the heme superoxide abstracts a hydrogen atom (H˙ = H^+^ + e^−^) in a single mechanistic step (*i.e.*, concerted), or an electron and a proton (or *vice versa*) in two consecutive steps (*i.e.*, stepwise) is salient in dictating the overall thermodynamics of the reaction landscape. All of these concerted and stepwise mechanistic possibilities fall under the umbrella of proton-coupled electron transfer (PCET) reactions,^15^ of which, the precise mechanistic details are solely dependent on a few key thermodynamic parameters (*vide infra*).^16^ Recent thorough investigations by Mayer and coworkers have underscored the importance of PCET processes of heme superoxide adducts with relevance to the cathodic reaction in fuel cells, where efficient, cheap dioxygen reducing metallocatalysts could be of momentous importance.^17^ Specifically, the proton affinity of the heme-bound superoxide moiety is critical as it dictates the overpotential barrier of the ORR catalyst, thereby determining the rate/efficiency of the catalytic process.^17*b*,18^ Essentially, the p*K*_a_ of the protonating acid is climacteric for the overall catalytic outcome as it should deliver weakly interacting protons that enhance the susceptibility of heme superoxide toward reduction, while preventing dissociation of protonated superoxide; protonated superoxide radicals are highly unstable, and have long known to decay giving O_2_ and H_2_O_2_ (2HO_2_^−^˙ → H_2_O_2_ + O_2_).^19^ Such subtleties related to p*K*_a_ of the proton source are also critical further downstream in the ORR pathway, where protonation of proximal or distal O-atom of the heme hydroperoxide dictates whether 2-electron (O_2(g)_ + 2H^+^ + 2e^−^ → H_2_O_2_) or 4-electron (O_2(g)_ + 4H^+^ + 4e^−^ → 2H_2_O) reduction of oxygen is accomplished, respectively.^20^ Patently, the latter process is of preference for fuel cell applications, where the complete 4-electron reduction of O_2(g)_ to H_2_O engender an efficient cathodic reaction, preventing the generation of partially reduced reactive oxygen species. Suitably, the detailed body of work by Dey and coworkers on bio-inspired ORR electrocatalysis have offered classic examples of how to adapt inexpensive, environmentally benign metallosystems for the efficient reduction of O_2(g)_ to H_2_O.^20*c*,21^

Despite the widespread significance of heme superoxide mediated PCET pathways, a comprehensive fundamental understanding of the exact mechanistic, structural, and thermochemical parameters governing these processes is still lacking. Synthetic small molecule models can be useful tools in this endeavor, where in-depth thermodynamic and kinetic investigations into systematically varied heme structures are more feasible. Nonetheless, heme superoxide mimics are abundantly known as incompetent oxidants, and their directed reactivities with organic substrates are extremely scarce. Indeed, our recent work marks the first report where synthetic heme superoxide intermediates were shown to react with exogenously added indole substrates in the efficient modelling of tryptophan dioxygenation chemistry of indoleamine and tryptophan 2,3-dioxygenases.^22^ Similarly, heme superoxide adducts that efficiently react with added acids (*i.e.*, protons (H^+^)), reductants (*i.e.*, electrons(e^−^)), and/or H˙ donors are only a handful.^23^ Intriguingly, Naruta,^24^ Dey,^25^ and their coworkers have presented unique examples of heme superoxide intermediates that react with intramolecular H^+^ or H˙ donors, ultimately giving rise to the corresponding heme hydroperoxo (Fe^III^–OOH) adduct. To the best of our knowledge, recent work by Karlin and coworkers is the only instance where proton-coupled electron transfer reactivities of heme superoxide intermediates have been shown, where H˙ abstraction from an exogenous, weak O–H bond substrate generates the corresponding heme hydroperoxo species in a single kinetic step.^26^

**Chart 2:**
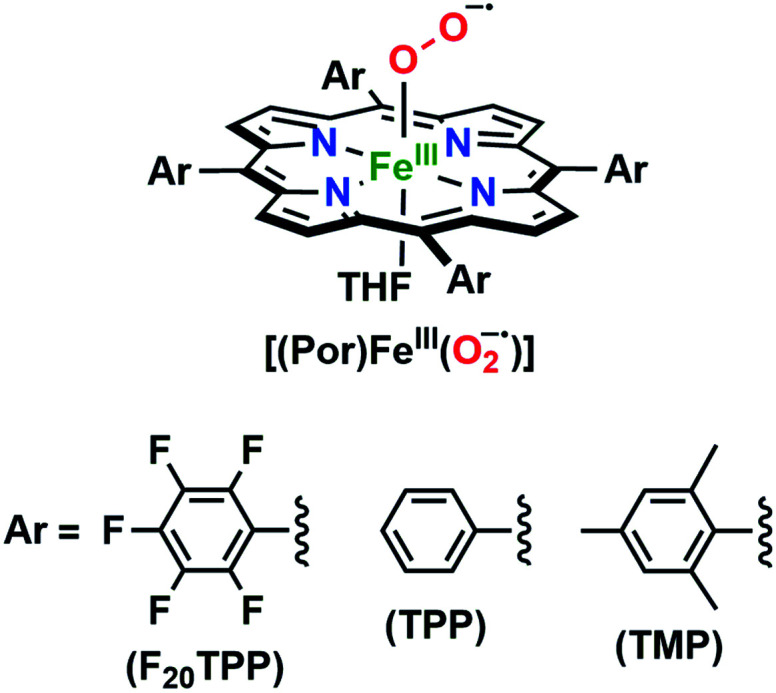
The series of *meso*-substituted heme superoxide intermediates with varied electronic properties discussed in this work.

In the present investigation, we have interrogated the concerted proton–electron (*i.e.*, H˙) abstraction reactivities of three electronically different, geometrically similar heme iron superoxo complexes, **[(Por)FeIII(O2−˙)]** (where Por = porphyrin supporting ligand; Chart 2), using the weak O–H bond substrate TEMPO–H (TEMPO–H = 1-hydroxy-2,2,6,6-tetramethylpiperidine; BDFE = 66.5 kcal mol^−1^ in THF^16*a*^), resulting in the corresponding heme hydroperoxo adduct, **[(Por)FeIII(OOH)]**, under cryogenic conditions. In detail experimental and theoretical analyses into key thermodynamic and kinetic parameters are also presented, revealing intriguing insights into how subtle alterations in electronic atmospheres about heme centers can effect profound outcomes in their physical properties and related reactivities. In that, the most electron-deficient superoxide adduct reacts the fastest with TEMPO–H, while the most electron-rich superoxo species remains unreactive. This reactivity pattern is further corroborated by the experimentally determined (*i.e.*, by means of the Bordwell relationship; eqn (1)^27^) O–H bond dissociation free energies (BDFEs) of the heme hydroperoxo products. This study marks the first report where detailed thermodynamic (elucidation of p*K*_a_, *E*°, and BDFE values) and kinetic (rate comparisons, kinetic isotope effects and activation parameters) investigations are described for a series of structurally similar, electronically divergent heme superoxide intermediates, along with strong theoretical justification. These findings are crucial in the unequivocal comprehension of both biological systems that are indispensable in human therapeutic applications, as well as processes central to alternative energy sources such as fuel cells.

1 BDFE = 1.37(p*K*_a_) + 23.06*E*° + *C*_G,solv_

## Results and discussion

### Formation and PCET reactivities of **[(Por)FeIII(O2−˙)]** complexes

The series of superoxo adducts utilized for this study offer disparate electronic atmospheres about the heme center (Chart 2): F_20_TPP (F_20_TPP = 5,10,15,20-*tetrakis*(pentafluorophenyl)-porphyrin), TPP (TPP = 5,10,15,20-tetraphenylporphyrin) and TMP (TMP = 5,10,15,20-tetramesitylporphyrin). Upon bubbling of dry O_2(g)_ into a THF solution of **[(THF)2(F20TPP)FeII]** (419 nm (Soret; *ε* = 2.5 × 10^5^ M^−1^ cm^−1^) and 539 nm (*ε* = 1.2 × 10^4^ M^−1^ cm^−1^)) at −80 °C, new electronic absorption spectral features centered at 413 nm (Soret; *ε* = 1.58 × 10^5^ M^−1^ cm^−1^) and 532 nm (*ε* = 1.3 × 10^4^ M^−1^ cm^−1^) were generated, indicating the formation of the EPR-silent ferric superoxo species, **[(F20TPP)FeIII(O2−˙)]** (Fig. S1†). In consistence, the isotopically shifted resonance Raman frequencies for *ν*(Fe–O) and *ν*(O–O) features were observed at 577 (Δ^18^O_2_ = −23) and 1137 (Δ^18^O_2_ = −22) cm^−1^, respectively (Fig. S2†). The rest of the series of heme superoxo adducts (*i.e.*, **[(TPP)FeIII(O2−˙)]** and **[(TMP)FeIII(O2−˙)]**) were prepared similarly (Fig. S1†), and their spectroscopic signatures are in strong agreement with previous reports.^1*b*,22,28^

**Fig. 1:**
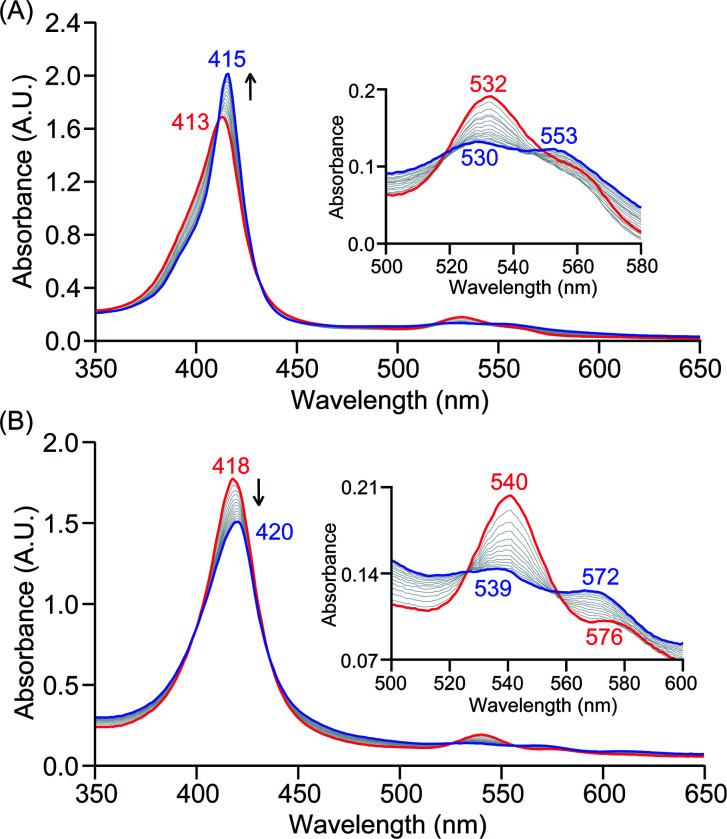
Electronic absorption spectral changes observed (in THF at −80 °C) during the reaction of a 50 μM solution of (A) **[(F20TPP)FeIII(O2−˙)]** and (B) **[(TPP)FeIII(O2−˙)]** with 100 equiv. of TEMPO–H (red = initial ferric superoxo complex; blue = final ferric product). Insets show the expanded Q-band regions, and arrows indicate the direction of peak transition.

**Scheme 1:**
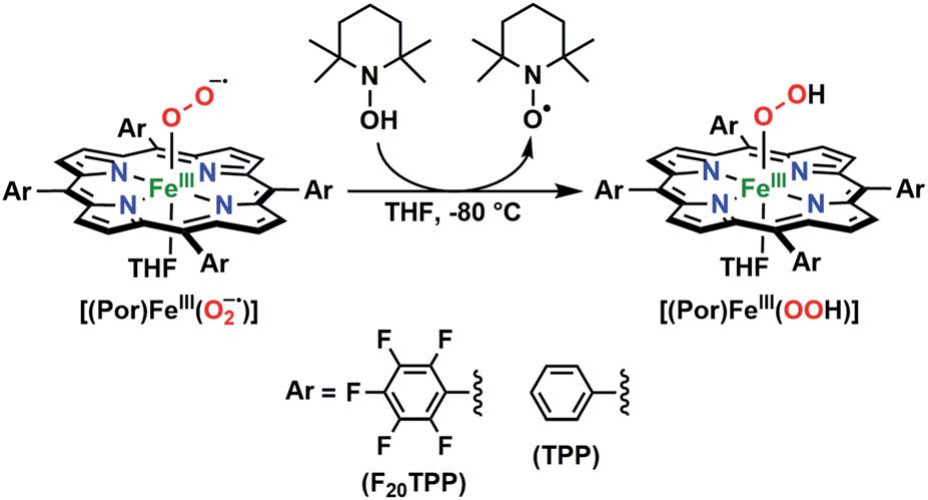
Generalized reaction scheme depicting hydrogen atom abstraction reactivities involving heme superoxide adducts and TEMPO–H substrate.

The PCET reactivities of these heme superoxide oxidants were then evaluated against variable concentrations of the TEMPO–H substrate (BDFE = 66.5 kcal mol^−1^ in THF). When 100 equiv. of TEMPO–H was added into a solution of **[(F20TPP)FeIII(O2−˙)]** in THF at −80 °C, patent changes in absorption features (Soret: 413 to 415 nm; Q-band: 532 to 530 and 553 nm) were evidenced (Fig. 1A), indicating the H˙ abstraction reactivity of **[(F20TPP)FeIII(O2−˙)]** with TEMPO–H (Scheme 1). Importantly, the H˙ here is transferred in a single mechanistic step (*i.e.*, concerted), rather than a H^+^ and an e^−^ in two separate steps. This is due to only H^+^ or e^−^ transfer from TEMPO–H substrate being extremely thermodynamically uphill compared to H˙ transfer.^16*a*,*c*^**[(TPP)FeIII(O2−˙)]** also exhibited a similar PCET reactivity with TEMPO–H (Soret: 418 to 420 nm; Q-band: 540 and 576 to 539 and 572 nm) under identical experimental conditions (Fig. 1B). On the contrary however, no reactivity was observed between **[(TMP)FeIII(O2−˙)]** and TEMPO–H. Moreover, several other PCET substrates with weak C–H (*i.e.*, xanthene (BDFE = 73.3 kcal mol^−1^ in DMSO^29^), 9,10 dihydroanthracene (BDFE = 76 kcal mol^−1^ in DMSO^16*a*^)), and O–H (*i.e.*, 5,6-isopropylidene ascorbic acid (BDFE = 70.5 kcal mol^−1^ in MeCN^30^), *p-*OMe-2,6-di-*tert*-butylphenol (BDFE = 77.4 kcal mol^−1^ in DMSO^29^)) bonds were tested against the above series of heme superoxo adducts, where no evidence of any reactivity could be observed.

### Spectroscopic characterization of **[(Por)FeIII(OOH)]** complexes

**Fig. 2:**
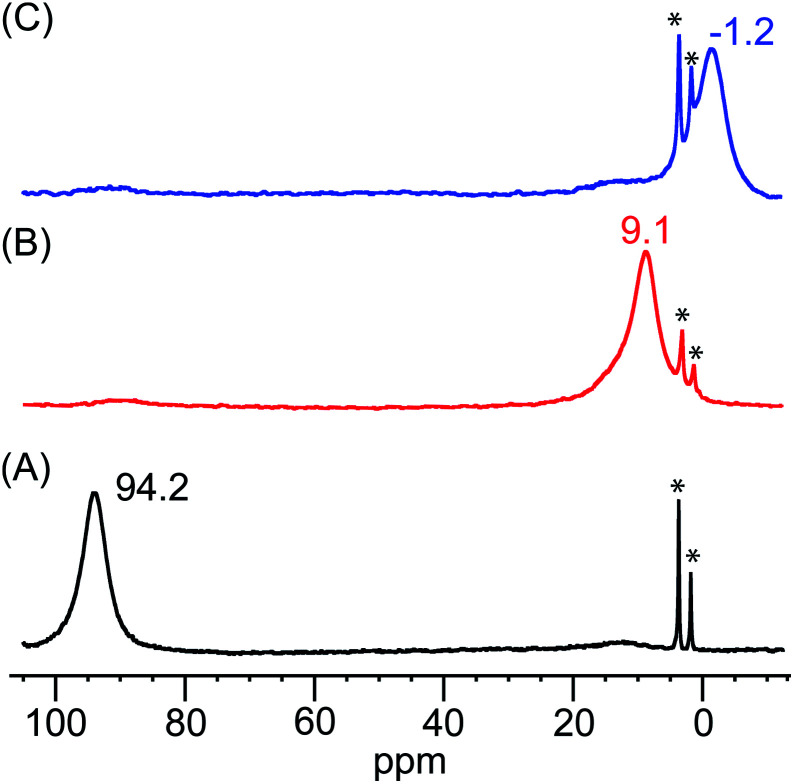
^2^H NMR spectra (in THF at −80 °C) of (A) **[(THF)2(F20TPP-d8)FeII]** (black), (B) **[(F20TPP-d8)FeIII(O2−˙)]** (red), and (C) final heme product of the reaction between **[(F20TPP-d8)FeIII(O2−˙)]** and TEMPO–H (blue) (* peaks at 3.58 and 1.73 ppm correspond to the THF solvent).

**Fig. 3:**
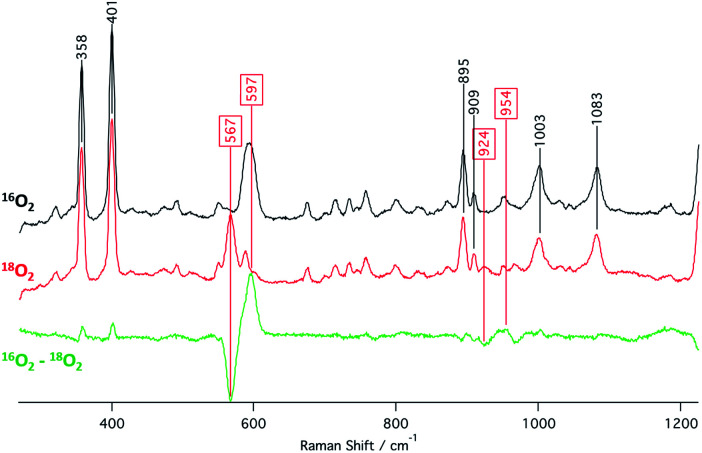
Resonance Raman spectra (*λ*_ex_ = 413.1 nm) collected from a 2 mM frozen THF solution of the final heme product from the reaction between TEMPO–H and **[(F20TPP)FeIII(O2−˙)]** prepared with ^16^O_2(g)_ (black) and ^18^O_2(g)_ (red).

The identities of the expected heme hydroperoxo products (*i.e.*, **[(Por)FeIII(OOH)]**) from PCET reactivities between heme superoxo adducts and TEMPO–H substrate were established as follows: low-temperature ^2^H NMR analyses were carried out on heme complexes supported by the pyrrole-position deuterated **F20TPP-d8** porphyrinate. **[(THF)2(F20TPP-d8)FeII]** and **[(F20TPP-d8)FeIII(O2−˙)]** exhibited a single ^2^H NMR resonance at *δ*_pyrrole_ = 94.2 and *δ*_pyrrole_ = 9.1 ppm, respectively (Fig. 2). The addition of TEMPO–H induced an upfield shift in the pyrrole resonances to *δ*_pyrrole_ = −1.2 ppm, which is indicative of the formation of a low-spin (*S* = 1/2) ferric heme system (Fig. 2).^31^ This is in excellent agreement with the *δ*_pyrrole_^2^H NMR resonances observed for the low-spin **[(F8TPP-d8)FeIII(OOH)]** species by Karlin and coworkers (*δ*_pyrrole_ = −0.63 ppm).^26*a*^ The EPR spectrum of the final reaction mixture of **[(F20TPP)FeIII(O2−˙)]** and TEMPO–H predominantly consists of an organic radical signal (*g* = 2.0; attributed to the TEMPO˙ radical; yield = 82%), which is overlapped with the *S* = 1/2 Fe^III^ features of the low-spin heme hydroperoxo product complex (Fig. S3 and S4†). Notably, all synthetic heme hydroperoxo adducts reported to-date consist of low-spin ferric centers,^23*a*,*c*,24*a*,*b*,25,26,32^ which parallels our aforementioned ^2^H NMR and EPR characterizations. Moreover, the putative **[(F20TPP)FeIII(OOH)]** product complex exhibited the isotopic-sensitive resonance Raman frequency for *ν*(Fe–O) at 597 (Δ^18^O_2_ = −30) cm^−1^ (Fig. 3), which is in-line with other heme hydroperoxo species reported thus far.^23*c*,24*a*,*b*,25,26,33^ The same feature for **[(TPP)FeIII(OOH)]** was observed at 589 (Δ^18^O_2_ = −26) cm^−1^ (Fig. S5†). The identities and yields of the **[(Por)FeIII(OOH)]** products were further substantiated by H_2_O_2_ quantification following acidification, where **[(F20TPP)FeIII(OOH)]** and **[(TPP)FeIII(OOH)]** produced 92% and 88% yields of H_2_O_2_, respectively (Fig. S6†).

**Scheme 2:**
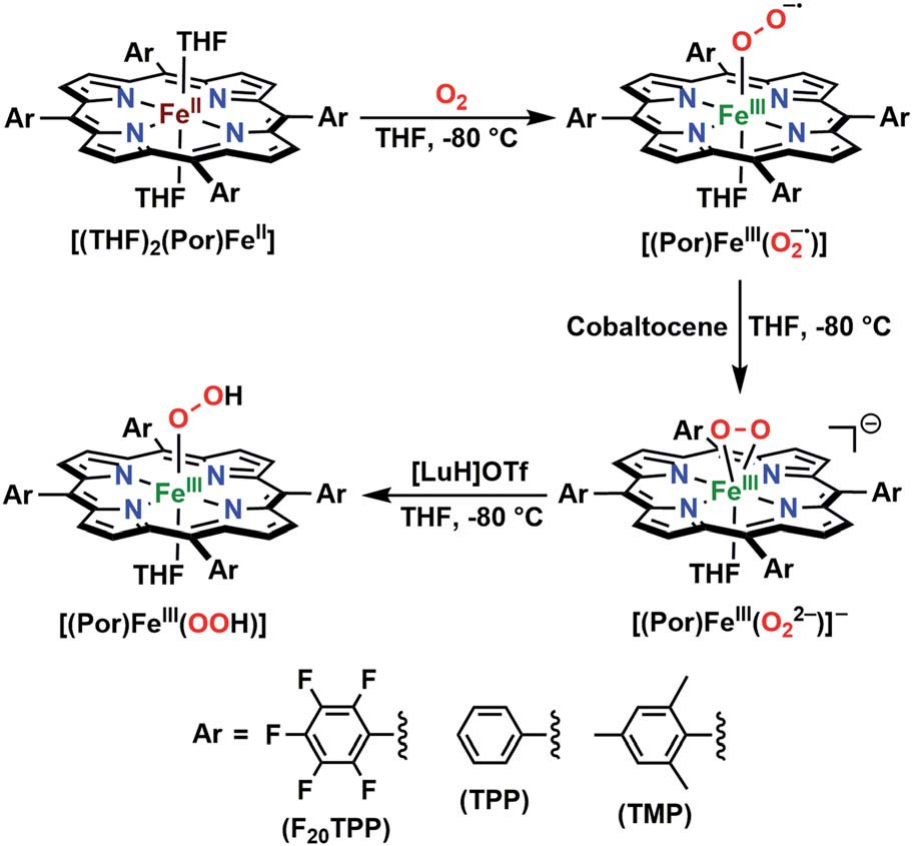
Synthetic protocol for the generation of authentic **[(Por)FeIII(OOH)]** adducts by reduction, followed by protonation of the **[(Por)FeIII(O2−˙)]** complexes. All reactions were carried out in THF at −80 °C.

**Fig. 4:**
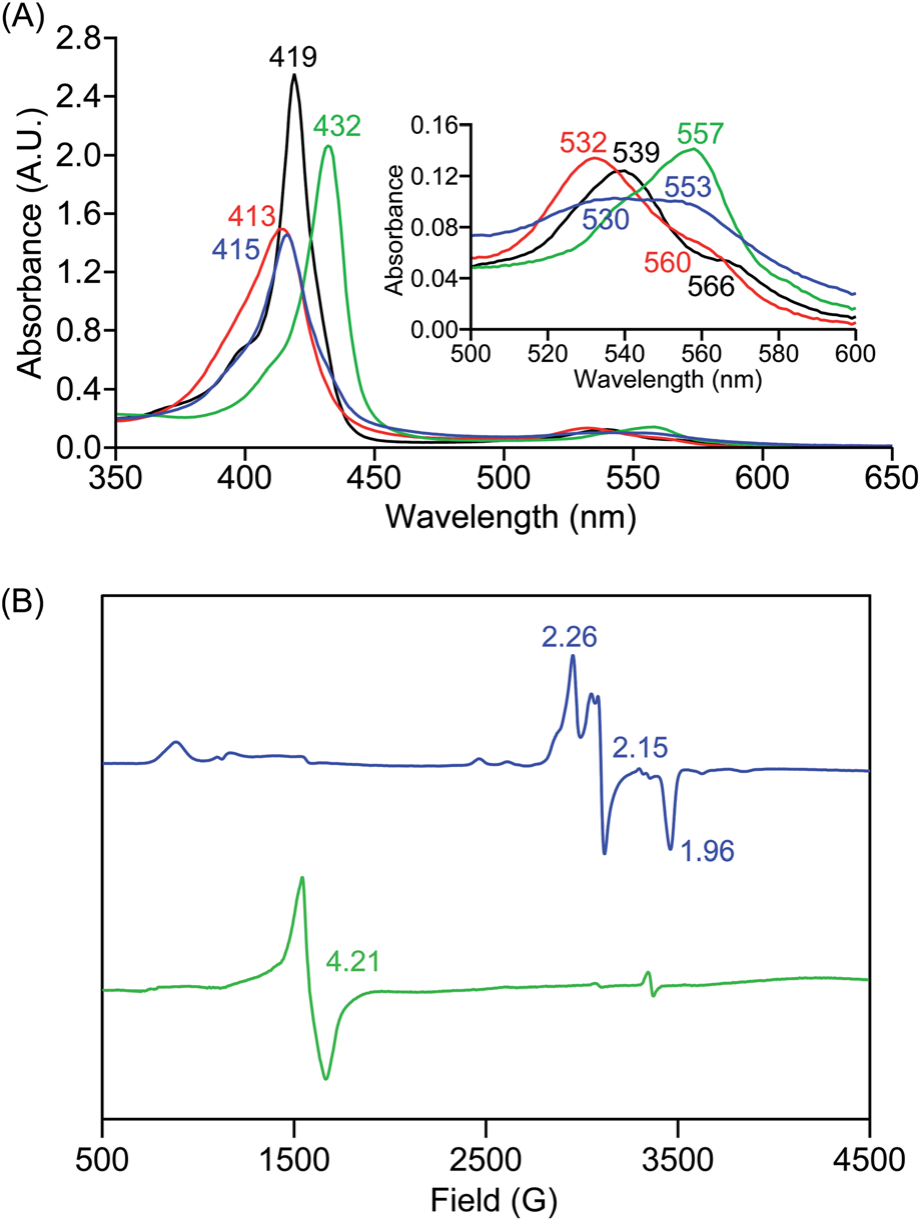
(A) UV-vis spectra for 50 μM solutions of **[(THF)2(F20TPP)FeII]** (black), **[(F20TPP)FeIII(O2−˙)]** (red), **[(F20TPP)FeIII(O22−)]−** (green) and **[(F20TPP)FeIII(OOH)]** (blue) collected in THF at −80 °C, and (B) EPR spectral features (in frozen THF at 7 K) for 2 mM solutions of **[(F20TPP)FeIII(O22−)]−** (green) and **[(F20TPP)FeIII(OOH)]** (blue) complexes.

In further effort to characterize the final **[(Por)FeIII(OOH)]** products, we have generated authentic **[(Por)FeIII(OOH)]** complexes for all three heme systems by reduction, followed by protonation of each superoxo complex (Scheme 2). In that, the one-electron reduction of the **[(Por)FeIII(O2−˙)]** complex was achieved with 1 equiv. of cobaltocene, leading to the corresponding side-on ferric peroxo species, **[(Por)FeIII(O22−)]−** (as would be expected for a five-coordinate or solvent-ligated heme superoxide complex^1*b*^). The peroxo complex was subsequently protonated with 1 equiv. of 2,6-lutidinium triflate ([LuH]OTf) affording the end-on ferric hydroperoxo complex, **[(Por)FeIII(OOH)]**. For example, when 1 equiv. of cobaltocene is added into **[(F20TPP)FeIII(O2−˙)]** in THF at −80 °C, the formation of the side-on ferric heme–peroxo complex, **[(F20TPP)FeIII(O22−)]−** was evidenced by the formation of new electronic absorption features at 432 nm (Soret, *ε* = 2.0 × 10^5^ M^−1^ cm^−1^) and 557 nm (*ε* = 1.5 × 10^4^ M^−1^ cm^−1^) (Fig. 4A; for **[(TPP)FeIII(O2−˙)]** and **[(TMP)FeIII(O2−˙)]** see Fig. S7†).^23*b*,*c*,34^ Characteristic resonance Raman features of **[(F20TPP)FeIII(O22−)]−** were observed at *ν*(Fe–O) = 469 (Δ^18^O_2_ = −15) and *ν*(O–O) = 808 (Δ^18^O_2_ = −41) cm^−1^ (Fig. S8†). Successive addition of 1 equiv. of [LuH]OTf produced new electronic absorption features at 415 nm (Soret, *ε* = 1.5 × 10^5^ M^−1^ cm^−1^), and 553 nm (*ε* = 1.0 × 10^4^ M^−1^ cm^−1^) (Fig. 4A; also see Fig. S7†); the EPR features corresponding to authentic **[(F20TPP)FeIII(O22−)]−** and **[(F20TPP)FeIII(OOH)]** adducts were observed at *g* = 4.21, and *g* = 2.26, 2.15, and 1.96, respectively (Fig. 4B and Table S1†). These electronic absorption and EPR characteristics of our authentic **[(Por)FeIII(OOH)]** species closely resemble those of previously reported heme ferric hydroperoxo adducts,^24*b*,25,26,32^ and manifest unequivocal similarities to those of the PCET reaction products of **[(Por)FeIII(O2−˙)]** and TEMPO–H (Fig. S9 and Table S1†). In summation, the close resemblance of spectroscopic properties of PCET reaction products and authentic hydroperoxo compounds strongly suggest the near stoichiometric conversion of **[(F20TPP)FeIII(O2−˙)]** and **[(TPP)FeIII(O2−˙)]** to **[(F20TPP)FeIII(OOH)]** and **[(TPP)FeIII(OOH)]**, respectively, upon abstracting a H˙ from TEMPO–H under the aforementioned reaction conditions.

### Mechanistic investigations into PCET reactivities of **[(Por)FeIII(O2−˙)]** complexes

**Fig. 5:**
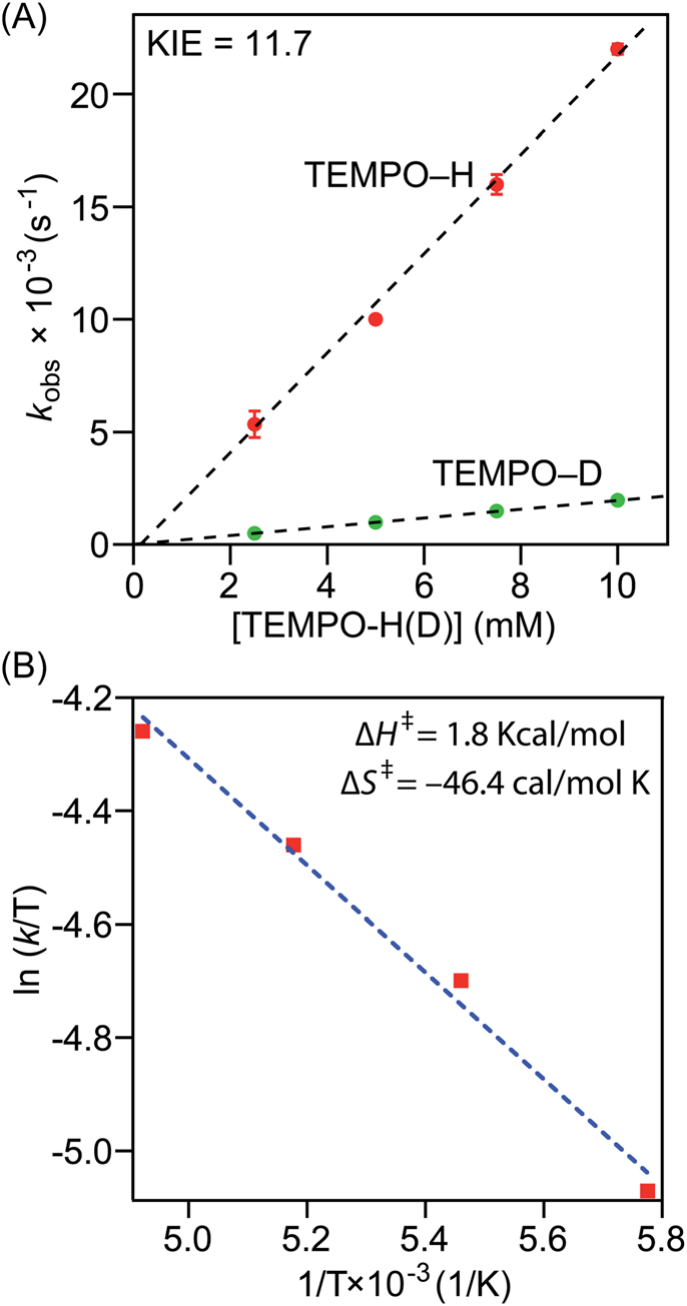
(A) Plot of pseudo-first-order rate constants (*k*_obs_) *versus* [TEMPO–H] (red) or [TEMPO–D] (green) for a 50 μM solution of **[(F20TPP)FeIII(O2−˙)]** in THF at −80 °C. (B) Eyring plot showing ln(*k*/*T*) *versus* 1/*T* for the reaction between a 50 μM THF solution of **[(F20TPP)FeIII(O2−˙)]** and TEMPO–H at −70, −80, −90 and −100 °C.

Precise physicochemical signatures of proton coupled electron transfer reactions mediated by heme superoxide intermediates are gravely understudied; yet could be of momentous importance in a broad combination of applications. We have carried out thorough thermodynamic and kinetic examinations into the entire series of heme superoxide adducts, which exert firm support for the overall experimental findings presented in this study. Pseudo first-order kinetic analyses were carried out by reacting the superoxo complexes with 50−200 equiv. of TEMPO–H in THF at −80 °C (Fig. S10†). All reactions were followed by electronic absorption spectroscopy, and in all cases, the pseudo-first-order rate constants (*k*_obs_) increased linearly as a function of the TEMPO–H substrate concentration (Fig. 5A and S11†), leading to second order rate constants (*k*_2_) of 2.23 M^−1^ s^−1^ and 1.24 M^−1^ s^−1^ for **[(F20TPP)FeIII(O2−˙)]** and **[(TPP)FeIII(O2−˙)]**, respectively (Table 1). As well, kinetic isotope effects (KIE (*k*_H_/*k*_D_)) for PCET mediated by **[(F20TPP)FeIII(O2−˙)]** and **[(TPP)FeIII(O2−˙)]** were found to be 11.7 and 6.7 (Fig. 5A, S11† and Table 1), respectively, using the isotopically labelled substrate, TEMPO–D. It is noteworthy that Karlin and coworkers have observed a similar KIE (6) for **[(F8TPP)FeIII(O2−˙)]**, and values of the same magnitude (4.8–12.1) have been reported for PCET effected by various other non-heme superoxo complexes of Fe, Co, and Cu (Table 1).^29,35,36*a*^ These large (*i.e.*, >2) KIE values present compelling evidence into the rate limiting nature of the homolytic O–H bond cleavage process (and thus, concerted H˙ transfer from the substrate), rather than only proton or electron transfer being the slowest step (where the reaction rate would linearly correlate with either p*K*_a_ or *E*° of the substrate, respectively, rather than the BDFE).^36*b*^ Activation parameters for TEMPO–H oxidation by **[(F20TPP)FeIII(O2−˙)]** and **[(TPP)FeIII(O2−˙)]** were determined *via* Eyring analyses of reaction rates from −70 °C to −100 °C in THF (Fig. 5B and S11†). The experimental Δ*H*^‡^ and Δ*S*^‡^ activation parameter values, and the activation energies (Δ*G*^‡^) derived therefrom are listed in Table 1. Similar Eyring analyses for PCET reactivities of a limited, yet diverse group of non-heme superoxo complexes have been reported, all of which are in the same order of magnitude as the heme superoxo values reported herein.^35*a*–*c*,36*a*^ Notably, Δ*H*^‡^ and Δ*S*^‡^ for most non-heme superoxides are larger than those of heme superoxide adducts; substantially negative Δ*S*^‡^ values of the latter suggest highly ordered transition states as one of the possible contributing factors to their sluggish PCET capabilities. Interestingly, Shaik and coworkers found higher activation barriers for Cyt P450 superoxide mediated hydrogen atom abstraction when compared with the corresponding compound-I oxidant.^37^ These activation parameters also divulge the remarkable influence of the electronic properties of a heme center on its ability to execute successful H˙ atom abstraction. In that, the most electrophilic superoxide adduct, **[(F20TPP)FeIII(O2−˙)]**, exhibits the smallest activation barrier, and thus, reacts the fastest. To the best of our knowledge, this study marks the first report where activation parameters are presented for any substrate reactivity mediated by synthetic heme superoxide intermediates, hence impeding any detailed comparisons with like systems. Nonetheless, these findings impart substantial insights into methodologies for further improvement of ORR efficiencies in fuel cells, and mechanistic understanding of human heme proteins with significant pathological and/or therapeutic value. For example, these activation parameters could shine light on important structural properties of heme ORR catalysts that could be modulated in order to optimally adjust the activation barriers for precise ORR-related reduction and/or protonation events.

**Table 1:** Kinetic and thermodynamic parameters for PCET reactivities of heme and nonheme–superoxo complexes with H-atom donor substrates

Identity of the heme/nonheme superoxo complex^a^	Kinetic parameters					Thermodynamic parameters			Ref.^g^
	*k*_2_ (M^−1^ s^−1^)	KIE (*k*_H_/*k*_D_)	Δ*H*^‡^ (kcal mol^−1^)	Δ*S*^‡^ (cal mol^−1^ K^−1^)	Δ*G*^‡^ (kcal mol^−1^)	*E*° *vs.* Fc^+/0^ (V)^e^	p*K*_a_^f^	BDFE_O–H_^f^ (kcal mol^−1^)	
**[(F20TPP)FeIII(O2−˙)]**	2.23^b^ ± 0.09	11.7^b^	1.8 ± 0.1	−46.4 ± 0.3	10.7 ± 0.2	−1.17^b^^,^^c^ ± 0.01	25.6^b^^,^^c^ ± 0.4	69.1^c^ ± 0.4	This work
**[(TPP)FeIII(O2−˙)]**	1.24^b^ ± 0.09	6.7^b^	2.9 ± 0.2	−42.0 ± 0.4	11.0 ± 0.2	−1.18^b^^,^^c^ ± 0.01	24.6^b^^,^^c^ ± 0.1	67.5^c^ ± 0.2	This work
**[(TMP)FeIII(O2−˙)]**	NR^d^	NR^d^	NR^d^	NR^d^	NR^d^	−1.20^b^^,^^c^ ± 0.01	24.2^b^^,^^c^ ± 0.1	66.5^c^ ± 0.2	This work
**[(F8TPP)FeIII(O2−˙)]**	0.5	6.0	—	—	—	−1.17 ± 0.01	28.8 ± 0.5	73.5 ± 0.9	26*a*
**[(PIm)FeIII(O2−˙)]**	—	—	—	—	—	−1.33 ± 0.01	28.6 ± 0.5	69.5	26*b*
**(TMPImOH)FeIII(O2−˙)**	—	—	—	—	—	−1.32	25.1	—	24*a*
**(TMPImOEt)FeIII(O2−˙)**	—	—	—	—	—	−1.75	32.3	—	24*a*
**[CoIII(py)(O2)(TBP8Cz)]−**	3.6 ± 0.8	9.2	6.7 ± 0.1	−23 ± 0.4	—	—	—	—	35*a*
**Co(O2)(Me3TACN)(S2SiMe2)**	0.87 ± 0.03	8.8	3.6	−36.4	—	—	—	—	35*b*
**[(DMM-tmpa)CuII(O2−˙)]+**	23	11	3.6 ± 0.6	−32 ± 3	—	—	—	—	35*c*
**(TMC)FeIII** superoxo complex	1.2	6.3	—	—	—	—	—	—	35*d*
**FeIII(BDPP)** superoxo complex		7.0							35*e*
**[LCuII-(O2−˙)]+**	1.9	12.1	—	—	—	—	—	—	35*f*
**[FeIII(S2Me2N3(Pr,Pr))(O2)]**	—	4.8	—	—	—	—	—	—	35*g*
μ-1,2-Superoxo Cu^II^_2_ complex	0.13	—	9.03 ± 0.4	26.7 ± 1.6	16.8 ± 0.9	−0.59	22.2	71.7 ± 1.1	36*a*
**[Cu** ^**II**^_**2**_ **(XYLO)(O**_**2**_^**−**^ **˙)]** ^**2+**^	5.6	—	—	—	—	−0.525 ± 0.01	24 ± 0.6	81.8 ± 1.5	29

a See Chart 2 for structures related to this work.

b At −80 °C.

c In THF.

d No reactivity was observed.

e Fc^+/0^ = ferrocene/ferrocenium redox couple.

f Determined for the corresponding hydroperoxo species.

g See reference for experimental conditions.

**Scheme 3:**
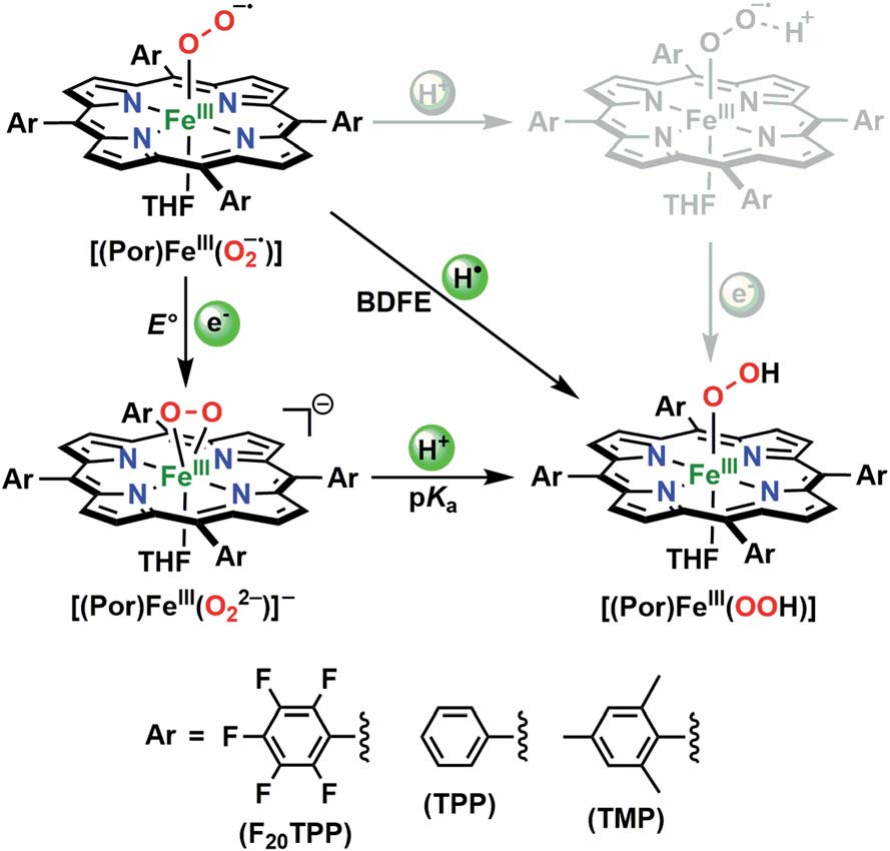
Thermodynamic square scheme used to determine the O–H bond dissociation free energies of the **[(Por)FeIII(OOH)]** complexes in THF, along with the relevant thermodynamic parameters.

Comprehending these observed reactivity disparities on thermodynamic grounds calls for experimental examination of O–H bond dissociation free energies (BDFEs) of the **[(Por)FeIII(OO–H)]** complexes. That is, since the concerted homolytic O–H bond cleavage is the slowest step of the reaction (*vide supra*), O–H bond strength of the **[(Por)FeIII(OO–H)]** product complex predominantly dictates the thermodynamic driving force for the overall reaction. These BDFE values can be deduced in a fairly straightforward fashion using the Bordwell equation (eqn (1)),^27^ if one could determine the redox potential (*E*°) and acidity (p*K*_a_) of the metal oxidants that make up the corresponding thermodynamic cycle/square scheme (Scheme 3).^16*a*,29,36*a*,38^ In addition to shedding light on the perceived reactivities, BDFEs also divulge reactivity limitations to be expected based on the thermodynamic portrayal. All of the heme superoxide complexes employed in this study can be quantitatively converted to the corresponding heme hydroperoxo adducts by stepwise reduction-protonation (by cobaltocene and [LuH]OTf, respectively) reactivity as illustrated in Scheme 2. However, to elucidate the relevant *E*° and p*K*_a_ values experimentally, the equilibrium constants must be determined for each of the reduction and protonation steps.

**Fig. 6:**
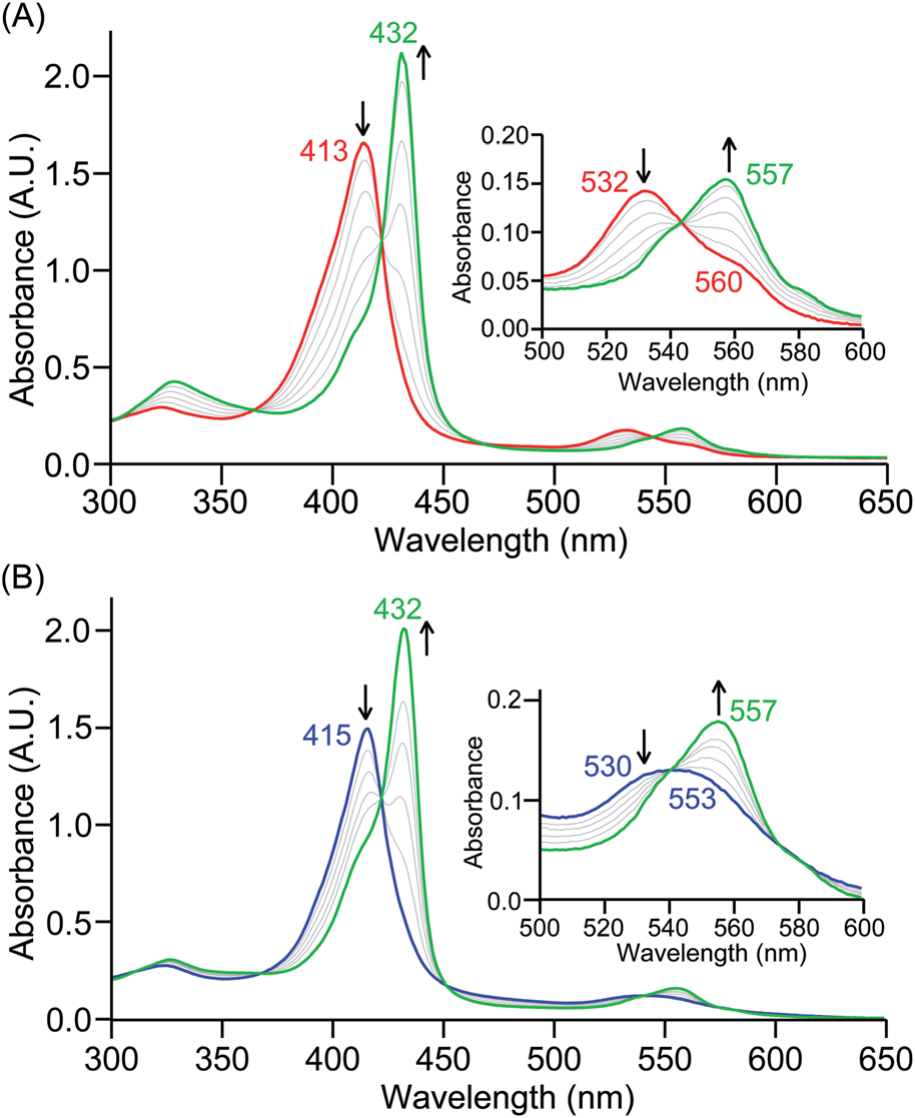
Electronic absorption spectral changes observed (in THF at −80 °C) upon incremental addition of (A) Cr(η^6^-C_6_H_6_)_2_ into **[(F20TPP)FeIII(O2−˙)]** (red) inducing its one-electron reduction to **[(F20TPP)FeIII(O22−)]−** (green), and (B) ^*t*^BuP_2_(dma) into **[(F20TPP)FeIII(OOH)]** (blue) effecting its deprotonation giving **[(F20TPP)FeIII(O22−)]−** (green).

**Scheme 4:**
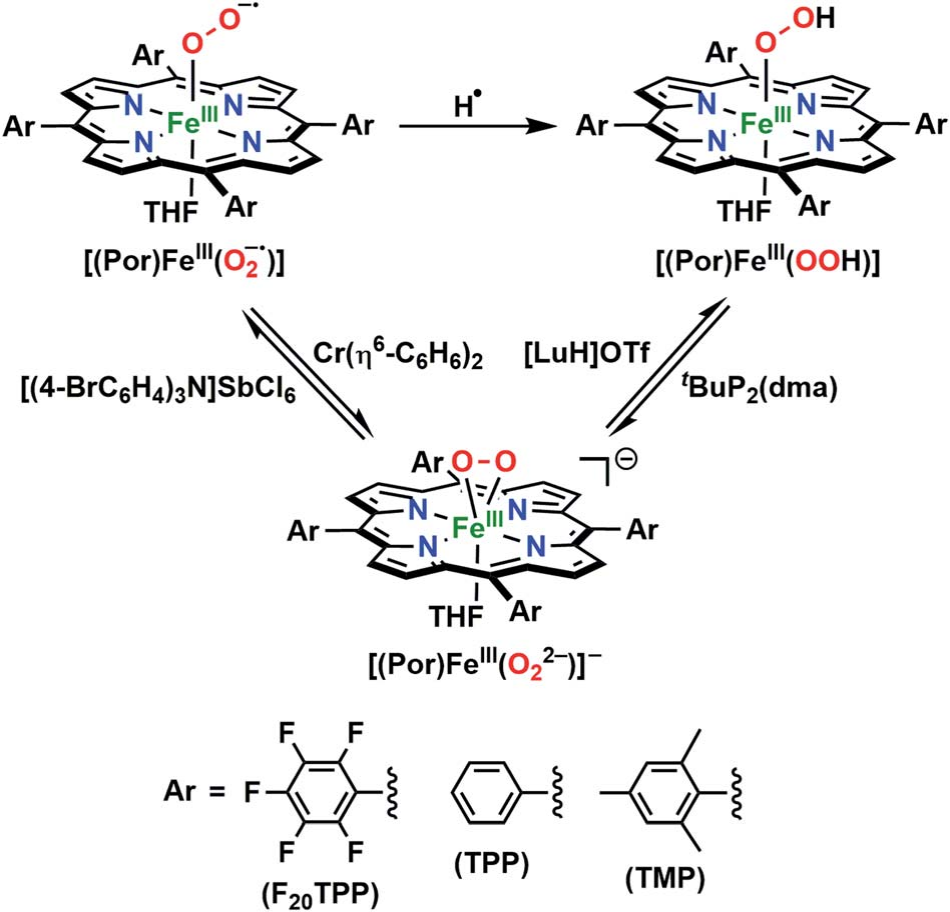
Interconversion of **[(Por)FeIII(O2−˙)]**, **[(Por)FeIII(O22−)]−** and **[(Por)FeIII(OOH)]** complexes in THF at −80 °C, resulting in equilibrium mixtures that allowed the determination of corresponding reduction potential (*E*°) and p*K*_a_ values.

The reduction potential (*E*°) of the **[(Por)FeIII(O2−˙)]**/**[(Por)FeIII(O22−)]−** redox couple for each system was elucidated by titrating the **[(Por)FeIII(O2−˙)]** complexes in THF at −80 °C (Fig. 6A and S12†) with the weak reductant, Cr(η^6^-C_6_H_6_)_2_ (*E*_1/2_ = −1.15 V *vs.* Fc^+/0^ in CH_2_Cl_2_;^39^Scheme 4), to afford equilibrium mixtures of **[(Por)FeIII(O2−˙)]** and **[(Por)FeIII(O22−)]−**. The array of calculated equilibrium constants (Table S2†) yielded corresponding reduction potentials *via* Nernst equation (Table S3†), and were found to be −1.17, −1.18, −1.20 V (*vs.* Fc^+/0^) for **[(F20TPP)FeIII(O2−˙)]**, **[(TPP)FeIII(O2−˙)]**, and **[(TMP)FeIII(O2−˙)]**, respectively (Table 1). Naruta and co-workers have recently reported theoretical reduction potential values for two tethered axial imidazole coordinated TMP-based heme superoxide systems, which are more negative compared to our values (−1.32 and −1.75 V (*vs.* Fc^+/0^ in EtCN))^24*a*^ (Table 1). This trend is consistent with recently reported values for two F_8_TPP based systems by Karlin and coworkers, where the superoxo adduct with tethered axial imidazole coordination displayed a lower reduction potential (−1.33 V)^26*b*^ compared to the parent complex (−1.17 V)^26*a*^ (Table 1). These differences most likely reflect the enrichment of electron density in the d_*z*_^2^ orbital of the iron center upon imidazole ligation, making it much less susceptible for reduction. Notably, our reduction potential for **[(F20TPP)FeIII(O2−˙)]** is in excellent agreement with the electrochemically determined values (−1.09 *vs.* Fc^+/0^ in dimethylformamide at −30 °C) by Anxolabéhère-Mallart and co-workers for the same system.^18,32*b*,40^ Finally, the reversibility of the redox couple, **[(Por)FeIII(O2−˙)]**/**[(Por)FeIII(O22−)]−**, was also established: the **[(Por)FeIII(O22−)]−** complexes generated by one-electron reduction of **[(Por)FeIII(O2−˙)]** adducts by Cr(η^6^-C_6_H_6_)_2_ can be reoxidized stoichiometrically by tris(4-bromophenyl)ammoniumyl hexachloroantimonate ([(4-BrC_6_H_4_)_3_N]SbCl_6_; *E*_1/2_ = 0.67 V *vs.* Fc^+/0^ in MeCN;^39^Scheme 4) giving the starting superoxide complex, which can then be re-reduced to **[(Por)FeIII(O22−)]−** by the addition of 5 equiv. of Cr(η^6^-C_6_H_6_)_2_ (Fig. S13†).

In order to complete the thermodynamic square scheme analysis (Scheme 3), the p*K*_a_ values for the **[(Por)FeIII(OOH)]** complexes were deduced utilizing the derivatized phosphazene weak base, ^*t*^BuP_2_(dma) (p*K*_a_ of conjugate acid = 24.9 in THF at RT^41^), in THF at −80 °C.^42^ Gradual addition of the base afforded equilibrium mixtures of **[(Por)FeIII(OOH)]** and **[(Por)FeIII(O22−)]−** (Fig. 6B and S14†), yielding the corresponding equilibrium constants (Table S4†). Accordingly, p*K*_a_ values of 25.6, 24.6, and 24.2 were computed for **[(F20TPP)FeIII(OOH)]**, **[(TPP)FeIII(OOH)]**, and **[(TMP)FeIII(OOH)]**, respectively (Tables 1 and S5†). These experimentally determined p*K*_a_'s are generally smaller (*i.e.*, more acidic), as would be expected, than those of tethered axial imidazole coordinated TMP-based heme superoxide systems (≈25.1–32.3) previously reported from theoretical studies performed in propionitrile solvent^24*a*^ (Table 1). Moreover, the reversibility of the acid–base interconversion of the **[(Por)FeIII(OOH)]**/**[(Por)FeIII(O22−)]−** couple was established by re-protonation of the aforementioned **[(Por)FeIII(O22−)]−** complexes by [LuH]OTf giving stoichiometric quantities of **[(Por)FeIII(OOH)]** species (Fig. S15†). Finally, our thermochemical findings also parallel those recently reported for **[(F8TPP)FeIII(O2−˙)]** (p*K*_a_ = 28.8; *E*° = −1.17 V) and **[(PIm)FeIII(O2−˙)]** (p*K*_a_ = 28.6; *E*° = −1.33 V) by Karlin and coworkers (Table 1), where similarly reversible protonation and reduction processes were observed.^26^

The substitution of experimental thermodynamic parameters, *E*° and p*K*_a_ (Table 1), in the Bordwell equation (*C*_G(THF)_ = 61 kcal mol; a solvent-dependent constant)^26*a*,43^ lead to BDFE's of 69.1, 67.5, and 66.5 kcal mol^−1^ for the O–H bonds of **[(F20TPP)FeIII(OOH)]**, **[(TPP)FeIII(OOH)]**, and **[(TMP)FeIII(OOH)]**, respectively (Table 1). It is noteworthy that these results are in great accord with previously reported experimental O–H BDFE's by Karlin and coworkers (73.5, 69.5 kcal mol; Table 1), as well as computationally calculated BDEs of several heme hydroperoxide complexes by Morokuma and coworkers (64–66 kcal mol^−1^),^44^ Moreover, our BDFE values are in excellent agreement with the observed PCET reactivity pattern, where **[(F20TPP)FeIII(O2−˙)]** and **[(TPP)FeIII(O2−˙)]** facilitate hydrogen atom abstraction from TEMPO–H (BDFE_heme hydroperoxo_ > BDFE_TEMPOH_), while **[(TMP)FeIII(O2−˙)]** remained unreactive (BDFE_heme hydroperoxo_ ≈ BDFE_TEMPOH_; *i.e.*, thermodynamically neutral). Finally, these BDFEs also unveil the thermodynamic basis for the incapacity of hydrogen atom abstraction by **[(Por)FeIII(O2−˙)]** complexes from other substrates with stronger (*i.e.*, BDFE ≥ 69.1 kcal mol^−1^) C–H, N–H, or O–H bonds.

### Computational studies

**Fig. 7:**
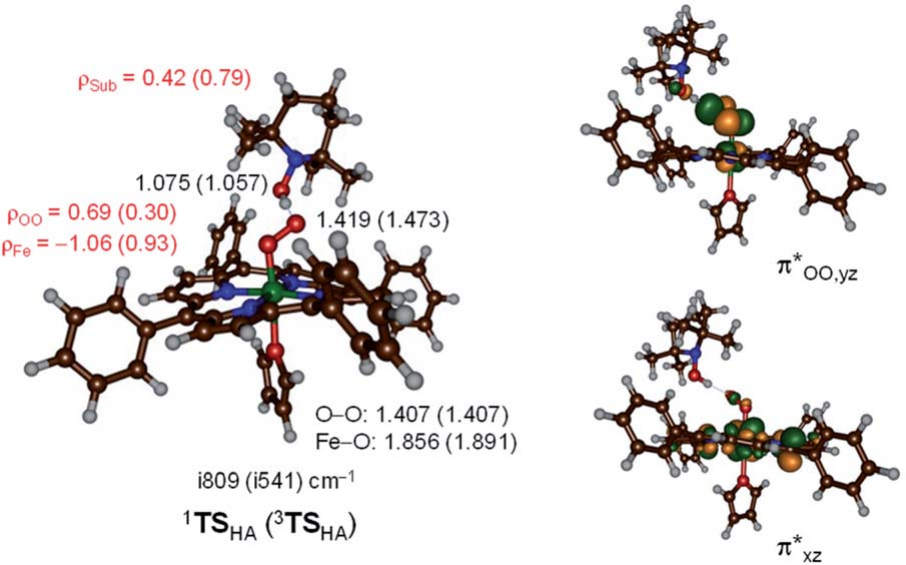
Optimized geometries of the hydrogen atom abstraction transition states from TEMPO–H by **[(TPP)FeIII(O2−˙)]** in the singlet and triplet spin states. Bond lengths are in angstroms, the imaginary frequency in cm^−1^ and group spin densities (*ρ*) in atomic units. The right-hand-side shows the singly occupied molecular orbitals in the singlet and triplet reactants.

To gain further insight into the intricate electrochemical properties of the iron(iii)–superoxo complexes, we did a computational study on both **[(F20TPP)FeIII(O2−˙)]** and **[(TPP)FeIII(O2−˙)]** systems and studied their reactivities with TEMPO–H. The **[(TPP)FeIII(O2−˙)]** complex is calculated as an end-on superoxo configuration with an open-shell singlet spin ground state with two unpaired electrons antiferromagnetically coupled in 
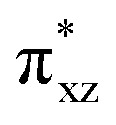
 and 
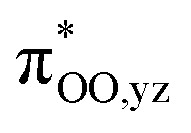
 orbitals. The former orbital is a dominant 3d_xz_ atomic orbital on iron in the plane of the FeOO group, while the latter is the antibonding interaction along the O–O bond that also interacts with the 3d_yz_ orbital on iron. The triplet spin state has the same electron configuration but ferromagnetically coupled and its energy with zero-point energy correction is Δ*E* + ZPE = 5.6 kcal mol^−1^ higher in energy. Our calculated ground state; therefore, matches experimental assignments (see above) that the system is EPR silent. Subsequently, we calculated the hydrogen atom abstraction transition states (**TS**_HA_) for the reaction of ^1,3^**[(TPP)FeIII(O2−˙)]** from TEMPO–H and the optimized geometries are shown in Fig. 7. The reactions are concerted with a single hydrogen atom transfer leading to an iron(iii)–hydroperoxo product. The transition states are early with short TEMPO–H distances of 1.075 and 1.057 Å in the singlet and triplet spin states, respectively. At the same time, the accepting O–H distance is long: 1.419 Å in ^1^**TS**_HA_ and 1.473 Å in ^3^**TS**_HA_. Generally, early transition states correspond with low-energy hydrogen atom abstraction reactions, while later transition states have much higher energy barriers.^45^

Hydrogen atom abstraction by ^1,3^**[(TPP)FeIII(O2−˙)]** leads to electron transfer into the 
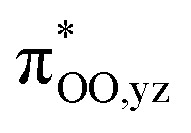
 orbital and generates a doublet spin **[(TPP)FeIII(OOH)]** coupled to a TEMPO radical. This electron transfer is confirmed by the group spin densities that show an increase of spin on the TEMPO group to *ρ*_Sub_ = 0.42, while at the same time spin is lost on the dioxygen moiety *ρ*_OO_ = 0.69. The hydrogen atom abstraction barrier is Δ*E*^‡^ + ZPE = 3.2 kcal mol^−1^ in the singlet spin state and 9.9 kcal mol^−1^ for the triplet spin state barrier. The low-spin barrier matches the experimentally determined Δ*H*^‡^ = 2.9 kcal mol^−1^ excellently. The experimentally determined entropy contribution is relatively large, probably due to a solvent cage surrounding the molecular complex. As shown by previous work of ours, reactions in solution often have a solvent cage around the active complex that affect entropies and particularly reduces vibrational contributions.^46^ Because of the fact that the solvent cage was not included in the model, the computational free energy of activation is relatively low and much lower than that observed in experiment as the gas-phase entropy is overestimated.

The imaginary frequency in the transition state is modest: i809 cm^−1^ in ^1^**TS**_HA_ and i541 cm^−1^ in ^3^**TS**_HA_. Typical values for hydrogen atom abstraction imaginary frequencies are of the order of i1200–i1800 cm^−1^ and implicate a narrow and sharp peak on the potential energy surface.^47^ The smaller values seen here, also imply that quantum mechanical tunnelling will be less. Indeed, we calculated a KIE = 3.6 using the Eyring model and KIE = 4.0 with the Wigner tunnelling model for the reaction that passes ^1^**TS**_HA_. By contrast, for hydrogen atom abstraction of aliphatic substrates by P450 compound I or non-heme iron dioxygenases typically values well larger than 12 are calculated.^48^ Our obtained KIE value is in good agreement with the experimentally determined value of 6.7 (Table 1) and hence the calculations give a similar potential energy landscape and curvature as derived from the experimental work.

**Fig. 8:**
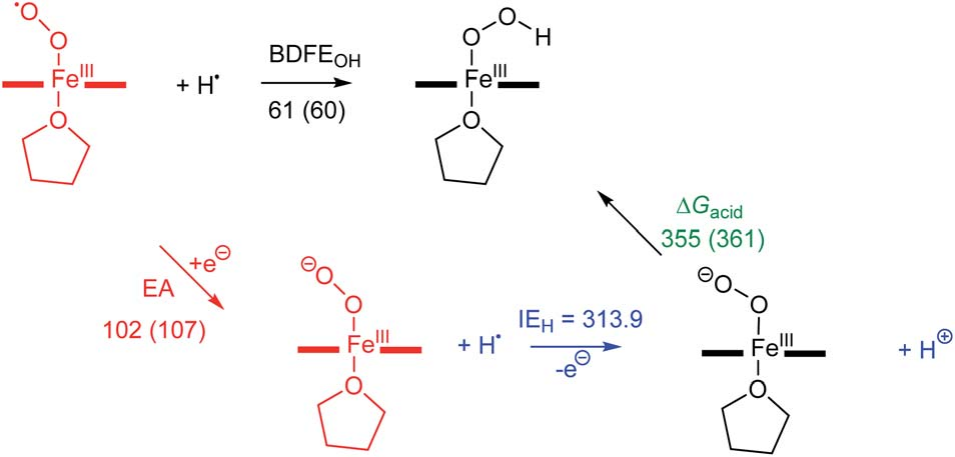
Thermochemical cycle for **[(TPP)FeIII(O2−˙)]** (and **[(F20TPP)FeIII(O2−˙)]** in parenthesis) with calculated reaction energies for electron transfer (EA), O–H bond dissociation and gas-phase acidity. All data in kcal mol^−1^.

Next, we calculated the BDFE of **[(TPP)FeIII(OOH)]** as the energy difference of its optimized geometry with that of a hydrogen atom and the **[(TPP)FeIII(O2−˙)]** complex and find a value of 61 kcal mol^−1^ as an energy difference between the singlet spin iron–superoxo and the doublet spin iron(iii)–hydroperoxo complex and a hydrogen atom (Fig. 8). Our calculated value matches the experimental derivation from redox potential and p*K*_a_ values well and shows the hydroperoxo O–H bond is relatively weak. Thus, the BDFE of porphyrin/heme ligated iron(iii)–hydroxo complexes were calculated for several systems previously. A value of 80.8 kcal mol^−1^ was obtained for model of horseradish peroxidase that has a porphyrin equatorial ligand and imidazole as axial ligand, while a value of 88.9 kcal mol^−1^ was found for a P450 model with a thiolate axial ligand.^49^ As often the BDFE represents a measurement of the ability of an oxidant to abstract hydrogen atoms efficiently, this means that **[(TPP)FeIII(O2−˙)]** will be a weak oxidant and only able to activate substrates with weak C–H or O–H bonds. Indeed, as reported above, our experimental studies show that only reactions with TEMPO–H led to hydrogen atom transfer, while the system is inactive with other aliphatic substrates. These results; therefore, support previous computational studies on the oxidative properties of the iron(iii)–hydroperoxo and iron(iii)–superoxo intermediates in P450 enzymes that found them to be sluggish oxidants.^37^ In addition to the BDFE values, we also calculated the one-electron reduction potential (or electron affinity, EA) of the iron(iii)–superoxo complex and find values of 102 and 107 kcal mol^−1^ for **[(TPP)FeIII(O2−˙)]** and **[(F20TPP)FeIII(O2−˙)]**, respectively. Finally, we estimated the gas-phase acidity of the iron(iii)–hydroperoxo complex (Δ*G*_acid_) from the experimentally determined ionization energy of a hydrogen atom (IE_H_ = 313.9 kcal mol^−1^)^50^ and the difference between EA and BDFE_OH_.

## Conclusions

Despite the ubiquitous nature of proton-coupled electron transfer processes mediated by heme superoxo adducts in both biology and alternative energy applications, understanding of precise reactivity limitations in terms of key physicochemical properties of the heme oxidant is still in its infancy. To this end, we present a detailed thermodynamic, kinetic, and theoretical analysis of PCET reactivities of a series of electronically disparate, structurally equivalent heme superoxo model systems. These heme ferric superoxo adducts, **[(Por)FeIII(O2−˙)]**, abstract a hydrogen atom (H˙; *i.e.*, H^+^ + e^−^) from the weak O–H bond substrate TEMPO–H, in a single kinetic step (*i.e.*, concerted) leading to the stoichiometric generation of the corresponding heme ferric hydroperoxo, **[(Por)FeIII(OOH)]**, species and TEMPO˙ radical, where the substrate O–H bond cleavage is the overall rate-determining process. Accordingly, H/D KIE's were found to be 11.7 and 6.7 (Table 1) for **[(F20TPP)FeIII(O2−˙)]** and **[(TPP)FeIII(O2−˙)]** adducts (Chart 2), respectively, which are in excellent agreement with our theoretical findings. All heme reactants and products have been fully characterized using electronic absorption, EPR, resonance Raman, and ^2^H NMR spectroscopies under cryogenic conditions. Besides, the identities and yields of the resultant **[(Por)FeIII(OOH)]** complexes have been probed by the comparison of their spectroscopic features with those of the independently prepared (*i.e.*, by reduction–protonation of the **[(Por)FeIII(O2−˙)]** counterpart) complexes, and by quantification of hydrogen peroxide liberated upon acidification, respectively.

Variable temperature kinetic (Eyring) studies have allowed the ascertainment of activation parameters (*i.e.*, Δ*H*^‡^, Δ*S*^‡^, and Δ*G*^‡^) that dictate the aforementioned PCET reactivities of **[(Por)FeIII(O2−˙)]** complexes, which are in strong support of their second-order PCET rates. Indeed, computational findings are in great agreement within the limitations of the employed solvation model. Notably, this study marks the first report with both experimental and theoretical insights into activation parameters of any substrate reactivity of synthetic heme superoxo systems, which, in this case, could be monumental in the rational design of oxygen reduction catalysts for fuel cell or similar applications. To gain further understanding into the strong influence of heme center electronics on the competency to abstract an H˙ by the ligated superoxide unit, we have determined O–H BDFE's for the entire series of **[(Por)FeIII(OOH)]** complexes utilizing experimental *E*° and p*K*_a_ values deduced from redox and acid–base titrations (Table 1), respectively. The O–H BDFE's of 69.1, 67.5, and 66.5 kcal mol^−1^ found for **[(F20TPP)FeIII(OOH)]**, **[(TPP)FeIII(OOH)]**, and **[(TMP)FeIII(OOH)]**, respectively, provide strong thermodynamic evidence in support of the observed limitations in reactivity. That is, **[(F20TPP)FeIII(O2−˙)]** and **[(TPP)FeIII(O2−˙)]** successfully abstracted an H˙ from TEMPO–H, while **[(TMP)FeIII(O2−˙)]** did not react due to the nullified thermodynamic driving force (*i.e.*, BDFE_heme hydroperoxo_ ≈ BDFE_TEMPOH_). Moreover, the trends in experimentally observed O–H BDFE's are unequivocally supported by our computational results. Lastly, this work reveals previously unknown, critical aspects surrounding electronically driven feasibilities of substrate reactivities facilitated by heme superoxo intermediates. This knowledge will broaden the current understanding of long overlooked reactivity properties of mid-valent heme–oxygen intermediates in biology, while offering novel avenues for the design of better ORR catalysts leading to enhanced efficiencies (*e.g.*, fine-tuning of (1) heme systems steered by electronic properties, (2) acidities of proton sources utilized in ORR catalysis, and (3) structure-based thermodynamic (*e.g.*, p*K*_a_, *E*°, BDFE) and kinetic (*e.g.*, reaction rates and feasibilities, rate limiting events) properties of heme ORR catalysts to further the overall efficacy *etc.*). If geometric, electronic, and/or secondary sphere properties of heme superoxide intermediates can be optimized to promote the oxidation of stronger organic substrates is an intriguing unknown, and will be a focus of our future interrogations.

## Data availability

The datasets supporting this article have been uploaded as part of the supplementary material.

## Author contributions

G. B. W. conceived the project idea and supervised the investigation; P. M. and C. L. synthesized all compounds, and generated and analyzed the experimental data; I. I. and S.-R. Y. conducted and analyzed the resonance Raman characterization experiments; E. F. G. and S. d. V. carried out theoretical computations and interpreted the data. G. B. W., S. d. V. and P. M. wrote and edited the manuscript with input from all other co-authors.

## Conflicts of interest

There are no conflicts to declare.

## Supplementary Material

SC-012-D1SC01952J-s001
