# Supplementary material for: Proton-coupled electron transfer reactivities of electronically divergent heme superoxide intermediates: a kinetic, thermodynamic, and theoretical study
Source: Chem Sci. 2021 May 27;12(25):8872–83. doi: 10.1039/d1sc01952j (PMC8246096; doi:10.1039/d1sc01952j)
Supplement: SC-012-D1SC01952J-s001 [file SC-012-D1SC01952J-s001.pdf]

Electronic Supplementary Material (ESI) for Chemical Science.
This journal is © The Royal Society of Chemistry 2021

Supporting Information for

Proton-coupled Electron Transfer Reactivities of Electronically Divergent Heme Superoxide Intermediates: A Kinetic, Thermodynamic, and Theoretical Study

Pritam Mondal,[a] Izumi Ishigami,[b] Emilie F. Gérard,[c] Chaeeun Lim,[a] Syun-Ru Yeh,[b] Sam P. de Visser,*[c] and Gayan B. Wijeratne*[a]

[a] Department of Chemistry, University of Alabama at Birmingham, Birmingham, AL 35205, United States. E-mail: wijeratne@uab.edu.

[b] Department of Physiology and Biophysics, Albert Einstein College of Medicine, The Bronx, New York 10461, United States. E-mail: syun-ru.yeh@einsteinmed.org

[c] Manchester Institute of Biotechnology and the Department of Chemical Engineering and Analytical Science, The University of Manchester, 131 Princess Street, Manchester M1 7DN, UK. E-mail: sam.devisser@manchester.ac.uk

Contents	Page
**1. Materials and methods**	S3
**2. Formation of the heme superoxo, [(Por)Fe^{III}($O_2^{-•}$)] complexes, where Por = porphyrinate supporting ligand**	S5
Figure S1. UV−vis spectra for the formation of **[(Por)Fe^{III}($O_2^{-•}$)]** in THF at −80 °C.	S5
Figure S2. Resonance Raman spectra of the **[(F_{20}TPP)Fe^{III}($O_2^{-•}$)]** complex	S6
**3. Low-temperature ^{2}H NMR spectroscopic studies**	S6
**4. EPR and resonance Raman spectroscopic studies**	S6
Figure S3. EPR spectral data (in frozen THF at 7 K) for the reaction of **[(F_{20}TPP)Fe^{III}($O_2^{-•}$)]** with TEMPO–H.	S7
Figure S4. EPR spectral data (in frozen THF at 7 K) for the reaction of **[(TPP)Fe^{III}($O_2^{-•}$)]** with TEMPO–H.	S8
Figure S5. Resonance Raman spectra for the final heme product of the reaction between **[(TPP)Fe^{III}($O_2^{-•}$)]** and TEMPO–H.	S8
**5. Hydrogen peroxide quantification**	S9
Figure S6. H_2O_2 quantification via UV–vis titrations in support of the formation and yields of the **[(Por)Fe^{III}(OOH)]** product complexes.	S9
**6. Generation of authentic heme peroxo, [(Por)Fe^{III}(O_2^{2-})]$^{-}$ and heme hydroperoxo, [(Por)Fe^{III}(OOH)] complexes (Por = porphyrinate supporting ligand)**	S10
Figure S7. UV−vis spectra for the formation of authentic **[(Por)Fe^{III}(O_2^{2-})]$^{-}$ and [(Por)Fe^{III}(OOH)]** complexes in THF at −80 °C.	S10
Figure S8. Resonance Raman spectra for the **[(F_{20}TPP)Fe^{III}(O_2^{2-})]$^{-}$** complex	S11
Figure S9. UV-vis spectral comparison of final heme product of the reaction between **[(F_{20}TPP)Fe^{III}($O_2^{-•}$)]** and TEMPO–H with authentic **[(F_{20}TPP)Fe^{III}(OOH)]** complex.	S11
Table S1. Table for spectroscopic characterization of **[(F_{20}TPP)Fe^{III}($O_2^{-•}$)]**, **[(F_{20}TPP)Fe^{III}(O_2^{2-})]$^{-}$**, and **[(F_{20}TPP)Fe^{III}(OOH)]** complexes.	S11
**7. Spectroscopic reactivity and kinetic studies of [(Por)Fe^{III}($O_2^{-•}$)] complexes with TEMPO–H(D)**	S12
Figure S10. Plots of the change in absorbance versus time (time traces) of **[(Por)Fe^{III}($O_2^{-•}$)]** with 100 and 150 equiv of TEMPO–H in THF at -80 °C.	S12
Figure S11. Plot of pseudo-first-order rate constants versus [TEMPO–H(D)] for **[(TPP)Fe^{III}($O_2^{-•}$)]** and variable temperature Eyring plot for the reaction of **[(TPP)Fe^{III}($O_2^{-•}$)]** with TEMPO–H.	S13
**8. Determination of reduction potentials of [(Por)Fe^{III}($O_2^{-•}$)] complexes**	S14
Table S2. Calculation of equilibrium concentrations for the titration of Cr(η^6-C_6H_6)$_2$ with **[(Por)Fe^{III}($O_2^{-•}$)]** complexes in THF at –80 °C.	S14
Table S3. Calculations for the determination of reduction potentials of **[(Por)Fe^{III}($O_2^{-•}$)]** complexes in THF at –80 °C.	S15
Figure S12. UV−vis spectral changes (in THF at –80 °C) during the conversion of **[(Por)Fe^{III}($O_2^{-•}$)]** to **[(Por)Fe^{III}(O_2^{2-})]$^{-}$** using Cr(η^6-C_6H_6)$_2$.	S16
Figure S13. UV−vis spectral changes (in THF at –80 °C) during the reversible interconversion of **[(Por)Fe^{III}(O_2^{2-})]$^{-}$** and **[(Por)Fe^{III}($O_2^{-•}$)]** complexes using ([(4-BrC$_6$H$_4$)$_3$N]SbCl$_6$) and Cr(η^6-C_6H_6)$_2$.	S17
**9. Determination of pK_a values of [(Por)Fe^{III}(OOH)] complexes**	S18
Table S4. Calculation of equilibrium concentrations for the titration of **[(Por)Fe^{III}(OOH)]** complexes with ^{t}BuP$_2$(dma) in THF at –80 °C.	S18
Table S5. Calculation of pK_a values of **[(Por)Fe^{III}(OOH)]** complexes in THF at –80 °C.	S19
Figure S14. UV−vis spectral changes (in THF at –80 °C) during the conversion of **[(Por)Fe^{III}(OOH)]** to **[(Por)Fe^{III}(O_2^{2-})]$^{-}$** using ^{t}BuP$_2$(dma).	S19
Figure S15. UV−vis spectral changes (in THF at –80 °C) during the reversible interconversion of **[(Por)Fe^{III}(OOH)]** and **[(Por)Fe^{III}(O_2^{2-})]$^{-}$** complexes using ^{t}BuP$_2$(dma) and [LuH]OTf.	S20
**10. Tables for computational calculations**	S21
**11. Cartesian coordinates**	S26
**12. References**	S40

1. Materials and methods

All commercially available chemicals were purchased at the highest available purity, and were used as received unless otherwise stated. Air-sensitive compounds were handled either under an argon atmosphere using standard Schlenk techniques, or in an Mbraun Unilab Pro SP (<0.1 ppm O_2, <0.1 ppm H_2O) nitrogen-filled glovebox. All organic solvents were purchased at HPLC-grade or better. THF was degassed (bubbling argon gas for 30 min at room temperature) and dried (passing through a 60 cm alumina column) using an Inert Pure Solv MD 5 (2018) solvent purification system, and then stored in dark glass bottles inside the glovebox over 4 Å molecular sieves at least for 72 hrs prior to use. The purity of O_2 gas used was >99%, and was further purified by passage through a 12 in. column containing drierite and 5 Å molecular sieves. Benchtop UV–vis experiments were carried out using Agilent Cary 60 spectrophotometer equipped with a liquid nitrogen chilled Unisoku CoolSpek UV USP-203-B cryostat. A 2 mm path length quartz cell cuvette modified with an extended glass neck with a female 14/19 joint and stopcock was used to perform all UV–vis experiments. Low-temperature ^{2}H NMR spectroscopic studies were carried out on a Bruker AV 360 MHz NMR Spectrometer. All spectra were recorded in 5 mm (outer diameter) NMR tubes. The chemical shifts were reported as δ (ppm) values calibrated to natural abundance deuterium or proton solvent peaks. Electron paramagnetic resonance (EPR) spectra were collected in 4 mm (outer diameter) quartz tubes using an X-band Bruker EMX-plus spectrometer coupled to a Bruker ER 041 XG microwave bridge, and a continuous-flow liquid helium cryostat (ESR900) controlled by an Oxford Instruments TC503 temperature controller (Experimental conditions: microwave frequency = 9.41 GHz; microwave power = 0.2 mW; modulation frequency = 100 kHz; modulation amplitude = 10 G; temperature = 7 K). For Raman spectroscopic measurements, each frozen sample in an EPR tube was immersed in a EPR Cold Finger Liquid Nitrogen Dewar. It was kept rotating by a sample spinner (Princeton Photonics, Inc., Princeton, NJ) during the data acquisition to avoid photoinduced artifacts. The 413.1 nm excitation beam from a Kr ion laser (Coherent, Santa Clara, CA) was focused on the spinning EPR tube by a cylindrical lens. The back scattered light was collected by a camera lens and focused on the 100-μm wide entrance slit of a 1.25 m Spex spectrometer equipped with a 1200 grooves/mm grating (Horiba Jobin Yvon, Edison, NJ), where it was dispersed and then detected by a liquid-nitrogen cooled CCD detector (Princeton Instruments, Trenton, NJ). A holographic notch filter (Kaiser, Ann Arbor, MI) was used to remove the excitation light. The laser power was 2 mW

and the acquisition time was 20 min. The Raman shift was calibrated by using indene as the reference. The data were processed using WinSpec/32 (Princeton Photonics, Inc., Princeton, NJ) and IGOR Pro (WaveMetrics Inc., Portland, OR). 5,10,15,20-tetraphenylporphyrin iron(III) chloride, **[(TPP)Fe^{III}Cl]** was purchased from commercial sources. The syntheses of **H_2(F_{20}TPP)**,[1] **H_2(F_{20}TPP)-*d_8***,[2] **H_2(TMP)**,[3] TEMPO–H,[4] TEMPO–D[4], [LuH]OTf[5] were carried out according to previously published methods. Metalation of the porphyrinates to generate **[(F_{20}TPP)Fe^{III}Cl]**, **[(F_{20}TPP-*d_8*)Fe^{III}Cl]**, **[(TMP)Fe^{III}Cl]** and the subsequent reduction to **[(THF)$_2$(Por)Fe^{II}]** complexes were carried out by following previously reported procedures.[6] Density functional theory calculations were performed on **[(TPP)Fe^{III}($O_2^{-\bullet}$)]** and **[(F_{20}TPP)Fe^{III}($O_2^{-\bullet}$)]** and their reactivities with TEMPO-H. All calculations were done with the Gaussian-09 software package[7] at the unrestricted B3LYP level of theory.[8] Geometries were optimized without constraints and transition states were characterized with a single imaginary frequency for the correct mode. In addition, intrinsic reaction coordinate scans from the transition states were performed, which confirmed they connected to the assigned reactants and products complexes. Initial geometry optimizations utilized an LANL2DZ basis set (with core potential) on iron and 6-31G on the rest of the atoms (basis set BS1)[9] and a solvent model (self-consistent reaction field) using the continuum polarized conductor model with a dielectric constant mimicking THF.[10] Subsequent single point calculations using the LACV3P+ basis set (with core potential) and 6-311+G* on the rest of the atoms were done in Gaussian: basis set BS2. Analytical frequencies were calculated at −80 °C and used to evaluate zero point energies, vibrational entropies and kinetic isotope effects.

2. Formation of the heme superoxo, $[(Por)Fe^{III}(O_2^{-\bullet})]$ complexes, where Por = porphyrinate supporting ligand

Generation of the heme superoxo complexes, $\mathbf{[(Por)Fe^{III}(O_2^{-\bullet})]}$, was carried out following a literature-adapted procedure.[6] In a typical experiment, a 50 μM THF solution (1 mL) of $\mathbf{[(THF)_2(Por)Fe^{II}]}$ was added into a 2 mm pathlength Schlenk cuvette inside the glovebox, and was sealed using a rubber septum. Upon cooling down inside the UV-vis cryostat stabilized at –80 ºC, this solution was bubbled with dry dioxygen gas using a needle, and excess $O_{2(g)}$ was removed by three vacuum/Ar purge cycles. The complete formation of the superoxide complexes, $\mathbf{[(Por)Fe^{III}(O_2^{-\bullet})]}$, was monitored by UV–vis spectroscopy (Figure S1).

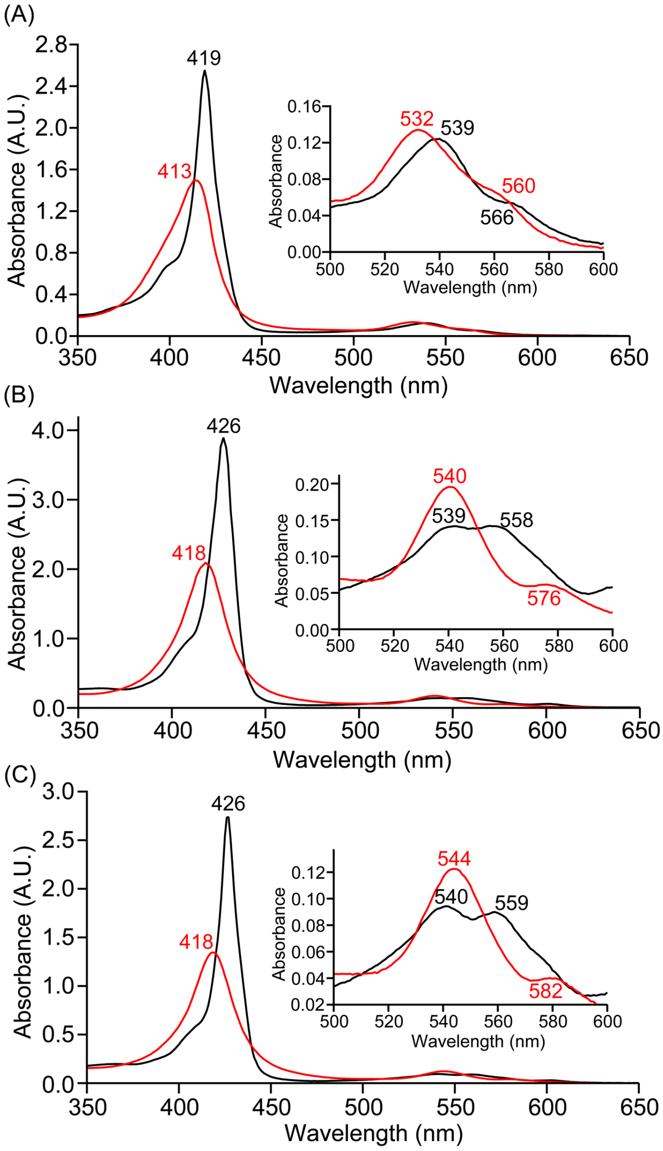

Figure S1. UV−vis spectra (in THF at −80 °C) for the formation of (A) $\mathbf{[(F_{20}TPP)Fe^{III}(O_2^{-\bullet})]}$, (B) $\mathbf{[(TPP)Fe^{III}(O_2^{-\bullet})]}$, and (C) $\mathbf{[(TMP)Fe^{III}(O_2^{-\bullet})]}$. Black: $\mathbf{[(THF)_2(Por)Fe^{II}]}$ and red: $\mathbf{[(Por)Fe^{III}(O_2^{-\bullet})]}$. Insets show the expanded Q-band regions.

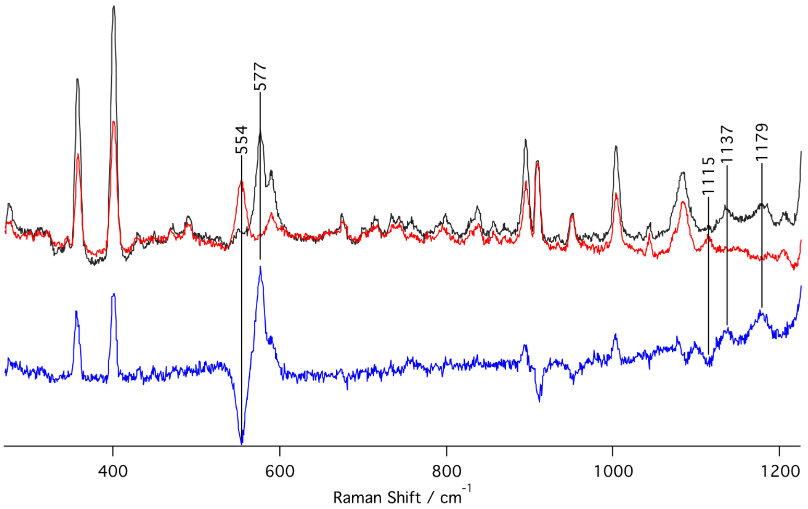

Figure S2. Resonance Raman spectra (λ_{ex} = 413.1 nm) collected for a 2 mM frozen THF solution of **[(F$_{20}$TPP)Fe^{III}(O$_2$$^{-•}$)]** prepared with $^{16}O_{2(g)}$ (black) and $^{18}O_{2(g)}$ (red).

3. Low-temperature ^{2}H NMR spectroscopic studies

For a typical ^{2}H NMR experiment, 15 mg of **[(F$_{20}$TPP-*d$_8$*)Fe^{II}]** (15 mM) was dissolved in 0.5 mL of THF, and was sealed in a 5 mm (outer diameter) NMR tube within the glovebox. This tube was then stabilized at –90 °C using a liquid nitrogen/acetone cold bath, followed by the addition of $O_{2(g)}$ by means of a 9" needle. TEMPO–H (50 equiv in 50 µL THF) was then added using a Hamilton gas-tight syringe, and was quickly mixed with Ar bubbling and kept for 20 min. The tube was then transferred into the cryostat of the NMR spectrometer held at –80 °C (Figure 2).

4. EPR and resonance Raman spectroscopic studies

In a typical resonance Raman sample preparation, 100 µL of the ferrous heme complex, **[(THF)$_2$(Por)Fe^{II}]** (2 mM in THF), was placed in a 9 mm EPR tube (4 mm O.D.) and was sealed with a rubber septum inside the glovebox. Following cooling to −80 °C (using liquid nitrogen/acetone cold bath), dry $O_{2(g)}$ (or $^{18}O_{2(g)}$) was bubbled through the solution using a three-way gastight syringe to generate the corresponding superoxide complex, **[(Por)Fe^{III}(O$_2$$^{-•}$)],** under inert conditions. Subsequently, 50 equiv of TEMPO–H was added in, and the reaction mixture was homogenized with dry Ar bubbling. The tube was then kept at −80 °C for 20 min to complete the reaction. Authentic **[(Por)Fe^{III}(O$_2$$^{2-}$)]$^-$** and **[(Por)Fe^{III}(OOH)]** complexes were prepared by adding 1 equiv of cobaltocene (10 µL) into **[(Por)Fe^{III}(O$_2$$^{-•}$)]**, and 1 equiv of [LuH]OTf (10 µL) into **[(Por)Fe^{III}(O$_2$$^{2-}$)]$^-$** complex, respectively. Immediately following the completion of the reaction, the final reaction mixtures

were frozen in liquid N_2. EPR sample preparation was also carried out using the same methodology.

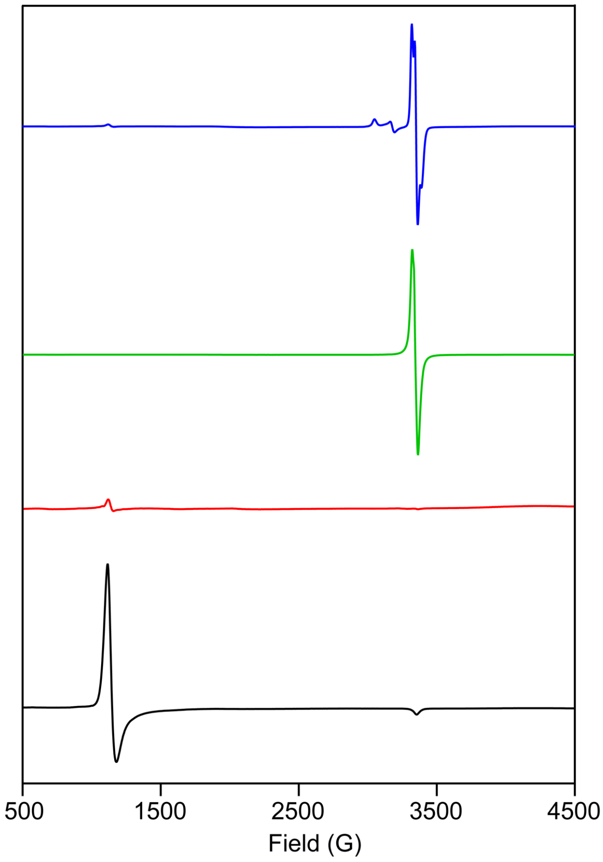

Figure S3. EPR spectral features (in frozen THF at 7 K) for 2 mM solutions of **[(F_{20}TPP)Fe^{III}($O_2^{-•}$)]** (red), compared to that of a **[(F_{20}TPP)Fe^{III}(Cl)]** (black) (g = 5.9, 2.0); TEMPO radical (green) (g = 2.0), and the final heme product from the reaction between **[(F_{20}TPP)Fe^{III}($O_2^{-•}$)]** and TEMPO–H (blue).

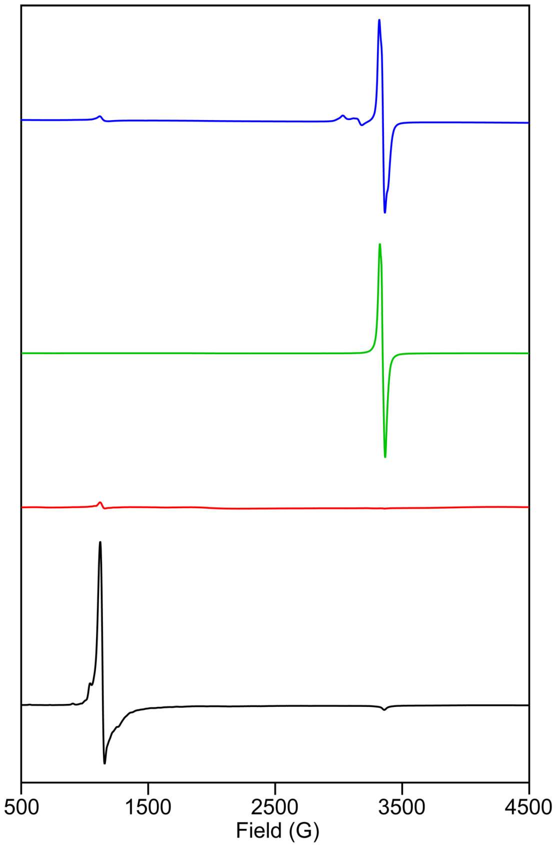

Figure S4. EPR spectral features (in frozen THF at 7 K) for 2 mM solutions of **[(TPP)Fe^{III}($O_2^{-•}$)]** (red), compared with that of a **[(TPP)Fe^{III}(Cl)]** (black) (g = 5.8, 2.0); TEMPO radical (green) (g = 2.0), and the final heme product from the reaction between **[(TPP)Fe^{III}($O_2^{-•}$)]** and TEMPO–H (blue).

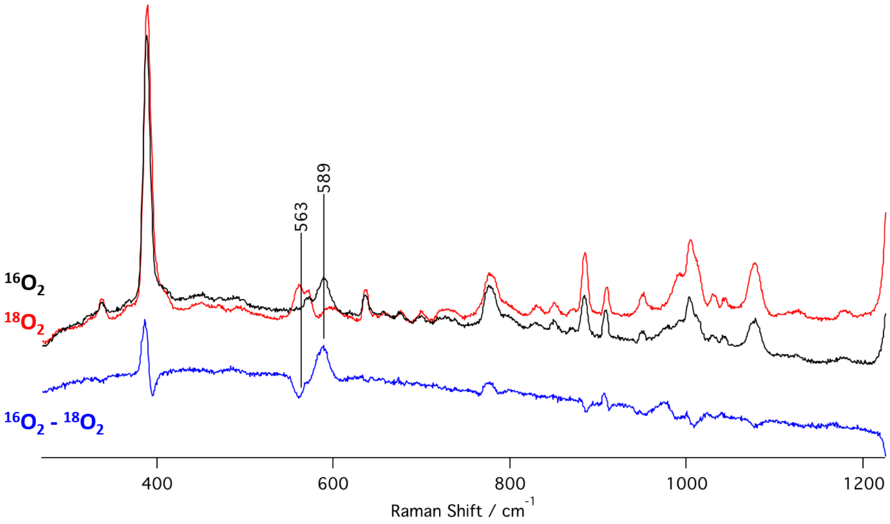

Figure S5. Resonance Raman spectra (λ_{ex} = 413.1 nm) collected from a 2 mM frozen THF solution of the final heme product from the reaction between TEMPO–H and **[(TPP)Fe^{III}($O_2^{-•}$)]** prepared with $^{16}O_{2(g)}$ (black) and $^{18}O_{2(g)}$ (red).

5. Hydrogen peroxide quantification

The formation and the yields of the **[(Por)Fe^{III}(OOH)]** complexes were further verified by H_2O_2 quantification upon its acidification.[11] In a typical experiment, to a 1 ml of a 0.5 mM THF solution of **[(Por)Fe^{III}(OOH)]** (prepared from **[(Por)Fe^{III}($O_2^{-\bullet}$)]** and TEMPO–H), 1 equiv of HCl.Et_2O (2.0 M, Sigma Aldrich) was added using a gas-tight syringe (to release H_2O_2), during which, the complete degradation of heme hydroperoxide absorption features were observed. Then, 100 μL aliquot of this solution was transferred into another cuvette with saturated NaI solution in MeCN (1 mL) at RT. This mixture was allowed to incubate for 5 min. The UV-vis spectrum of this final mixture was then recorded showing the formation of triodide (I_3^-) primarily absorbing at 362 nm (Figure S6). The yield of H_2O_2 was found to be 92% (for **[(F_{20}TPP)Fe^{III}(OOH)]**) and 88% (for **[(TPP)Fe^{III}(OOH)]**) when compared with the standard series of solutions (i.e., calibration plot; Figure S6).

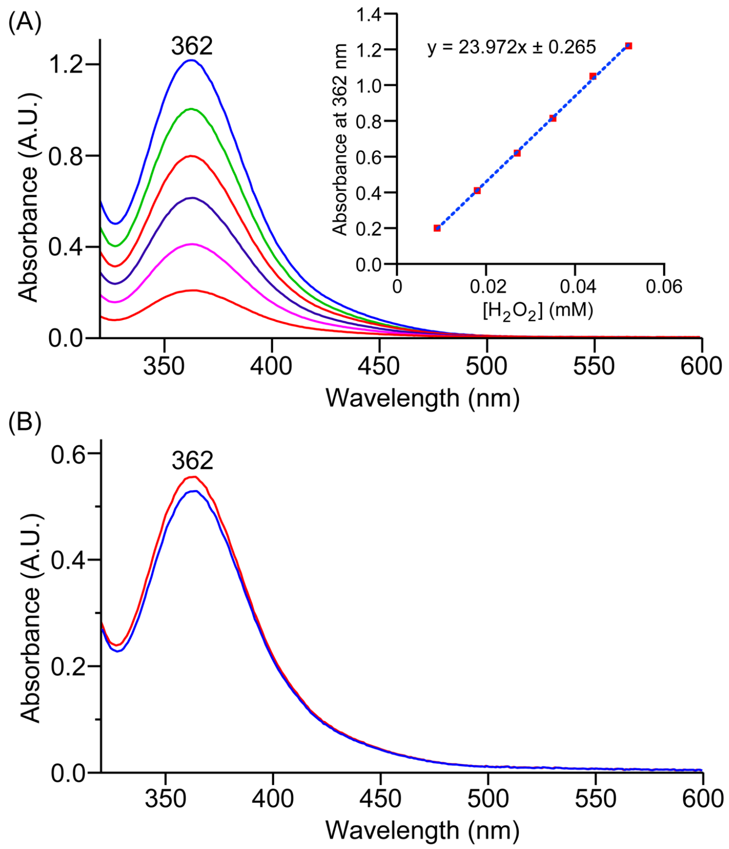

Figure S6. (A) Spectrophotometric titrations of NaI with known concentrations of H_2O_2 in acetonitrile indicating the formation of I_3^-; inset shows the calibration curve for [H_2O_2]. (B) UV–vis spectra of I_3^- generated from **[(F_{20}TPP)Fe^{III}(OOH)]** (red) and **[(TPP)Fe^{III}(OOH)]** (blue) product complexes following acidification and reactivity with NaI. *Note: electronic absorption features of heme are not observed in the final I_3^- spectra due to their very low concentrations, and impaired solubilities in MeCN.*

6. Generation of authentic heme peroxo, $[(Por)Fe^{III}(O_2^{2-})]^-$ and heme hydroperoxo $[(Por)Fe^{III}(OOH)]$ complexes (Por = porphyrinate supporting ligand)

In a typical experiment, 1 equiv of cobaltocene (in 25μL of THF) was added into a 2 mm pathlength cuvette containing a 50 μM THF solution (1 mL) of **$[(Por)Fe^{III}(O_2^{-\bullet})]$** using a Hamilton gas-tight syringe at –80 °C, and the cuvette contents were quickly mixed with dry argon bubbling to generate the **$[(Por)Fe^{III}(O_2^{2-})]^-$** species. Subsequent addition of 1 equiv of 2,6-lutidinium triflate (in 25 μL of THF) resulted in the corresponding hydroperoxo complex, **$[(Por)Fe^{III}(OOH)]$**. These complexes were characterized by UV–vis and EPR spectroscopies (Figures 4 and S7), and are in accordance with the previous literature reports.[12]

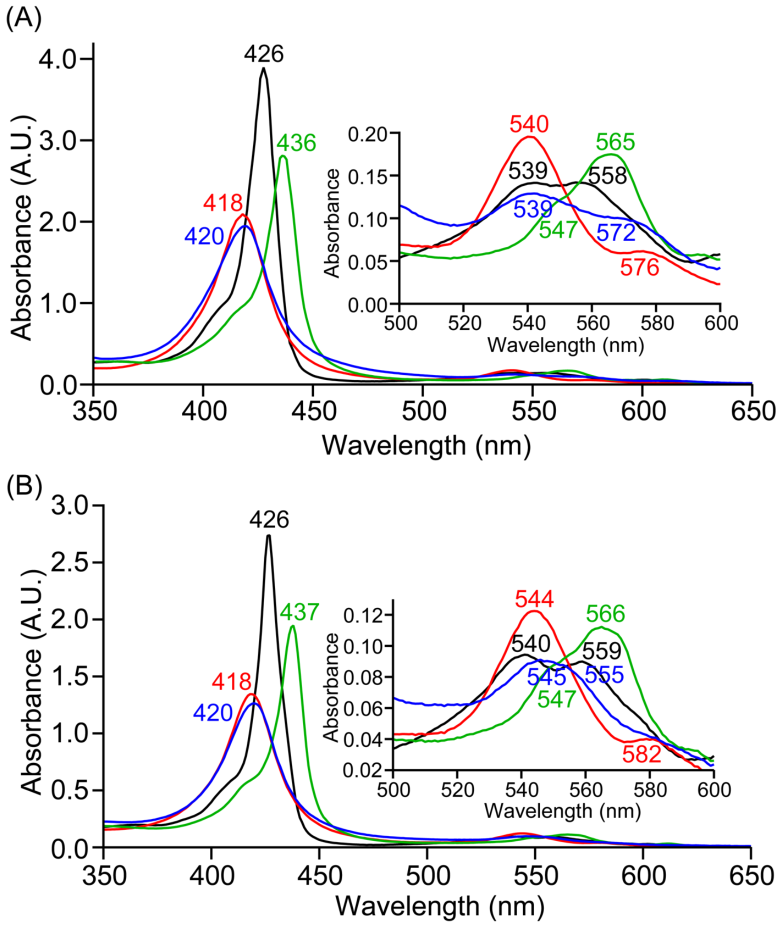

Figure S7. UV–vis spectra (in THF at −80 °C) for 50 μM solutions of: (A) **$[(THF)_2(TPP)Fe^{II}]$** (black), **$[(TPP)Fe^{III}(O_2^{-\bullet})]$** (red), **$[(TPP)Fe^{III}(O_2^{2-})]^-$** (green), **$[(TPP)Fe^{III}(OOH)]$** (blue); (B) **$[(THF)_2(TMP)Fe^{II}]$** (black), **$[(TMP)Fe^{III}(O_2^{-\bullet})]$** (red), **$[(TMP)Fe^{III}(O_2^{2-})]^-$** (green), **$[(TMP)Fe^{III}(OOH)]$** (blue).

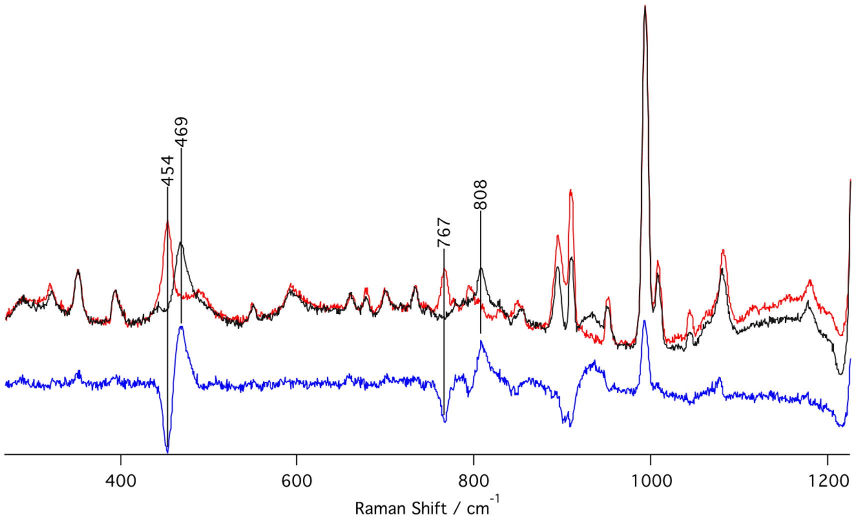

Figure S8. Resonance Raman spectra (λ_{ex} = 413.1 nm) collected from a 2 mM frozen THF solution of **[(F$_{20}$TPP)Fe^{III}(O$_2$$^{2-}$)]$^-$** prepared with $^{16}O_{2(g)}$ (black) and $^{18}O_{2(g)}$ (red).

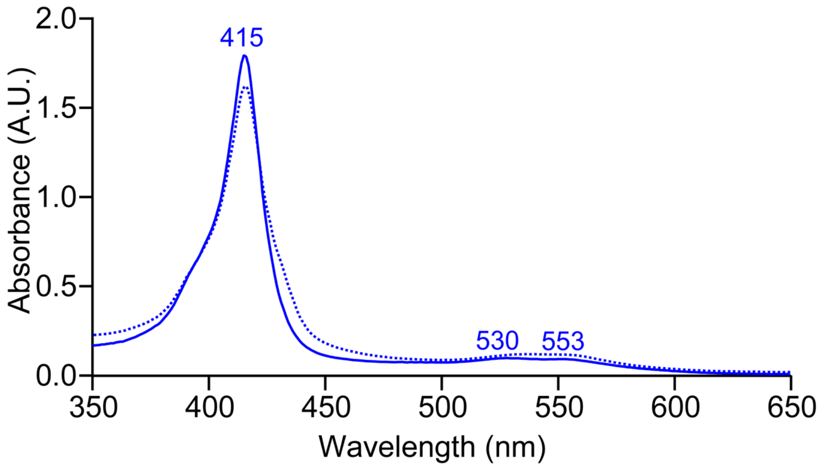

Figure S9. UV−vis spectral comparison (in THF at −80 °C) of the final heme product from the reaction of **[(F$_{20}$TPP)Fe^{III}(O$_2$$^{-•}$)]** with 100 equiv of TEMPO–H (blue solid) and authentically generated **[(F$_{20}$TPP)Fe^{III}(OOH)]** complex (blue dotted).

Table S1. Spectroscopic characterization details for **[(F$_{20}$TPP)Fe^{III}(O$_2$$^{-•}$)]**, **[(F$_{20}$TPP)Fe^{III}(O$_2$$^{2-}$)]$^-$**, and **[(F$_{20}$TPP)Fe^{III}(OOH)]** species.

Identity of the heme complex	UV-vis (nm) (in THF at −80 °C)	^{2}H NMR (ppm) (in THF at −80 °C)	EPR (in frozen THF at 7K)	Resonance Raman (cm^{-1}) (in frozen THF)
**[(F$_{20}$TPP)Fe^{III}(O$_2$$^{-•}$)]**	413 (Soret), 532, 560	9.1	Silent	ν(Fe–O) 577<br>ν(O–O) 1137
**[(F$_{20}$TPP)Fe^{III}(O$_2$$^{2-}$)]$^-$**	432 (Soret), 557	NR[a]	4.21	ν(Fe–O) 469<br>ν(O–O) 808
**[(F$_{20}$TPP)Fe^{III}(OOH)]**	415 (Soret), 530, 553	−1.2[b]	2.26, 2.15, 1.96	ν(Fe–O) 597[b]

[a]Not reported/observed; [b]Measured for PCET reaction product from **[(F$_{20}$TPP)Fe^{III}(O$_2$$^{-•}$)]** + TEMPOH.

7. Spectroscopic reactivity and kinetic studies of **[(Por)Fe^{III}($O_2^{-\bullet}$)]** complexes with TEMPO–H(D)

For each kinetic experiment, 50 μM THF solutions of **[(Por)Fe^{III}($O_2^{-\bullet}$)]** complexes (1 mL) were generated in a 2 mm pathlength Schlenk cuvette as previously described (*vide supra*). Subsequently, 100 equiv of TEMPO–H (50 μL in THF) was added into the cuvette using a gas-tight syringe, and the reaction mixture was quickly mixed with dry argon bubbling. The reaction was monitored by the progression of Soret band spectral changes centered at 415 nm (for **[(F_{20}TPP)Fe^{III}($O_2^{-\bullet}$)]**) and 418 nm (for **[(TPP)Fe^{III}($O_2^{-\bullet}$)]**) until plateaued. Kinetic studies were carried out, under pseudo-first-order conditions, by the addition of 50–200 equiv of TEMPO–H (or TEMPO–D) to a 50 μM 1 mL THF solution of **[(Por)Fe^{III}($O_2^{-\bullet}$)]** at –80 °C. Kinetic experiments at variable temperatures (at –70, –80, –90, and –100 °C) were performed following the same procedure as described, allowing the cuvette to achieve thermal equilibrium (10 min) in the cryostat, prior to the addition of the substrate. The pseudo-first-order rate constants, k_{obs} were calculated from plots of ln[(A–A_f)/(A_i–A_f)] *vs* time(s), where A_i and A_f are initial and final absorbencies, respectively (Figure S10). The second-order rate constants (k_2) were obtained from the slope of the best-fit line from a plot of k_{obs} values *vs* substrate concentration (Figures 5 and S11).

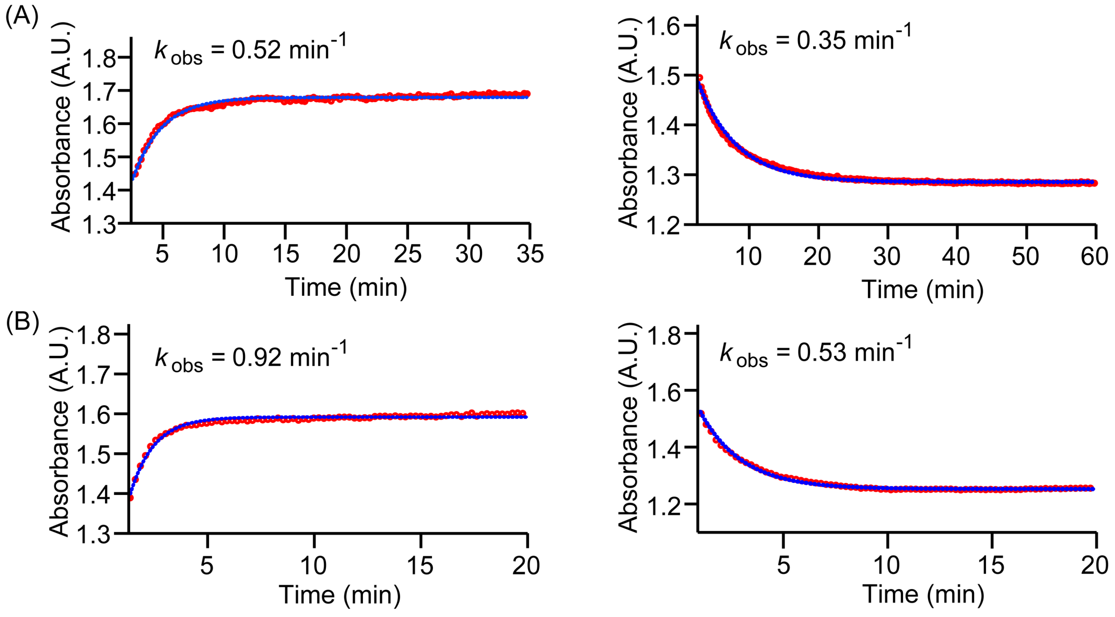

Figure S10. Plots of the change in absorbance versus time (red) with best-fit lines (blue) for the reaction of 50 μM solution of **[(F_{20}TPP)Fe^{III}($O_2^{-\bullet}$)]** (415 nm) (*left*) and **[(TPP)Fe^{III}($O_2^{-\bullet}$)]** (418 nm) (*right*) with (A) 100 and (B) 150 equiv of TEMPO–H in THF at –80 °C.

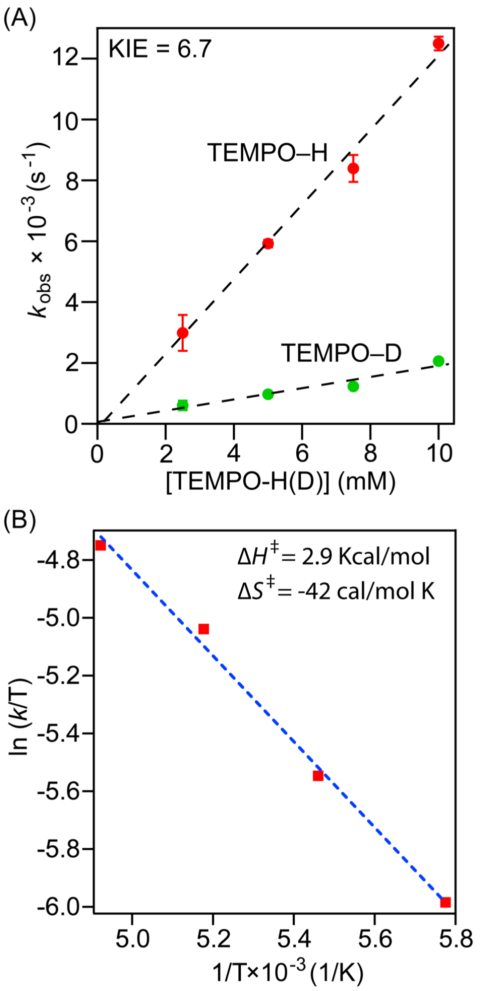

Figure S11. (A) Plot of pseudo-first-order rate constants (k_{obs}) versus [TEMPO–H] (red) or [TEMPO–D] (green) with best-fit lines (black) for a 50 μM solution of **[(TPP)Fe^{III}(O$_2^{-•}$)]**. (B) Eyring plot showing ln(k/T) versus 1/T for the reaction of 50 μM solution of **[(TPP)Fe^{III}(O$_2^{-•}$)]** with TEMPO–H at −70, –80, –90 and –100 °C.

8. Determination of reduction potentials of [(Por)Fe^{III}($O_2^{-•}$)] complexes

[(Por)Fe^{III}($O_2^{-•}$)] complexes were generated as described above. These **[(Por)Fe^{III}($O_2^{-•}$)]** complexes were titrated with Cr(η^6-C_6H_6)$_2$ in THF at –80 °C. After each addition of Cr(η^6-C_6H_6)$_2$, an equilibrated mixture was established (2 min). For each equilibrium mixture, the concentration of each species in solution was computed taking into account the absorption features at 432, 436 and 437 nm for **[(F_{20}TPP)Fe^{III}($O_2^{-•}$)]**, **[(TPP)Fe^{III}($O_2^{-•}$)]**, and **[(TMP)Fe^{III}($O_2^{-•}$)]**, respectively, and the solution mass balance (Table S1). From these equilibrium constants, corresponding reduction potentials were calculated using the Nernst equation (eq. S1-S4 and Table S2-S3).

Table S2. Equilibrium concentrations obtained during the titration of **[(Por)Fe^{III}($O_2^{-•}$)]** with Cr(η^6-C_6H_6)$_2$ in THF at –80 °C.

For [(F_{20}TPP)Fe^{III}($O_2^{-•}$)]				
[Cr(η^6-C_6H_6)$_2$]$_{added}$	[Cr(η^6-C_6H_6)$_2$]$_{eq}$	[(Por)Fe^{III}(O_2^{2-})]$^-_{eq}$	[(Por)Fe^{III}($O_2^{-•}$)]$_{eq}$	[Cr(η^6-C_6H_6)$_2$]$^+_{eq}$
1.69 x 10^{-5}	0.23 x 10^{-5}	1.46 x 10^{-5}	3.66 x 10^{-5}	1.46 x 10^{-5}
3.38 x 10^{-5}	1.17 x 10^{-5}	2.21 x 10^{-5}	2.91 x 10^{-5}	2.21 x 10^{-5}
5.06 x 10^{-5}	1.91 x 10^{-5}	3.15 x 10^{-5}	1.97 x 10^{-5}	3.15 x 10^{-5}
6.76 x 10^{-5}	2.83 x 10^{-5}	3.93 x 10^{-5}	1.19 x 10^{-5}	3.93 x 10^{-5}
8.45 x 10^{-5}	3.78 x 10^{-5}	4.67 x 10^{-5}	0.45 x 10^{-5}	4.67 x 10^{-5}
**For [(TPP)Fe^{III}($O_2^{-•}$)]**				
2.51 x 10^{-5}	0.33 x 10^{-5}	2.18 x 10^{-5}	2.82 x 10^{-6}	2.18 x 10^{-5}
5.01 x 10^{-5}	1.88 x 10^{-5}	3.13 x 10^{-5}	1.87 x 10^{-5}	3.13 x 10^{-5}
7.52 x 10^{-5}	3.46 x 10^{-5}	4.06 x 10^{-5}	0.94 x 10^{-5}	4.06 x 10^{-5}
10.05 x 10^{-5}	5.55 x 10^{-5}	4.50 x 10^{-5}	0.50 x 10^{-5}	4.50 x 10^{-5}
12.52 x 10^{-5}	7.66 x 10^{-5}	4.86 x 10^{-5}	0.14 x 10^{-5}	4.86 x 10^{-5}
**For [(TMP)Fe^{III}($O_2^{-•}$)]**				
2.98 x 10^{-5}	0.04 x 10^{-5}	2.94 x 10^{-5}	3.66 x 10^{-5}	2.94 x 10^{-5}
5.01 x 10^{-5}	0.31 x 10^{-5}	4.70 x 10^{-5}	1.90 x 10^{-5}	4.70 x 10^{-5}
7.48 x 10^{-5}	1.95 x 10^{-5}	5.53 x 10^{-5}	1.07 x 10^{-5}	5.53 x 10^{-5}
10.02 x 10^{-5}	3.77 x 10^{-5}	6.25 x 10^{-5}	0.35 x 10^{-5}	6.25 x 10^{-5}
14.95 x 10^{-5}	8.47 x 10^{-5}	6.48 x 10^{-5}	0.12 x 10^{-5}	6.48 x 10^{-5}

$E = E°' – (RT/nF)\ln(Q)$ (S1)

$0 = E°' – (RT/nF)\ln(K_{eq})$ (S2)

$E°' = E_S – E_{Cr}$ (S3)

$E_S = E_{Cr} + (RT/nF)\ln(K_{eq})$ (S4)

E is the potential of the system, which is zero at equilibrium. R is the universal gas constant (8.314 J/Kmol), T is the temperature of the system (193.15 K), n is the number of electrons (1), F is the Faraday's constant (96485.33 C/mol), E_{Cr} is the reduction potential of $Cr(\eta^6\text{-}C_6H_6)_2$ (–1.15 V vs. $Fc^{+/0}$ in CH_2Cl_2), K_{eq} is the equilibrium constant (values given in Table S2), and E_S is the reduction potential of the **[(Por)Fe^{III}(O$_2^{-•}$)]** complexes.

Table S3. Calculated E_s values corresponding to the reduction potential of **[(Por)Fe^{III}(O$_2^{-•}$)]** complexes in THF at –80 °C.

**For [(F$_{20}$TPP)Fe^{III}(O$_2^{-•}$)]**	
K_{eq}	E_s **(mV)**
0.395	–1.165
0.697	–1.156
0.379	–1.166
0.218	–1.175
0.078	–1.192
**For [(TPP)Fe^{III}(O$_2^{-•}$)]**	
0.195	–1.177
0.358	–1.167
0.197	–1.177
0.137	–1.184
0.045	–1.201
**For [(TMP)Fe^{III}(O$_2^{-•}$)]**	
0.016	–1.218
0.026	–1.210
0.068	–1.194
0.033	–1.206
0.024	–1.212

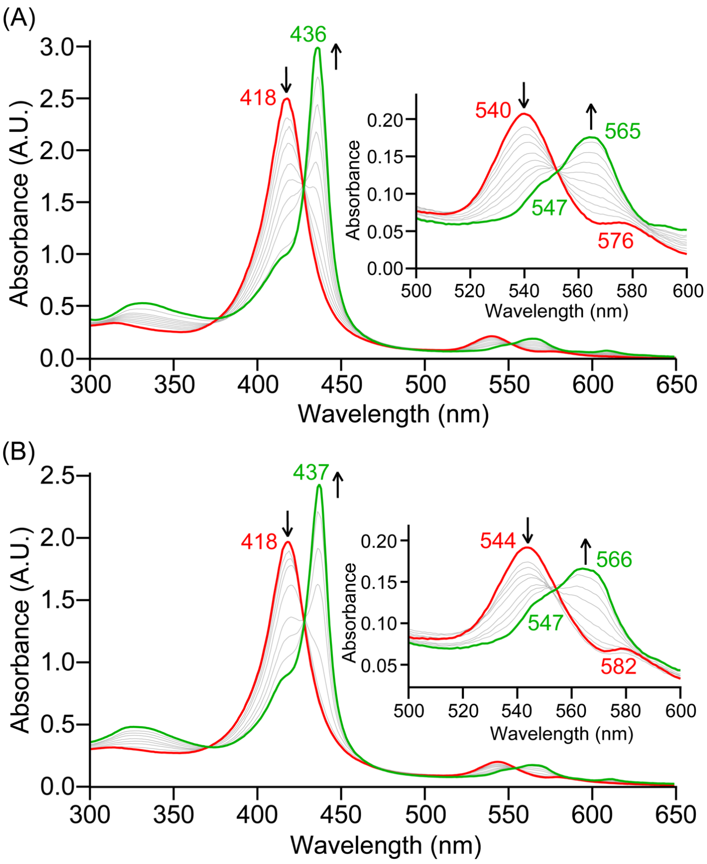

Figure S12. UV−vis spectral changes (in THF at –80 °C) during the conversion of (A) **[(TPP)Fe^{III}(O$_2^{-•}$)]** (red) to **[(TPP)Fe^{III}(O$_2^{2-}$)]$^-$** (green), and (B) **[(TMP)Fe^{III}(O$_2^{-•}$)]** (red) to **[(TMP)Fe^{III}(O$_2^{2-}$)]$^-$** (green) upon incremental addition of Cr(η^6-C$_6$H$_6$)$_2$.

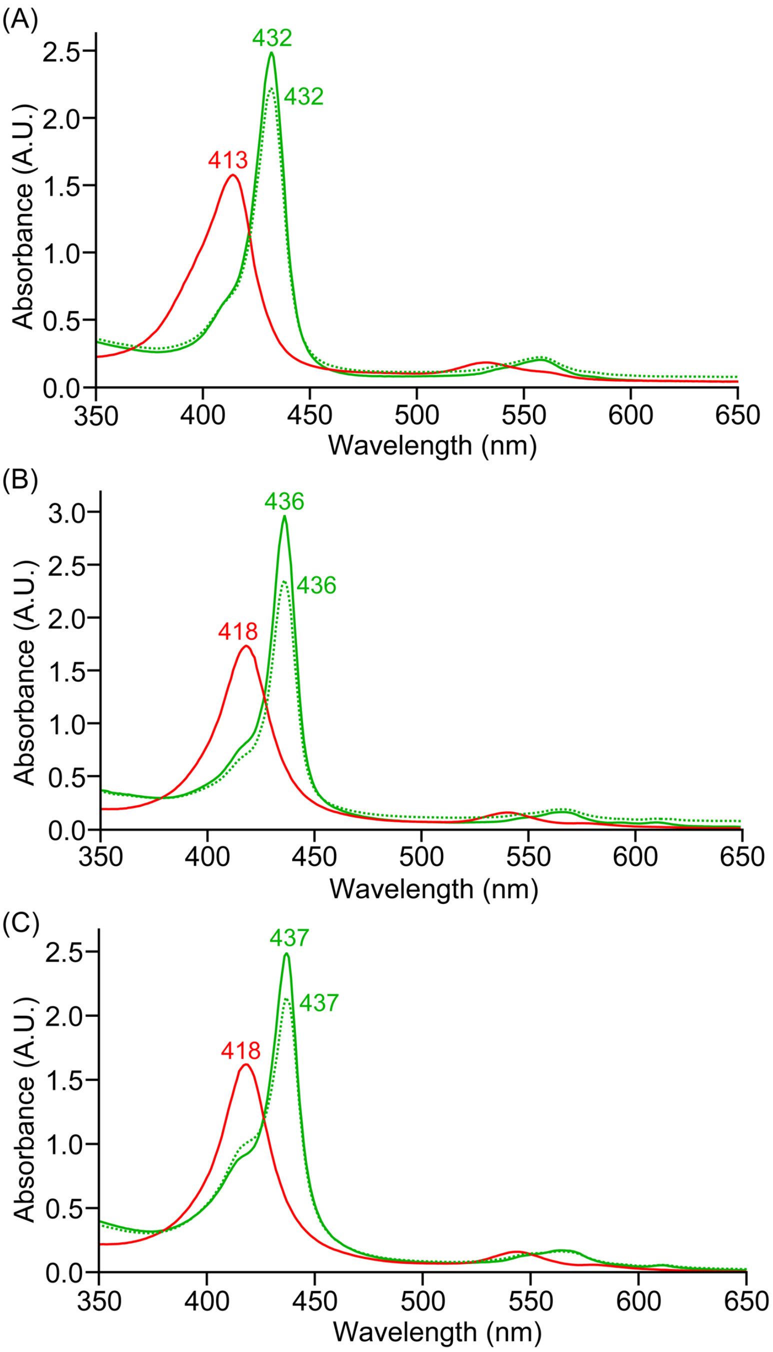

Figure S13. UV−vis spectral changes (in THF at –80 °C) observed during the reversible interconversion of **[(Por)Fe^{III}(O_2^{2-})]$^-$** (green) to **[(Por)Fe^{III}($O_2^{-\bullet}$)]** (red) using ([(4-BrC$_6$H$_4$)$_3$N]SbCl$_6$) and **[(Por)Fe^{III}($O_2^{-\bullet}$)]** (red) back to **[(Por)Fe^{III}(O_2^{2-})]$^-$** (dotted green) using Cr(η^6-C$_6$H$_6$)$_2$ for (A) **[(F$_{20}$TPP)Fe^{III}(O_2^{2-})]$^-$**, (B) **[(TPP)Fe^{III}(O_2^{2-})]$^-$** and (C) **[(TMP)Fe^{III}(O_2^{2-})]$^-$** adducts.

9. Determination of pK_a values of [(Por)Fe^{III}(OOH)] complexes

In a Schlenk cuvette, **[(Por)Fe^{III}(OOH)]** complexes were generated as described above, and titrated by the ^{t}BuP$_2$(dma) base in THF at –80 °C. After each addition of ^{t}BuP$_2$(dma), an equilibrated mixture was established (2 min). For each equilibrium mixture, the concentration of each species in solution was computed taking into account the absorption at 432, 436 and 437 nm for **[(F_{20}TPP)Fe^{III}(OOH)]**, **[(TPP)Fe^{III}(OOH)]**, and **[(TMP)Fe^{III}(OOH)]**, respectively, and the solution mass balance (Table S4). From these equilibrium constants, the pK_a values were calculated (eq. S5 and Table S5).

Table S4. Equilibrium concentrations obtained during the titration of **[(Por)Fe^{III}(OOH)]** with ^{t}BuP$_2$(dma) in THF at –80 °C.

**For [(F_{20}TPP)Fe^{III}(OOH)]**				
**[^{t}BuP$_2$(dma)]$_{added}$**	**[(Por)Fe^{III}(O_2^{2-})]$_{eq}$**	**[^{t}BuP$_2$(dma)]$_{eq}$**	**[(Por)Fe^{III}(OOH)]$_{eq}$**	**[^{t}BuP$_2$(dma)H^+]$_{eq}$**
2.01 x 10^{-5}	1.83 x 10^{-6}	0.18 x 10^{-6}	3.17 x 10^{-6}	1.83 x 10^{-6}
3.96 x 10^{-5}	2.65 x 10^{-5}	1.31 x 10^{-5}	2.35 x 10^{-5}	2.65 x 10^{-5}
6.03 x 10^{-5}	3.33 x 10^{-5}	2.70 x 10^{-5}	1.67 x 10^{-5}	3.33 x 10^{-5}
7.95 x 10^{-5}	3.86 x 10^{-5}	4.09 x 10^{-5}	1.14 x 10^{-5}	3.86 x 10^{-5}
10.12 x 10^{-5}	4.84 x 10^{-5}	5.28 x 10^{-5}	0.16 x 10^{-5}	4.84 x 10^{-5}
**For [(TPP)Fe^{III}(OOH)]**				
3.72 x 10^{-5}	2.10 x 10^{-6}	1.62 x 10^{-6}	4.10 x 10^{-6}	2.10 x 10^{-6}
7.44 x 10^{-5}	2.71 x 10^{-5}	4.73 x 10^{-5}	3.49 x 10^{-5}	2.71 x 10^{-5}
11.16 x 10^{-5}	3.28 x 10^{-5}	7.88 x 10^{-5}	2.92 x 10^{-5}	3.28 x 10^{-5}
14.88 x 10^{-5}	3.78 x 10^{-5}	11.10 x 10^{-5}	2.42 x 10^{-5}	3.78 x 10^{-5}
18.60 x 10^{-5}	4.28 x 10^{-5}	14.32 x 10^{-5}	1.92 x 10^{-5}	4.28 x 10^{-5}
**For [(TMP)Fe^{III}(OOH)]**				
6.51 x 10^{-5}	1.96 x 10^{-6}	4.55 x 10^{-6}	4.14 x 10^{-6}	1.96 x 10^{-6}
12.25 x 10^{-5}	2.50 x 10^{-5}	9.75 x 10^{-5}	3.60 x 10^{-5}	2.50 x 10^{-5}
16.50 x 10^{-5}	2.96 x 10^{-5}	13.54 x 10^{-5}	3.14 x 10^{-5}	2.96 x 10^{-5}
20.55 x 10^{-5}	3.55 x 10^{-5}	17.05 x 10^{-5}	2.55 x 10^{-5}	3.55 x 10^{-5}
25.10 x 10^{-5}	3.95 x 10^{-5}	21.15 x 10^{-5}	2.00 x 10^{-5}	3.95 x 10^{-5}

pK_a^{THF}**[(Por)Fe^{III}(OOH)]** = pK_a^{THF}(^{t}BuP$_2$(dma)) – pK_{eq} (S5)

Table S5. Calculated pK_a values of **[(Por)Fe^{III}(OOH)]** in THF at –80 °C.

**For [(F_{20}TPP)Fe^{III}(OOH)]**		
K_{eq}	pK_{eq}	pK_a
5.86	-0.76	25.66
2.28	-0.35	25.25
2.45	-0.39	25.29
3.19	-0.50	25.40
27.72	-1.44	26.34
**For [(TPP)Fe^{III}(OOH)]**		
0.66	0.17	24.73
0.44	0.35	24.55
0.46	0.33	24.57
0.53	0.27	24.63
0.66	0.18	24.72
**For [(TMP)Fe^{III}(OOH)]**		
0.20	0.69	24.20
0.17	0.75	24.15
0.20	0.69	24.20
0.29	0.53	24.36
0.36	0.43	24.47

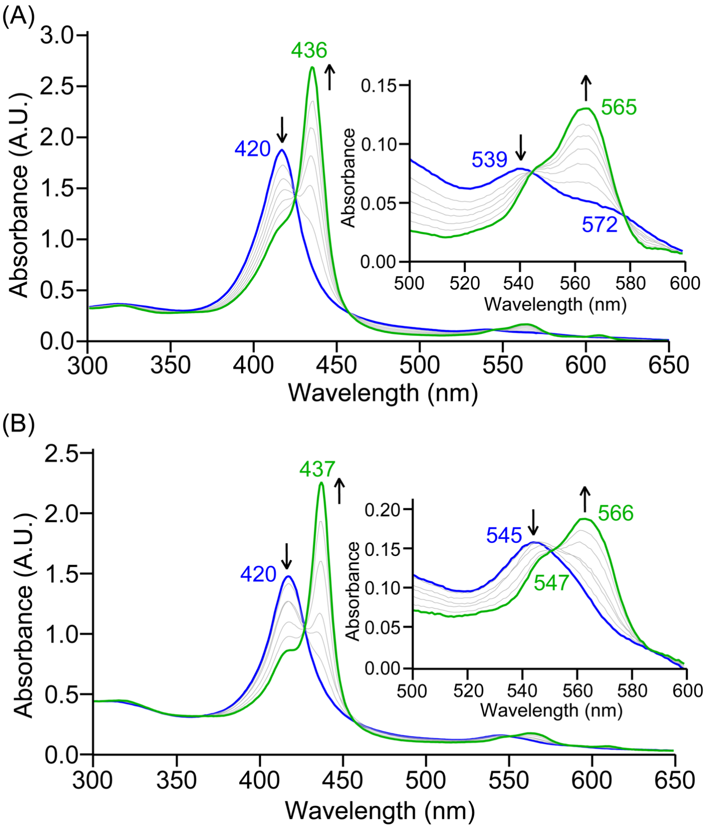

Figure S14. UV–vis spectral changes (in THF at –80 °C) during the conversion of (A) **[(TPP)Fe^{III}(OOH)]** (blue) to **[(TPP)Fe^{III}(O_2^{2-})]$^-$** (green), and (B) **[(TMP)Fe^{III}(OOH)]** (blue) to **[(TMP)Fe^{III}(O_2^{2-})]$^-$** (green) upon incremental addition of ^{t}BuP$_2$(dma).

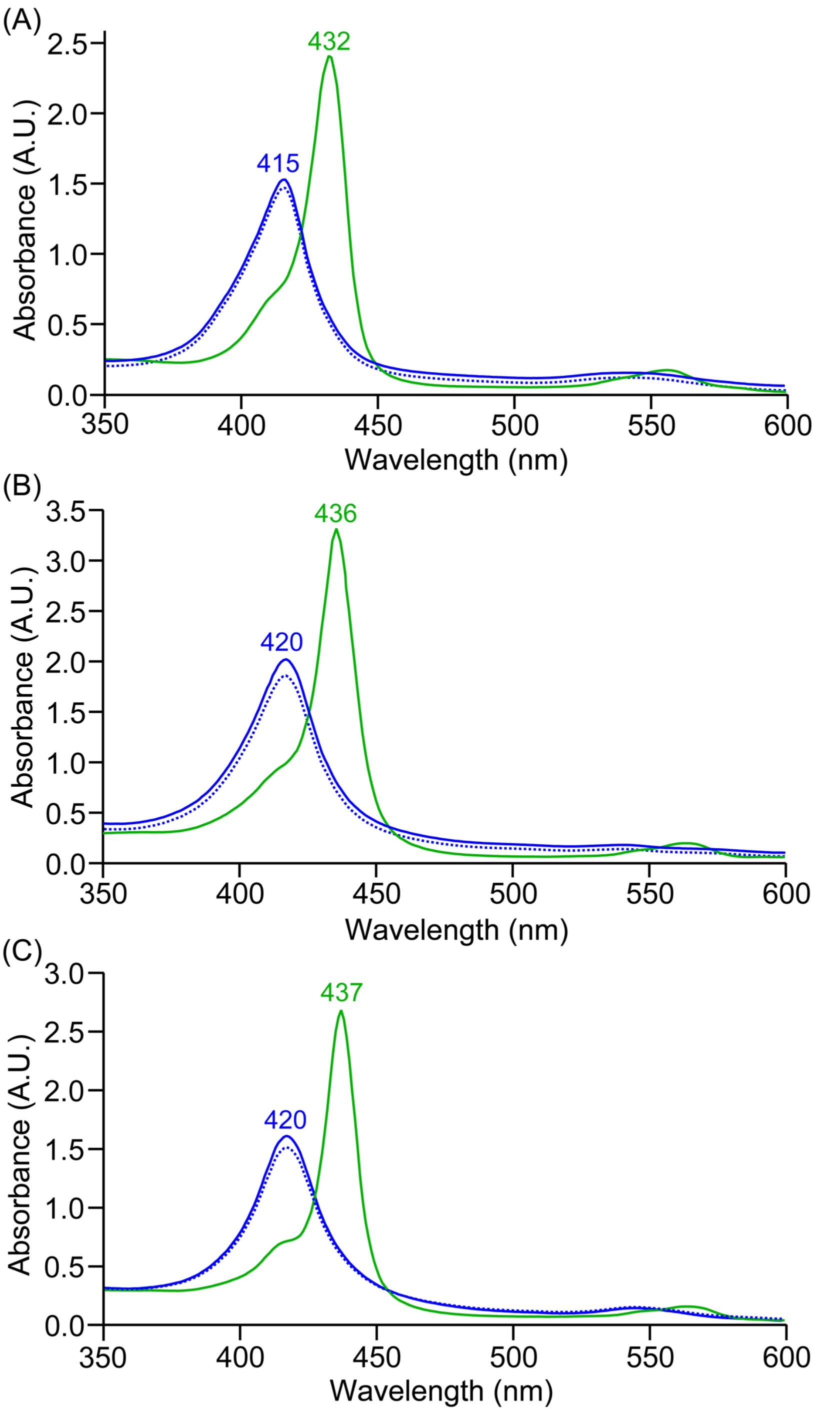

Figure S15. UV−vis spectral changes (in THF at –80 °C) during the reversible interconversion of **[(Por)Fe^{III}(OOH)]** (blue) to **[(Por)Fe^{III}(O_2^{2-})]$^-$** (green) using ^{t}BuP$_2$(dma) and **[(Por)Fe^{III}(O_2^{2-})]$^-$** (green) back to **[(Por)Fe^{III}(OOH)]** (blue dotted) using [LuH]OTf for (A) **[(F_{20}TPP)Fe^{III}(OOH)]**, (B) **[(TPP)Fe^{III}(OOH)]** and (C) **[(TMP)Fe^{III}(OOH)]** complexes.

10. Tables for computational calculations

Table S6. Absolute energies, zero-point and free energies (in a.u.) of optimized geometries for the reaction of **[(TPP)Fe^{III}(O$_2^{-\bullet}$)]** with the substrate TEMPO-H as obtained in Gaussian-09. All calculations were done with a continuum polarized conductor model (CPCM) included with the solvent THF that has a dielectric constant of 7.6.

Singlet spin	E [au]	ZPE [au]	G [au]	E [au]
	B3LYP/BS1	B3LYP/BS1	B3LYP/BS1	B3LYP/BS2
^{1}**[(TPP)Fe^{III}(O$_2^{-\bullet}$)]**	-2418.328453	0.729316	-2417.641474	-2414.198958
TEMPO-H	-484.19675	0.27530	-483.94156	-484.43818
^{1}**Re**	-2902.536261	1.005956	-2901.580815	-2898.634149
^{1}**TS**	-2902.531109	1.001856	-2901.578455	-2898.624917
^{1}**Pr**	-2902.561158	1.006969	-2901.604102	-2898.649902

Triplet spin	E [au]	ZPE [au]	G [au]	E [au]
	B3LYP/BS1	B3LYP/BS1	B3LYP/BS1	B3LYP/BS2
^{3}**[(TPP)Fe^{III}(O$_2^{-\bullet}$)]**	-2418.32536	0.72945	-2417.63880	-2414.18774
TEMPO-H	-484.19675	0.27530	-483.94156	-484.43818
^{3}**Re**	-2902.53468	1.00584	-2901.58012	-2898.62517
^{3}**TS**	-2902.52763	1.00227	-2901.57547	-2898.61476
^{3}**Pr**	-2902.55491	1.00628	-2901.60066	-2898.64556

Quintet spin	E [au]	ZPE [au]	G [au]	E [au]
	B3LYP/BS1	B3LYP/BS1	B3LYP/BS1	B3LYP/BS2
^{5}**[(TPP)Fe^{III}(O$_2^{-\bullet}$)]**	-2418.268370	0.72651	-2417.584590	-2414.123980
TEMPO-H	-484.19675	0.27530	-483.94156	-484.43818
^{5}**Re**	-2902.477912	1.00307	-2901.525231	-2898.565379
^{5}**TS**	-2902.470451	0.99914	-2901.521938	-2898.593732
^{5}**Pr**	-2902.497940	1.00301	-2901.547670	-2898.587680

Table S7. Relative energies, zero-point energies and free energies (in kcal mol^{-1}) of optimized geometries for the reaction of **[(TPP)Fe^{III}(O$_2^{-\bullet}$)]** with TEMPO-H as obtained in Gaussian-09.

	ΔE	ΔE+ZPE	ΔG	ΔE	ΔE+ZPE	ΔG
	BS1	BS1	BS1	BS2	BS2	BS2
**Singlet-Re**	0.00	0.00	0.00	0.00	0.00	0.00
**Singlet-TS**	3.23	0.66	1.48	5.79	3.22	4.04
**Singlet-Pr**	-15.62	-14.99	-14.61	-9.89	-9.25	-8.87
**Triplet-Re**	0.99	0.92	0.44	5.64	5.57	5.08
**Triplet-TS**	5.42	3.11	3.35	12.17	9.86	10.10
**Triplet-Pr**	-11.70	-11.50	-12.46	-7.16	-6.96	-7.91
**Quintet-Re**	36.61	34.80	34.88	43.15	41.34	41.42
**Quintet-TS**	41.30	37.02	36.95	25.36	21.09	21.01
**Quintet-Pr**	24.05	22.20	20.80	29.16	27.31	25.91

Table S8. Absolute energies, zero-point and free energies (in a.u.) of optimized geometries for the reaction of **[(F$_{20}$TPP)Fe^{III}(O$_2^{-\bullet}$)]** with the substrate TEMPO-H as obtained in Gaussian-09. All calculations were done with a continuum polarized conductor model (CPCM) included with the solvent THF that has a dielectric constant of 7.6.

Singlet spin	E [au]	ZPE [au]	G [au]	E [au]
	B3LYP/BS1	B3LYP/BS1	B3LYP/BS1	B3LYP/BS2
^{1}**[(F$_{20}$TPP)Fe^{III}(O$_2^{-\bullet}$)]**	-4402.42833	0.55998	-4401.92155	-4399.44131
TEMPO-H	-484.19675	0.27530	-483.94156	-484.43818
^{1}**Re**	-4886.635761	0.836622	-4885.860309	-4883.877039
^{1}**TS**	-4886.631196	0.833070	-4885.858197	-4883.867496
^{1}**Pr**	-4886.662420	0.837149	-4885.886580	-4883.891011

Table S9. Relative energies, zero-point energies and free energies (in kcal mol^{-1}) of optimized geometries for the reaction of **[(F$_{20}$TPP)Fe^{III}(O$_2^{-\bullet}$)]** with TEMPO-H as obtained in Gaussian-09.

	ΔE	ΔE+ZPE	ΔG	ΔE	ΔE+ZPE	ΔG
	BS1	BS1	BS1	BS2	BS2	BS2
^{1}**Re**	0.00	0.00	0.00	0.00	0.00	0.00
^{1}**TS**	2.86	0.64	1.33	5.99	3.76	4.45
^{1}**Pr**	-16.73	-16.40	-16.49	-8.77	-8.44	-8.52

Table S10. Group spin densities of UB3LYP/BS1+solvent optimized geometries for the reaction pathway from **[(TPP)Fe^{III}(O$_2^{-\bullet}$)]** with TEMPO-H **^{1}Re** as obtained in Gaussian-09.

	ρ_{Fe}	ρ_{ligand}	ρ_H	ρ_{TEMPO}
^{1}**Re**	-1.12	1.10	-0.01	0.02
^{1}**TS**	1.10	-0.86	0.03	-0.28
^{1}**Pr**	0.75	-1.74	0.00	0.98

Table S11. Group spin densities of UB3LYP/BS1+solvent optimized geometries for the reaction pathway from **[(TPP)Fe^{III}(O$_2^{-\bullet}$)]** with TEMPO-H **^{3}Re** as obtained in Gaussian-09.

	ρ_{Fe}	ρ_{ligand}	ρ_H	ρ_{TEMPO}
^{3}**Re**	1.06	0.93	0.00	0.01
^{3}**TS**	0.93	0.30	-0.03	0.79
^{3}**Pr**	0.98	0.03	0.00	0.99

Table S12. Group spin densities of UB3LYP/BS1+solvent optimized geometries for the reaction pathway from **[(TPP)Fe^{III}(O$_2^{-\bullet}$)]** with TEMPO-H **^{5}Re** as obtained in Gaussian-09.

	ρ_{Fe}	ρ_{ligand}	ρ_H	ρ_{TEMPO}
^{5}**Re**	1.02	2.96	-0.01	0.02
^{5}**TS**	0.98	2.77	-0.03	0.28
^{5}**Pr**	0.97	2.05	0.00	0.99

Table S13. Group spin densities of UB3LYP/BS1+solvent optimized geometries for the reaction pathway from **[(F$_{20}$TPP)Fe^{III}(O$_2^{-\bullet}$)]** with TEMPO-H **^{1}Re** as obtained in Gaussian-09.

	ρ_{Fe}	ρ_{ligand}	ρ_H	ρ_{TEMPO}
^{1}**Re**	1.11	-1.10	0.01	-0.02
^{1}**TS**	1.09	-0.89	0.03	-0.22
^{1}**Pr**	0.97	0.02	0.00	-0.98

Table S14. Group charges of UB3LYP/BS1+solvent and UB3LYP/BS2+solvent optimized geometries for the reaction pathway from **[(TPP)Fe^{III}($O_2^{-•}$)]** with TEMPO-H ^{1}**Re** as obtained in Gaussian-09.

UB3LYP/BS1 + sol	Fe	ligand	H	TEMPO	Total
^{1}**Re**	0.80	-1.78	0.41	0.58	0.00
^{1}**TS**	0.78	-1.91	0.43	0.70	0.00
^{1}**Pr**	0.69	-2.17	0.42	1.05	0.00

UB3LYP/BS2 + sol	Fe	ligand	H	TEMPO	Total
^{1}**Re**	1.22	-2.86	0.15	1.49	0.00
^{1}**TS**	1.42	-3.43	0.39	1.63	0.00
^{1}**Pr**	0.96	-2.97	0.29	1.72	0.00

Table S15. Group charges of UB3LYP/BS1+solvent and UB3LYP/BS2+solvent optimized geometries for the reaction pathway from **[(TPP)Fe^{III}($O_2^{-•}$)]** with TEMPO-H ^{3}**Re** as obtained in Gaussian-09.

UB3LYP/BS1 + sol	Fe	ligand	H	TEMPO	Total
^{3}**Re**	0.80	-1.80	0.38	0.62	0.00
^{3}**TS**	0.74	-2.20	0.45	1.01	0.00
^{3}**Pr**	0.74	-2.23	0.43	1.06	0.00

UB3LYP/BS2 + sol	Fe	ligand	H	TEMPO	Total
^{3}**Re**	1.24	-2.87	0.15	1.48	0.00
^{3}**TS**	1.24	-3.19	0.33	1.61	0.00
^{3}**Pr**	1.10	-3.12	0.30	1.72	0.00

Table S16. Group charges of UB3LYP/BS1+solvent and UB3LYP/BS2+solvent optimized geometries for the reaction pathway from **[(TPP)Fe^{III}($O_2^{-•}$)]** with TEMPO-H ^{5}**Re** as obtained in Gaussian-09.

UB3LYP/BS1 + sol	Fe	ligand	H	TEMPO	Total
^{5}**Re**	0.79	-1.79	0.41	0.59	0.00
^{5}**TS**	0.78	-1.92	0.44	0.71	0.00
^{5}**Pr**	0.74	-2.24	0.43	1.07	0.00

UB3LYP/BS2 + sol	Fe	ligand	H	TEMPO	Total
^{5}**Re**	0.98	-2.62	0.16	1.48	0.00
^{5}**TS**	1.34	-3.12	0.36	1.43	0.00
^{5}**Pr**	0.81	-2.80	0.31	1.68	0.00

Table S17. Group charges of UB3LYP/BS1+solvent and UB3LYP/BS2+solvent optimized geometries for the reaction pathway from **[(F_{20}TPP)Fe^{III}($O_2^{-\bullet}$)]** with TEMPO-H ^{1}**Re** as obtained in Gaussian-09.

UB3LYP/BS1 + sol	Fe	ligand	H	TEMPO	Total
^{1}**Re**	0.78	-0.72	0.40	-0.46	0.00
^{1}**TS**	0.77	-0.84	0.43	-0.36	0.00
^{1}**Pr**	0.75	-1.20	0.43	0.03	0.00

UB3LYP/BS2 + sol	Fe	ligand	H	TEMPO	Total
^{1}**Re**	1.41	-1.36	0.25	-0.30	0.00
^{1}**TS**	1.47	-1.87	0.30	0.11	0.00
^{1}**Pr**	1.20	-1.50	0.17	0.14	0.00

11. Cartesian Coordinates

[1] [(TPP)Fe^{III}($O_2^{-•}$)]:

26	21.639398000	0.411774000	1.540174000
7	23.047263000	0.149374000	0.150362000
7	21.327844000	-1.554945000	1.618689000
7	20.293708000	0.689511000	3.027689000
7	22.026583000	2.392730000	1.559416000
6	22.060475000	-2.547544000	0.970173000
6	21.551989000	-3.847789000	1.346804000
6	20.485781000	-3.642601000	2.174114000
6	20.343553000	-2.213464000	2.349215000
6	19.400497000	-1.623744000	3.201894000
6	19.412391000	-0.263188000	3.529920000
6	18.550125000	0.348072000	4.521172000
6	18.940046000	1.648436000	4.649451000
6	20.013377000	1.872241000	3.702359000
6	20.629456000	3.110584000	3.489549000
6	21.552566000	3.337303000	2.461958000
6	22.077263000	4.643390000	2.118595000
6	22.845711000	4.490669000	1.002191000
6	22.817947000	3.085753000	0.650220000
6	23.546415000	2.526001000	-0.406580000
6	23.653955000	1.144779000	-0.613235000
6	24.466244000	0.534944000	-1.643569000
6	24.380724000	-0.817790000	-1.481801000
6	23.489657000	-1.062761000	-0.370095000
6	23.090597000	-2.338524000	0.046912000
8	20.378606000	0.663085000	0.337793000
8	20.245113000	-0.142764000	-0.714673000
6	20.242439000	4.269260000	4.361194000
6	21.154741000	4.788084000	5.299530000
6	18.971456000	4.865277000	4.254109000
6	20.803588000	5.871602000	6.113112000
1	22.137761000	4.336949000	5.391721000
6	18.620601000	5.948766000	5.067839000
1	18.262534000	4.481306000	3.527416000
6	19.535191000	6.454481000	6.000272000
1	21.517823000	6.257075000	6.833799000
1	17.637671000	6.398565000	4.970052000
1	19.262918000	7.294715000	6.630858000
6	18.368443000	-2.511527000	3.831470000
6	18.385387000	-2.785093000	5.212703000
6	17.362356000	-3.098335000	3.039932000
6	17.418896000	-3.618535000	5.787429000
1	19.162030000	-2.349255000	5.833088000
6	16.395262000	-3.930434000	3.615261000
1	17.338076000	-2.894972000	1.973997000
6	16.420162000	-4.192464000	4.990814000
1	17.449224000	-3.821773000	6.853188000
1	15.624055000	-4.370295000	2.990962000
1	15.670589000	-4.838171000	5.436856000
6	23.759531000	-3.527052000	-0.574825000
6	25.123772000	-3.781209000	-0.333749000
6	23.048819000	-4.405100000	-1.416038000
6	25.757994000	-4.886305000	-0.912464000
1	25.683019000	-3.113299000	0.313798000
6	23.684031000	-5.509015000	-1.996068000
1	22.000381000	-4.214221000	-1.621188000
6	25.040052000	-5.753894000	-1.745107000
1	26.808724000	-5.069728000	-0.711459000
1	23.122006000	-6.172413000	-2.645683000
1	25.532158000	-6.610272000	-2.194757000
6	24.282084000	3.452793000	-1.327451000
6	25.689628000	3.491372000	-1.348136000
6	23.570865000	4.312101000	-2.187232000
6	26.367608000	4.362814000	-2.208166000
1	26.250260000	2.842379000	-0.682889000
6	24.249308000	5.182066000	-3.048239000
1	22.485669000	4.290350000	-2.182813000
6	25.649508000	5.209888000	-3.061609000
1	27.452795000	4.382384000	-2.208155000

1	23.685477000	5.833660000	-3.708184000
1	26.175229000	5.885041000	-3.729093000
1	25.023865000	1.074497000	-2.391459000
1	24.852631000	-1.583849000	-2.075198000
1	21.954650000	-4.790320000	1.014016000
1	19.862889000	-4.386510000	2.643612000
1	17.764919000	-0.160078000	5.056502000
1	18.532659000	2.396086000	5.310139000
1	21.868658000	5.553294000	2.657520000
1	23.390673000	5.250893000	0.466502000
8	23.109872000	0.195918000	3.017844000
6	23.058889000	-0.796142000	4.127443000
6	24.366062000	0.992274000	3.092382000
6	24.197702000	-0.382151000	5.063363000
1	22.066458000	-0.735596000	4.572261000
6	25.235466000	0.237171000	4.100869000
1	23.851805000	0.367810000	5.783816000
1	25.808929000	-0.551313000	3.600147000
1	24.780565000	1.039384000	2.086151000
1	24.105759000	1.997492000	3.434075000
1	23.212011000	-1.788489000	3.695499000
1	24.596025000	-1.233657000	5.621832000
1	25.939777000	0.900909000	4.609831000

TEMPO-H:

1	-2.719550000	-1.111552000	-1.691886000
7	-4.447252000	-1.843530000	-2.436336000
6	-3.871066000	-3.009947000	-3.198251000
6	-4.931272000	-0.596671000	-3.131127000
6	-4.885592000	-3.355408000	-4.319979000
6	-5.896612000	-1.058655000	-4.254062000
6	-5.298345000	-2.141230000	-5.164808000
6	-2.457555000	-2.767880000	-3.790319000
6	-3.794469000	-4.191886000	-2.208570000
6	-5.728393000	0.212134000	-2.085904000
6	-3.810055000	0.307460000	-3.707843000
1	-4.446788000	-4.141442000	-4.946933000
1	-5.783332000	-3.779134000	-3.850197000
1	-6.804551000	-1.457247000	-3.781771000
1	-6.194259000	-0.175830000	-4.833501000
1	-4.439331000	-1.746921000	-5.722706000
1	-6.039106000	-2.446035000	-5.914588000
1	-2.068906000	-3.711198000	-4.189665000
1	-1.751828000	-2.433091000	-3.021045000
1	-2.454463000	-2.037633000	-4.602224000
1	-3.485387000	-5.102398000	-2.734778000
1	-4.774577000	-4.363077000	-1.752509000
1	-3.075121000	-3.986742000	-1.411517000
1	-6.210171000	1.072184000	-2.565128000
1	-5.072393000	0.576080000	-1.290929000
1	-6.500474000	-0.418691000	-1.634226000
1	-4.248603000	1.253362000	-4.044523000
1	-3.295988000	-0.141476000	-4.560414000
1	-3.063322000	0.556650000	-2.944875000
8	-3.555013000	-1.482722000	-1.315661000

^{1} [(TPP)Fe^{III}(O$_2^{-\bullet}$)]+ TEMPO-H, ^{1}Re:

26	0.578887000	0.279766000	0.562019000
7	1.856868000	-0.021904000	-0.938794000
7	0.275154000	-1.685788000	0.699960000
7	-0.660560000	0.595055000	2.111644000
7	0.914616000	2.250656000	0.461804000
6	0.980588000	-2.696149000	0.053261000
6	0.462621000	-3.982898000	0.464460000
6	-0.591882000	-3.747426000	1.299842000
6	-0.705150000	-2.313794000	1.458291000
6	-1.605892000	-1.694026000	2.333812000
6	-1.534133000	-0.336273000	2.664970000
6	-2.307798000	0.292570000	3.713673000
6	-1.870625000	1.581237000	3.824473000
6	-0.867649000	1.785109000	2.802097000
6	-0.268568000	3.016834000	2.513910000

6	0.530059000	3.221324000	1.382655000	6	1.931179000	-4.637628000	-2.290854000
6	0.981241000	4.519271000	0.931994000	6	4.665056000	-5.038630000	-1.853245000
6	1.607665000	4.337909000	-0.267525000	1	4.587476000	-3.216378000	-0.701331000
6	1.576989000	2.921505000	-0.560900000	6	2.573323000	-5.751701000	-2.843042000
6	2.212058000	2.333036000	-1.662357000	1	0.873467000	-4.477089000	-2.472753000
6	2.359456000	0.947338000	-1.803488000	6	3.941491000	-5.956751000	-2.624509000
6	3.122931000	0.305980000	-2.851673000	1	5.725509000	-5.190308000	-1.678730000
6	3.117649000	-1.035428000	-2.596997000	1	2.006991000	-6.454101000	-3.446213000
6	2.314246000	-1.246415000	-1.413767000	1	4.438734000	-6.821179000	-3.052520000
6	1.974061000	-2.510417000	-0.915195000	6	2.806873000	3.235773000	-2.700866000
8	-0.911237000	0.485539000	-0.580988000	6	4.201212000	3.331347000	-2.872836000
8	-1.047013000	-0.396140000	-1.607768000	6	1.970282000	4.015514000	-3.522692000
6	-0.555473000	4.191426000	3.401656000	6	4.743662000	4.179664000	-3.844860000
6	0.458720000	4.724051000	4.220130000	1	4.858339000	2.745369000	-2.238175000
6	-1.829620000	4.789919000	3.428488000	6	2.513325000	4.862369000	-4.495544000
6	0.203633000	5.822432000	5.049297000	1	0.893773000	3.949476000	-3.400664000
1	1.445290000	4.271319000	4.208069000	6	3.901623000	4.946522000	-4.660017000
6	-2.083853000	5.888669000	4.257349000	1	5.820805000	4.244080000	-3.961617000
1	-2.617013000	4.397104000	2.793132000	1	1.853565000	5.451882000	-5.124128000
6	-1.068621000	6.407393000	5.070890000	1	4.322887000	5.603494000	-5.414073000
1	0.995668000	6.218011000	5.677170000	1	3.601886000	0.818489000	-3.669845000
1	-3.070771000	6.340222000	4.263634000	1	3.585210000	-1.817794000	-3.172367000
1	-1.266453000	7.259328000	5.713367000	1	0.846882000	-4.936836000	0.142736000
6	-2.665387000	-2.543884000	2.969441000	1	-1.220478000	-4.472571000	1.790781000
6	-2.613597000	-2.889047000	4.332757000	1	-3.067486000	-0.197900000	4.300113000
6	-3.735401000	-3.017598000	2.185478000	1	-2.210633000	2.334416000	4.516134000
6	-3.611042000	-3.689797000	4.901718000	1	0.818349000	5.445603000	1.458520000
1	-1.787832000	-2.536420000	4.942847000	1	2.064009000	5.087435000	-0.893549000
6	-4.731977000	-3.816888000	2.757743000	8	2.162203000	0.108525000	1.914467000
1	-3.785228000	-2.743744000	1.135441000	6	2.194942000	-0.833927000	3.070125000
6	-4.672732000	-4.155393000	4.115719000	6	3.403705000	0.932159000	1.877843000
1	-3.556281000	-3.951630000	5.953678000	6	3.394914000	-0.372964000	3.902121000
1	-5.554078000	-4.171741000	2.144154000	1	1.236365000	-0.754944000	3.581704000
1	-5.445963000	-4.775988000	4.557300000	6	4.352481000	0.221188000	2.844635000
6	2.647325000	-3.710193000	-1.509491000	1	3.745341000	0.956375000	0.843803000
6	4.023937000	-3.923560000	-1.301844000	1	-2.782574000	-1.104483000	-1.570457000

7	-4.478870000	-1.900567000	-2.381907000
6	-3.890106000	-3.048889000	-3.156898000
6	-4.987437000	-0.658813000	-3.063234000
6	-4.914120000	-3.407883000	-4.267165000
6	-5.967767000	-1.130050000	-4.169929000
6	-5.367121000	-2.197649000	-5.096766000
6	-2.487687000	-2.778585000	-3.762551000
6	-3.778785000	-4.240288000	-2.181129000
6	-5.776312000	0.140292000	-2.004047000
6	-3.890838000	0.262084000	-3.663842000
1	-4.467932000	-4.178980000	-4.907696000
1	-5.795413000	-3.855017000	-3.787369000
1	-6.860816000	-1.545546000	-3.683646000
1	-6.289343000	-0.249525000	-4.740447000
1	-4.525830000	-1.784285000	-5.667598000
1	-6.115557000	-2.512823000	-5.834983000
1	-2.086118000	-3.712189000	-4.173838000
1	-1.788319000	-2.429665000	-2.995830000
1	-2.507889000	-2.042522000	-4.569435000
1	-3.483867000	-5.144093000	-2.727362000
1	-4.744178000	-4.420281000	-1.697287000
1	-3.035184000	-4.043151000	-1.404590000
1	-6.289617000	0.984670000	-2.478622000
1	-5.109805000	0.527111000	-1.228939000
1	-6.522088000	-0.504114000	-1.527652000
1	-4.343111000	1.217133000	-3.955320000
1	-3.422230000	-0.166576000	-4.553004000
1	-3.103309000	0.477691000	-2.935002000
8	-3.613105000	-1.547860000	-1.243484000
1	3.146957000	1.941673000	2.209368000
1	4.897356000	-0.578695000	2.330542000
1	5.083334000	0.909382000	3.278100000
1	3.843952000	-1.199385000	4.459623000
1	3.093158000	0.399444000	4.618433000
1	2.322399000	-1.842881000	2.670557000

^{1} [(TPP)Fe^{III}(O$_2^{-\bullet}$)]+ TEMPO-H, ^{1}TS:

26	0.460641000	0.325617000	0.482823000
7	1.746406000	0.007065000	-1.015374000
7	0.119954000	-1.637061000	0.613595000
7	-0.757227000	0.652814000	2.053223000
7	0.885649000	2.290606000	0.433445000
6	0.803728000	-2.654621000	-0.045041000
6	0.300886000	-3.937709000	0.398068000
6	-0.722564000	-3.692843000	1.268168000
6	-0.834726000	-2.256706000	1.408588000
6	-1.720376000	-1.626711000	2.293288000
6	-1.644727000	-0.264978000	2.607475000
6	-2.424959000	0.381947000	3.641398000
6	-1.975608000	1.666480000	3.745422000
6	-0.954085000	1.848528000	2.736462000
6	-0.314732000	3.064937000	2.470316000
6	0.522053000	3.257220000	1.364784000
6	1.044491000	4.543902000	0.956426000
6	1.694396000	4.359293000	-0.229450000
6	1.599539000	2.952320000	-0.558833000
6	2.213103000	2.362415000	-1.672036000
6	2.284556000	0.975561000	-1.858121000
6	2.998476000	0.329596000	-2.939001000
6	2.928832000	-1.016809000	-2.724665000
6	2.138781000	-1.221973000	-1.530611000
6	1.771457000	-2.482500000	-1.041533000
8	-0.968375000	0.642866000	-0.658414000
8	-1.174980000	-0.252328000	-1.724399000
6	-0.588561000	4.238434000	3.364553000
6	0.414073000	4.721787000	4.226557000
6	-1.839674000	4.883980000	3.354855000
6	0.170312000	5.818423000	5.061540000
1	1.383090000	4.232653000	4.242854000
6	-2.083119000	5.980453000	4.190081000
1	-2.617700000	4.528451000	2.686734000
6	-1.079413000	6.450297000	5.046526000

1	0.953440000	6.175921000	5.722620000
1	-3.052403000	6.468353000	4.168009000
1	-1.268617000	7.300611000	5.693775000
6	-2.770750000	-2.469586000	2.951625000
6	-2.737214000	-2.749269000	4.330777000
6	-3.816640000	-3.005372000	2.174395000
6	-3.728040000	-3.543082000	4.920609000
1	-1.929510000	-2.351721000	4.937263000
6	-4.807111000	-3.797033000	2.766864000
1	-3.848034000	-2.786237000	1.111327000
6	-4.766207000	-4.068002000	4.140659000
1	-3.686082000	-3.753760000	5.984596000
1	-5.610167000	-4.199122000	2.157087000
1	-5.534455000	-4.682871000	4.598660000
6	2.398455000	-3.692342000	-1.665206000
6	3.778623000	-3.931338000	-1.515639000
6	1.634646000	-4.605760000	-2.417557000
6	4.376631000	-5.055953000	-2.095275000
1	4.378805000	-3.236011000	-0.937290000
6	2.233515000	-5.729184000	-2.998696000
1	0.573270000	-4.426623000	-2.554692000
6	3.605725000	-5.959008000	-2.837957000
1	5.440513000	-5.226865000	-1.964838000
1	1.630267000	-6.419832000	-3.579344000
1	4.069375000	-6.830775000	-3.288298000
6	2.870754000	3.261648000	-2.674964000
6	4.268756000	3.260780000	-2.846240000
6	2.096032000	4.136275000	-3.461629000
6	4.874551000	4.107784000	-3.781059000
1	4.879463000	2.600373000	-2.238930000
6	2.702215000	4.982051000	-4.397419000
1	1.017359000	4.145141000	-3.341174000
6	4.093211000	4.970222000	-4.560396000
1	5.953719000	4.096839000	-3.896667000
1	2.089139000	5.645709000	-4.998838000
1	4.563204000	5.626540000	-5.285720000
1	3.489255000	0.842197000	-3.750124000
1	3.347734000	-1.803546000	-3.330833000
1	0.676287000	-4.895279000	0.076415000
1	-1.331305000	-4.412725000	1.790812000
1	-3.197791000	-0.093624000	4.222854000
1	-2.315412000	2.429599000	4.426342000
1	0.912111000	5.464868000	1.500742000
1	2.203146000	5.099757000	-0.825103000
8	2.062243000	0.079460000	1.845358000
6	2.059164000	-0.868007000	2.995355000
6	3.324668000	0.868030000	1.826719000
6	3.273671000	-0.458401000	3.834796000
1	1.103486000	-0.757741000	3.506885000
6	4.251203000	0.117546000	2.785615000
1	3.670820000	0.899961000	0.794296000
1	-2.344129000	-1.014236000	-1.467682000
7	-4.006953000	-1.957852000	-2.300677000
6	-3.455993000	-3.062408000	-3.151836000
6	-4.841042000	-0.819908000	-2.809858000
6	-4.573488000	-3.515085000	-4.127433000
6	-5.891524000	-1.408435000	-3.787216000
6	-5.285132000	-2.353207000	-4.834114000
6	-2.181808000	-2.623915000	-3.921382000
6	-3.098359000	-4.242353000	-2.222806000
6	-5.567537000	-0.197010000	-1.598737000
6	-3.992292000	0.277835000	-3.508225000
1	-4.127684000	-4.204062000	-4.855033000
1	-5.317339000	-4.086340000	-3.555633000
1	-6.638695000	-1.961112000	-3.201361000
1	-6.411897000	-0.570770000	-4.267096000
1	-4.590749000	-1.812274000	-5.489563000
1	-6.078406000	-2.745668000	-5.482417000
1	-1.746083000	-3.494115000	-4.425565000
1	-1.434097000	-2.220678000	-3.231815000
1	-2.396809000	-1.867246000	-4.679727000
1	-2.800497000	-5.104925000	-2.829734000

1	-3.964859000	-4.525067000	-1.615957000
1	-2.275906000	-3.984491000	-1.552302000
1	-6.279464000	0.558313000	-1.949529000
1	-4.859359000	0.280518000	-0.917538000
1	-6.116170000	-0.966539000	-1.046133000
1	-4.620772000	1.157235000	-3.689505000
1	-3.597213000	-0.055730000	-4.471720000
1	-3.149568000	0.575972000	-2.878912000
8	-3.187259000	-1.627487000	-1.206449000
1	3.096787000	1.879773000	2.173128000
1	4.771521000	-0.693103000	2.262770000
1	5.002905000	0.777476000	3.227503000
1	3.694333000	-1.306114000	4.382521000
1	2.997277000	0.314670000	4.560700000
1	2.149866000	-1.879537000	2.591746000

^{1} [(TPP)$Fe^{III}(O_2^{-\bullet})$]+ TEMPO-H, ^{1}Pr:

26	21.756554000	-0.266587000	1.402219000
7	23.412285000	-0.360247000	0.249458000
7	21.857297000	-2.239873000	1.767730000
7	20.182148000	-0.139547000	2.631520000
7	21.736861000	1.729146000	1.125015000
6	22.855636000	-3.105740000	1.339654000
6	22.534331000	-4.456826000	1.757773000
6	21.322602000	-4.410239000	2.382278000
6	20.907246000	-3.022758000	2.411214000
6	19.746438000	-2.554623000	3.037547000
6	19.456241000	-1.190445000	3.175758000
6	18.413200000	-0.665183000	4.031029000
6	18.525338000	0.695328000	4.019036000
6	19.611835000	1.031189000	3.122127000
6	19.983263000	2.339726000	2.785211000
6	20.945533000	2.641445000	1.812006000
6	21.203693000	3.977195000	1.312721000
6	22.132411000	3.866392000	0.318843000
6	22.475428000	2.463690000	0.205995000
6	23.475528000	1.961547000	-0.637827000
6	23.918823000	0.634151000	-0.582226000
6	25.013047000	0.098343000	-1.365385000
6	25.186232000	-1.200175000	-0.983709000
6	24.177369000	-1.495024000	0.014088000
6	23.967543000	-2.770760000	0.555711000
8	20.713673000	-0.418545000	-0.096307000
8	19.752175000	-1.578898000	-0.086280000
6	19.295439000	3.484088000	3.470867000
6	20.012997000	4.302521000	4.364881000
6	17.936949000	3.771082000	3.238933000
6	19.388123000	5.373590000	5.013641000
1	21.061210000	4.092135000	4.552791000
6	17.312059000	4.842933000	3.887019000
1	17.373963000	3.158450000	2.542653000
6	18.034873000	5.646526000	4.777506000
1	19.956131000	5.990713000	5.702626000
1	16.264635000	5.051440000	3.693096000
1	17.549668000	6.476967000	5.280265000
6	18.771360000	-3.537128000	3.609877000
6	19.106608000	-4.371708000	4.693157000
6	17.476093000	-3.628820000	3.061644000
6	18.174954000	-5.280221000	5.208963000
1	20.094526000	-4.300194000	5.136842000
6	16.546280000	-4.538198000	3.577745000
1	17.206779000	-2.985558000	2.229248000
6	16.893034000	-5.367804000	4.651855000
1	18.448465000	-5.913067000	6.047369000
1	15.554698000	-4.599787000	3.140527000
1	16.171416000	-6.072726000	5.052168000
6	24.958491000	-3.845772000	0.216699000
6	26.260301000	-3.802536000	0.751526000
6	24.618724000	-4.906302000	-0.645114000
6	27.194678000	-4.796949000	0.440019000
1	26.535088000	-2.989340000	1.416128000

6	25.553531000	-5.900084000	-0.957514000
1	23.622709000	-4.944853000	-1.074589000
6	26.843697000	-5.849405000	-0.414701000
1	28.192346000	-4.750288000	0.865095000
1	25.275659000	-6.708520000	-1.626341000
1	27.568103000	-6.620370000	-0.656949000
6	24.126762000	2.903918000	-1.604169000
6	25.479387000	3.271190000	-1.465306000
6	23.387437000	3.448826000	-2.672051000
6	26.076752000	4.154779000	-2.371560000
1	26.058545000	2.868039000	-0.640643000
6	23.985342000	4.331465000	-3.578683000
1	22.344809000	3.172067000	-2.792107000
6	25.332064000	4.686820000	-3.431671000
1	27.119371000	4.429613000	-2.246796000
1	23.401437000	4.737450000	-4.398633000
1	25.795677000	5.371509000	-4.134570000
1	25.572052000	0.647170000	-2.105675000
1	25.907831000	-1.907358000	-1.359556000
1	23.146254000	-5.324543000	1.573140000
1	20.766388000	-5.233570000	2.799533000
1	17.712395000	-1.263839000	4.589884000
1	17.925767000	1.409399000	4.559686000
1	20.723708000	4.873816000	1.670209000
1	22.561093000	4.657346000	-0.274994000
8	22.986245000	0.044427000	3.034751000
6	22.924654000	-0.725074000	4.310741000
6	24.060777000	1.075149000	3.105034000
6	23.811075000	0.064749000	5.276939000
1	21.877883000	-0.782725000	4.605499000
6	24.870852000	0.696420000	4.346932000
1	24.616946000	1.026162000	2.170267000
1	18.869898000	-1.140268000	0.066422000
7	16.396031000	-0.476259000	-0.901204000
6	16.158647000	-1.597506000	-1.891967000
6	16.204204000	0.998911000	-1.184492000

6	14.959849000	-1.229448000	-2.798434000
6	15.016269000	1.191366000	-2.157539000
6	15.015399000	0.207209000	-3.334921000
6	17.448160000	-1.824392000	-2.720311000
6	15.830473000	-2.870803000	-1.088370000
6	15.889320000	1.692675000	0.155065000
6	17.523069000	1.563512000	-1.767633000
1	14.927945000	-1.954963000	-3.619996000
1	14.032021000	-1.355821000	-2.223509000
1	14.080551000	1.066106000	-1.595678000
1	15.039302000	2.229008000	-2.511346000
1	15.899155000	0.354413000	-3.968437000
1	14.143374000	0.397344000	-3.971952000
1	17.336443000	-2.739094000	-3.312788000
1	18.311696000	-1.938756000	-2.058699000
1	17.645865000	-0.998747000	-3.409438000
1	15.571972000	-3.674662000	-1.785742000
1	14.977558000	-2.700345000	-0.423376000
1	16.684418000	-3.190019000	-0.487901000
1	15.695817000	2.754803000	-0.028704000
1	16.726507000	1.598773000	0.849094000
1	15.000703000	1.252182000	0.618970000
1	17.458212000	2.655585000	-1.820916000
1	17.722374000	1.185712000	-2.774058000
1	18.365423000	1.294601000	-1.122885000
8	17.139236000	-0.766464000	0.156047000
1	23.577844000	2.050825000	3.201023000
1	25.642938000	-0.039124000	4.093881000
1	25.360567000	1.565736000	4.794402000
1	24.251918000	-0.578902000	6.043148000
1	23.230995000	0.847005000	5.779565000
1	23.309919000	-1.727581000	4.109957000

^{1} [(F$_{20}$TPP)Fe^{III}(O$_2$$^{-\bullet}$)]:

26	0.541625000	0.260026000	0.584288000
7	1.762135000	-0.019183000	-0.986495000
7	0.233714000	-1.722667000	0.679001000

7	-0.673313000	0.550137000	2.169906000	6	1.969770000	-3.646302000	-2.098523000
7	0.839538000	2.255051000	0.491282000	6	2.913732000	-4.554230000	-1.609022000
6	0.683536000	-2.690637000	-0.208317000	6	1.463269000	-3.895919000	-3.377127000
6	0.182768000	-3.990867000	0.185409000	6	3.335380000	-5.655862000	-2.345191000
6	-0.552643000	-3.810886000	1.318188000	6	1.871510000	-4.989540000	-4.134226000
6	-0.528617000	-2.394577000	1.623013000	6	2.811234000	-5.872993000	-3.614871000
6	-1.206883000	-1.812801000	2.695139000	6	3.346465000	3.217519000	-2.195784000
6	-1.278694000	-0.439342000	2.931454000	6	4.523634000	3.768096000	-1.680341000
6	-2.052456000	0.163835000	3.997700000	6	3.026704000	3.546459000	-3.516363000
6	-1.929362000	1.514718000	3.868785000	6	5.344364000	4.601790000	-2.432452000
6	-1.070256000	1.757480000	2.728844000	6	3.832234000	4.376251000	-4.289313000
6	-0.682239000	3.024027000	2.288316000	6	4.996235000	4.906347000	-3.743758000
6	0.209840000	3.243152000	1.237252000	1	3.969411000	0.870497000	-3.348267000
6	0.648015000	4.549794000	0.793810000	1	3.408989000	-1.743357000	-3.344459000
6	1.552023000	4.351541000	-0.205918000	1	0.366775000	-4.915634000	-0.337556000
6	1.669184000	2.920314000	-0.399457000	1	-1.071907000	-4.562941000	1.890553000
6	2.476954000	2.324207000	-1.370078000	1	-2.619869000	-0.376413000	4.738723000
6	2.503782000	0.954489000	-1.640032000	1	-2.369065000	2.276592000	4.492249000
6	3.289356000	0.347107000	-2.695589000	1	0.302663000	5.491353000	1.189586000
6	3.002695000	-0.984690000	-2.694996000	1	2.076395000	5.102849000	-0.774366000
6	2.048832000	-1.214916000	-1.630591000	8	2.132758000	0.079042000	1.850734000
6	1.535895000	-2.466917000	-1.290247000	6	3.280162000	-0.863189000	1.664129000
8	-0.970274000	0.508691000	-0.533843000	6	2.326481000	0.884906000	3.096644000
8	-1.132596000	-0.332410000	-1.573275000	6	4.019433000	-0.827572000	3.002006000
6	-1.215572000	4.214406000	3.016569000	1	2.865280000	-1.835352000	1.403168000
6	-0.417570000	4.943556000	3.903518000	6	3.775630000	0.613518000	3.502104000
6	-2.533275000	4.651666000	2.853631000	1	2.115438000	1.923951000	2.849556000
6	-0.896979000	6.051527000	4.593694000	1	1.608112000	0.520066000	3.834282000
6	-3.036720000	5.755347000	3.534828000	1	4.453539000	1.312365000	2.999701000
6	-2.213910000	6.458014000	4.408181000	1	3.918895000	0.713752000	4.581213000
6	-1.930097000	-2.721847000	3.638499000	1	5.081609000	-1.057310000	2.883331000
6	-1.327574000	-3.179128000	4.813412000	1	3.587192000	-1.553009000	3.700033000
6	-3.234767000	-3.156335000	3.389957000	1	3.885509000	-0.480155000	0.839105000
6	-1.982949000	-4.026180000	5.700954000	9	0.527893000	-3.039741000	-3.920427000
6	-3.910685000	-4.004437000	4.261354000	9	1.349792000	-5.204062000	-5.389057000
6	-3.280633000	-4.440580000	5.421804000	9	3.221350000	-6.956246000	-4.353457000

9	4.268739000	-6.525885000	-1.830064000
9	3.457017000	-4.361058000	-0.353326000
9	-3.884818000	-2.741933000	2.245530000
9	-5.194607000	-4.413257000	3.983324000
9	-3.938060000	-5.278269000	6.289680000
9	-1.357279000	-4.456047000	6.848670000
9	-0.039390000	-2.785308000	5.116603000
9	-3.374151000	3.977668000	1.991781000
9	-4.338900000	6.157527000	3.346917000
9	-2.699926000	7.549688000	5.085626000
9	-0.081320000	6.741825000	5.460549000
9	0.892715000	4.559858000	4.113971000
9	1.877273000	3.038514000	-4.086734000
9	3.482979000	4.678362000	-5.585263000
9	5.798773000	5.728379000	-4.496979000
9	6.496680000	5.122408000	-1.889653000
9	4.898628000	3.483020000	-0.382336000

[1] $[(F_{20}TPP)Fe^{III}(O_2^{-\bullet})]$+ TEMPO-H, [1]Re:

26	0.566481000	0.256942000	0.601911000
7	1.785277000	-0.018303000	-0.968947000
7	0.266097000	-1.726564000	0.695395000
7	-0.657155000	0.542694000	2.180476000
7	0.849074000	2.250697000	0.503363000
6	0.724629000	-2.694016000	-0.188731000
6	0.232754000	-3.996445000	0.206290000
6	-0.513103000	-3.818033000	1.332735000
6	-0.501785000	-2.401069000	1.633310000
6	-1.198193000	-1.821397000	2.694441000
6	-1.272896000	-0.448781000	2.931651000
6	-2.059655000	0.151841000	3.989301000
6	-1.934568000	1.502873000	3.865752000
6	-1.063462000	1.748688000	2.736191000
6	-0.679091000	3.016486000	2.297333000
6	0.214039000	3.237215000	1.248104000
6	0.647474000	4.544711000	0.804431000
6	1.553673000	4.349742000	-0.193919000
6	1.677329000	2.919457000	-0.386865000
6	2.488303000	2.327917000	-1.357028000
6	2.520066000	0.958463000	-1.626357000
6	3.300753000	0.354916000	-2.687224000
6	3.017125000	-0.977703000	-2.687725000
6	2.072915000	-1.212651000	-1.616979000
6	1.568931000	-2.466412000	-1.274916000
8	-0.954652000	0.485377000	-0.501198000
8	-1.103435000	-0.364909000	-1.546970000
6	-1.219602000	4.205732000	3.021987000
6	-0.429196000	4.935297000	3.915192000
6	-2.536267000	4.641757000	2.848145000
6	-0.915023000	6.042720000	4.601653000
6	-3.046042000	5.744888000	3.525479000
6	-2.230833000	6.447959000	4.405600000
6	-1.946602000	-2.732214000	3.615775000
6	-1.376991000	-3.196607000	4.803993000
6	-3.244773000	-3.162775000	3.328922000
6	-2.057809000	-4.047574000	5.668345000
6	-3.945899000	-4.014234000	4.176599000
6	-3.348061000	-4.458003000	5.351356000
6	1.992967000	-3.643547000	-2.092680000
6	3.012955000	-4.497000000	-1.662813000
6	1.393400000	-3.947168000	-3.317892000
6	3.420450000	-5.599118000	-2.406577000
6	1.783504000	-5.043553000	-4.080416000
6	2.801659000	-5.872150000	-3.621739000
6	3.353102000	3.225054000	-2.183205000
6	4.528841000	3.779416000	-1.668774000
6	3.029518000	3.554839000	-3.502660000
6	5.344913000	4.617713000	-2.420871000
6	3.830431000	4.389121000	-4.275644000
6	4.993335000	4.922940000	-3.731155000
1	3.974617000	0.881769000	-3.343498000
1	3.417431000	-1.734779000	-3.342847000

1	0.430654000	-4.921360000	-0.311318000
1	-1.030720000	-4.571878000	1.904047000
1	-2.636733000	-0.390356000	4.721247000
1	-2.382271000	2.263174000	4.485326000
1	0.297246000	5.484919000	1.199039000
1	2.075314000	5.102900000	-0.762325000
8	2.137713000	0.090389000	1.856265000
6	3.291878000	-0.846623000	1.671217000
6	2.325035000	0.899281000	3.103113000
6	4.031827000	-0.799828000	3.007951000
1	2.881715000	-1.821891000	1.415553000
6	3.776928000	0.640468000	3.504681000
1	2.104056000	1.935686000	2.855275000
1	-2.965420000	-0.624321000	-1.728691000
7	-4.645813000	-1.369790000	-2.613132000
6	-4.292707000	-2.832420000	-2.670301000
6	-4.636974000	-0.496473000	-3.838174000
6	-5.149769000	-3.455026000	-3.804592000
6	-5.481230000	-1.228154000	-4.914997000
6	-5.056114000	-2.687726000	-5.131561000
6	-2.788122000	-3.137078000	-2.887316000
6	-4.729651000	-3.448972000	-1.323627000
6	-5.348093000	0.819313000	-3.455238000
6	-3.227272000	-0.153822000	-4.389266000
1	-4.842542000	-4.500798000	-3.931676000
1	-6.198158000	-3.462614000	-3.476117000
1	-6.532493000	-1.210953000	-4.596661000
1	-5.415664000	-0.656334000	-5.849183000
1	-4.037197000	-2.737948000	-5.536943000
1	-5.708240000	-3.157574000	-5.879040000
1	-2.613796000	-4.210651000	-2.748952000
1	-2.167254000	-2.608827000	-2.157217000
1	-2.436628000	-2.874066000	-3.887480000
1	-4.618619000	-4.539372000	-1.358152000
1	-5.779583000	-3.209013000	-1.126247000
1	-4.128118000	-3.061857000	-0.497741000

1	-5.480055000	1.446002000	-4.345058000
1	-4.766532000	1.376773000	-2.716572000
1	-6.331696000	0.602444000	-3.026459000
1	-3.321288000	0.598963000	-5.180606000
1	-2.719931000	-1.022278000	-4.815674000
1	-2.585957000	0.267810000	-3.608394000
8	-3.931826000	-0.711026000	-1.505237000
1	1.610969000	0.526636000	3.840596000
1	4.447576000	1.343885000	2.999054000
1	3.921513000	0.744202000	4.583231000
1	5.095750000	-1.020256000	2.887779000
1	3.607445000	-1.527098000	3.708744000
1	3.891813000	-0.461790000	0.843498000
9	0.378372000	-3.145149000	-3.799809000
9	1.168948000	-5.313338000	-5.281204000
9	3.195031000	-6.957338000	-4.366392000
9	4.430006000	-6.415571000	-1.951398000
9	3.646157000	-4.248811000	-0.460376000
9	-3.863782000	-2.737678000	2.170227000
9	-5.222428000	-4.418736000	3.862243000
9	-4.030071000	-5.299336000	6.196270000
9	-1.464171000	-4.485301000	6.829893000
9	-0.096495000	-2.807244000	5.143445000
9	-3.368932000	3.967027000	1.979247000
9	-4.346863000	6.145978000	3.327208000
9	-2.723194000	7.538962000	5.079350000
9	-0.106955000	6.733725000	5.474918000
9	0.879850000	4.552488000	4.135528000
9	1.881129000	3.042934000	-4.071740000
9	3.477952000	4.691520000	-5.570568000
9	5.791487000	5.748915000	-4.484538000
9	6.495706000	5.142221000	-1.878891000
9	4.906776000	3.493759000	-0.371682000

^{1} [(F_{20}TPP)Fe^{III}($O_2^{-\bullet}$)]+ TEMPO-H, ^{1}TS:

26	0.505622000	0.276658000	0.428979000
7	1.852128000	0.009821000	-1.033796000
7	0.191139000	-1.703884000	0.482243000
7	-0.806852000	0.548824000	1.932925000
7	0.845849000	2.267294000	0.416673000
6	0.685586000	-2.659023000	-0.394478000
6	0.195869000	-3.969944000	-0.020363000
6	-0.565593000	-3.809180000	1.098231000
6	-0.583089000	-2.392483000	1.404089000
6	-1.312346000	-1.822579000	2.449860000
6	-1.439171000	-0.447658000	2.660861000
6	-2.314734000	0.147355000	3.650902000
6	-2.227647000	1.499297000	3.503732000
6	-1.280403000	1.751839000	2.437749000
6	-0.870007000	3.022921000	2.031268000
6	0.137551000	3.251133000	1.092013000
6	0.636610000	4.559331000	0.720865000
6	1.657784000	4.363313000	-0.159646000
6	1.780026000	2.932619000	-0.360963000
6	2.671465000	2.339576000	-1.258488000
6	2.678503000	0.980564000	-1.580734000
6	3.527735000	0.385678000	-2.593902000
6	3.191860000	-0.932235000	-2.677564000
6	2.149544000	-1.169094000	-1.700257000
6	1.591394000	-2.419416000	-1.429185000
8	-0.866958000	0.610954000	-0.780581000
8	-1.031323000	-0.271063000	-1.855512000
6	-1.510123000	4.206436000	2.680770000
6	-0.845498000	4.960674000	3.652461000
6	-2.807253000	4.611329000	2.351932000
6	-1.431833000	6.062411000	4.266028000
6	-3.415897000	5.708370000	2.952925000
6	-2.723782000	6.437231000	3.913678000
6	-2.021588000	-2.737346000	3.396734000
6	-1.516700000	-2.973537000	4.679449000
6	-3.211105000	-3.390871000	3.062614000
6	-2.155188000	-3.812041000	5.586720000
6	-3.867201000	-4.236177000	3.951950000
6	-3.336949000	-4.446403000	5.219865000
6	2.053811000	-3.583763000	-2.243645000
6	2.953576000	-4.522658000	-1.729316000
6	1.619930000	-3.787047000	-3.556592000
6	3.396721000	-5.611266000	-2.472365000
6	2.050044000	-4.866808000	-4.321242000
6	2.942239000	-5.782911000	-3.775418000
6	3.652816000	3.231254000	-1.949358000
6	4.780588000	3.722030000	-1.284530000
6	3.490009000	3.625013000	-3.280955000
6	5.701080000	4.560886000	-1.903773000
6	4.397763000	4.461153000	-3.922875000
6	5.507644000	4.931785000	-3.229847000
1	4.279394000	0.907203000	-3.164443000
1	3.623206000	-1.678963000	-3.324915000
1	0.411078000	-4.889742000	-0.540040000
1	-1.074915000	-4.575938000	1.659573000
1	-2.928164000	-0.398524000	4.349790000
1	-2.743773000	2.256088000	4.072497000
1	0.253813000	5.499998000	1.082999000
1	2.255969000	5.115857000	-0.648114000
8	1.995102000	0.049414000	1.807137000
6	3.139924000	-0.905135000	1.685954000
6	2.110302000	0.831969000	3.077387000
6	3.769299000	-0.916870000	3.079139000
1	2.734055000	-1.862685000	1.364556000
6	3.516506000	0.518605000	3.590886000
1	1.944766000	1.879424000	2.830789000
1	-2.358339000	-0.863792000	-1.637894000
7	-4.171767000	-1.648410000	-2.275986000
6	-3.878966000	-3.000262000	-2.857160000
6	-4.742334000	-0.493982000	-3.045190000
6	-5.082078000	-3.403330000	-3.747693000
6	-5.908358000	-1.039661000	-3.910190000

6	-5.534520000	-2.295869000	-4.709820000
6	-2.552814000	-3.013734000	-3.659697000
6	-3.767864000	-3.998671000	-1.684358000
6	-5.299386000	0.516965000	-2.020155000
6	-3.683896000	0.217599000	-3.931590000
1	-4.807738000	-4.312876000	-4.295809000
1	-5.923462000	-3.663234000	-3.090954000
1	-6.749266000	-1.280351000	-3.245650000
1	-6.241536000	-0.233842000	-4.575261000
1	-4.747972000	-2.070347000	-5.441332000
1	-6.402278000	-2.639398000	-5.286573000
1	-2.292766000	-4.046816000	-3.916706000
1	-1.733556000	-2.601225000	-3.064246000
1	-2.623212000	-2.444506000	-4.590235000
1	-3.687678000	-5.017135000	-2.081278000
1	-4.655925000	-3.938611000	-1.047220000
1	-2.890087000	-3.791881000	-1.069192000
1	-5.825760000	1.319866000	-2.547902000
1	-4.495806000	0.957844000	-1.425485000
1	-6.002312000	0.022299000	-1.342218000
1	-4.116153000	1.140103000	-4.335764000
1	-3.366982000	-0.401252000	-4.775067000
1	-2.798163000	0.480856000	-3.346615000
8	-3.259462000	-1.269705000	-1.269177000
1	1.327603000	0.474913000	3.750439000
1	4.247387000	1.211605000	3.159618000
1	3.575023000	0.593239000	4.679979000
1	4.832408000	-1.168841000	3.041009000
1	3.265792000	-1.646867000	3.722579000
1	3.816342000	-0.510401000	0.923972000
9	0.734418000	-2.895842000	-4.129397000
9	1.597880000	-5.034230000	-5.609847000
9	3.374168000	-6.853070000	-4.520674000
9	4.283038000	-6.513697000	-1.930518000
9	3.428118000	-4.376223000	-0.439954000
9	-3.768707000	-3.201709000	1.814739000
9	-5.037783000	-4.860359000	3.586848000
9	-3.977378000	-5.277809000	6.106472000
9	-1.624681000	-4.020195000	6.839361000
9	-0.343324000	-2.360452000	5.073165000
9	-3.520811000	3.910241000	1.401383000
9	-4.693405000	6.078050000	2.600565000
9	-3.313534000	7.524031000	4.512435000
9	-0.745582000	6.778801000	5.219681000
9	0.436589000	4.610432000	4.028535000
9	2.396897000	3.179121000	-3.995889000
9	4.202575000	4.828417000	-5.234491000
9	6.410127000	5.759043000	-3.853138000
9	6.800094000	5.021408000	-1.215553000
9	5.003412000	3.370615000	0.032276000

^{1} [(TPP)Fe^{III}(O$_2^{-\bullet}$)]+ TEMPO-H, ^{1}Pr:

26	21.696991000	-0.319092000	1.535296000
7	23.274929000	-0.397448000	0.268199000
7	21.802993000	-2.304786000	1.832389000
7	20.195630000	-0.216979000	2.864327000
7	21.659791000	1.687267000	1.296549000
6	22.731973000	-3.172677000	1.280647000
6	22.436612000	-4.533402000	1.688926000
6	21.314338000	-4.485969000	2.459075000
6	20.923406000	-3.093640000	2.556602000
6	19.848133000	-2.625809000	3.312548000
6	19.534943000	-1.274342000	3.469031000
6	18.506383000	-0.769625000	4.356522000
6	18.554000000	0.590359000	4.291985000
6	19.596507000	0.937315000	3.345121000
6	19.906176000	2.243487000	2.958117000
6	20.844190000	2.577061000	1.977708000
6	21.078243000	3.925635000	1.497643000
6	22.023875000	3.845091000	0.520221000
6	22.391505000	2.448528000	0.397899000

6	23.377128000	1.963894000	-0.464639000	1	17.852787000	-1.375354000	4.963728000
6	23.789793000	0.630446000	-0.507738000	1	17.942283000	1.293108000	4.834735000
6	24.851712000	0.124835000	-1.355119000	1	20.574373000	4.811089000	1.850852000
6	24.985280000	-1.202545000	-1.079817000	1	22.443621000	4.654796000	-0.055191000
6	23.992381000	-1.532105000	-0.074544000	8	23.028541000	-0.050105000	3.083611000
6	23.770621000	-2.821456000	0.415349000	6	23.102244000	-0.906002000	4.305618000
8	20.579355000	-0.446161000	0.111529000	6	24.070188000	1.019896000	3.143565000
8	19.513313000	-1.499905000	0.248517000	6	24.007356000	-0.127552000	5.261167000
6	19.166822000	3.364122000	3.616464000	1	22.084694000	-1.049677000	4.665292000
6	19.780584000	4.175741000	4.575720000	6	24.974277000	0.607518000	4.306937000
6	17.836969000	3.661546000	3.305986000	1	24.569019000	1.047623000	2.176019000
6	19.113479000	5.226879000	5.195917000	1	18.684049000	-0.952314000	0.118027000
6	17.147272000	4.705719000	3.913234000	7	16.515605000	-0.025326000	-1.269120000
6	17.789947000	5.492729000	4.862367000	6	16.292963000	-1.376990000	-1.919188000
6	19.003004000	-3.625989000	4.032794000	6	16.280777000	1.325513000	-1.916510000
6	19.450774000	-4.269450000	5.190454000	6	15.122248000	-1.274639000	-2.926260000
6	17.721498000	-3.963032000	3.586295000	6	15.131024000	1.223681000	-2.947115000
6	18.674720000	-5.202090000	5.870084000	6	15.194994000	-0.033068000	-3.823598000
6	16.925013000	-4.890587000	4.250101000	6	17.598960000	-1.823343000	-2.621216000
6	17.404768000	-5.513239000	5.396991000	6	15.928244000	-2.377895000	-0.804153000
6	24.686365000	-3.906580000	-0.054903000	6	15.887722000	2.318362000	-0.804962000
6	25.968331000	-4.060022000	0.480618000	6	17.603689000	1.778930000	-2.580085000
6	24.311913000	-4.811025000	-1.052771000	1	15.117383000	-2.194401000	-3.522792000
6	26.837831000	-5.057174000	0.050973000	1	14.176255000	-1.250103000	-2.367972000
6	25.162170000	-5.818237000	-1.497633000	1	14.174149000	1.224008000	-2.407335000
6	26.431540000	-5.940242000	-0.943237000	1	15.151998000	2.133718000	-3.558284000
6	24.063791000	2.941054000	-1.362256000	1	16.108379000	-0.042189000	-4.431726000
6	25.361228000	3.390836000	-1.099121000	1	14.353894000	-0.034981000	-4.526980000
6	23.442194000	3.454778000	-2.504080000	1	17.466192000	-2.838730000	-3.010584000
6	26.013342000	4.299953000	-1.925228000	1	18.430689000	-1.831143000	-1.911850000
6	24.072191000	4.367247000	-3.344208000	1	17.857844000	-1.170409000	-3.459472000
6	25.364074000	4.790807000	-3.052795000	1	15.696680000	-3.347339000	-1.257993000
1	25.413131000	0.706664000	-2.068520000	1	15.046914000	-2.035096000	-0.252257000
1	25.674551000	-1.899158000	-1.530017000	1	16.749369000	-2.514062000	-0.097621000
1	23.007811000	-5.406170000	1.415512000	1	15.657875000	3.288253000	-1.259307000
1	20.800073000	-5.314090000	2.919883000	1	16.694000000	2.449645000	-0.082429000

1	14.997146000	1.966812000	-0.273449000
1	17.501236000	2.812276000	-2.928499000
1	17.868676000	1.155418000	-3.438858000
1	18.418692000	1.734032000	-1.851701000
8	17.275537000	-0.002097000	-0.184172000
1	23.556470000	1.966440000	3.327503000
1	25.760355000	-0.073113000	3.961088000
1	25.452109000	1.472377000	4.774949000
1	24.526187000	-0.790530000	5.958710000
1	23.423372000	0.594068000	5.843387000
1	23.532628000	-1.865847000	4.010027000
9	22.161018000	3.052529000	-2.823265000
9	23.428344000	4.849064000	-4.460923000
9	25.996919000	5.691252000	-3.875351000
9	27.290613000	4.720268000	-1.631950000
9	26.030011000	2.927173000	0.017315000
9	23.060607000	-4.714177000	-1.627118000
9	24.757776000	-6.689506000	-2.483144000
9	27.280947000	-6.929780000	-1.376098000
9	28.092260000	-5.176978000	0.604251000
9	26.401252000	-3.200757000	1.471300000
9	17.210360000	-3.363921000	2.452083000
9	15.669378000	-5.197245000	3.777965000
9	16.627343000	-6.431584000	6.060549000
9	19.152274000	-5.813080000	7.007052000
9	20.705060000	-3.977754000	5.690259000
9	17.168091000	2.899971000	2.367640000
9	15.838043000	4.965653000	3.579026000
9	17.120038000	6.528052000	5.468344000
9	19.752262000	6.001011000	6.137455000
9	21.093639000	3.938251000	4.93202900

12. References

[1] M. K. Peters, F. Röhricht, C. Näther and R. Herges, *Org. Lett.*, 2018, **20**, 7879–7883.

[2] A. Z. Muresan, P. Thamyongkit, J. R. Diers, D. Holten, J. S. Lindsey and D. F. Bocian, *J. Org. Chem.*, 2008, **73**, 6947–6959.

[3] M. Y. Hyun, Y. D. Jo, J. H. Lee, H. G. Lee, H. M. Park, I. H. Hwang, K. B. Kim, S. J. Lee and C. Kim, *Chem. Eur. J.*, 2013, **19**, 1810–1818.

[4] (a) E. A. Mader, E. R. Davidson and J. M. Mayer, *J. Am. Chem. Soc.*, 2007, **129**, 5153–5166; (b) A. Wu, E. A. Mader, A. Datta, D. A. Hrovat, W. T. Borden and J. M. Mayer, *J. Am. Chem. Soc.*, 2009, **131**, 11985–11997.

[5] J. J. Curley, R. G. Bergman and T. D. Tilley, *Dalton Trans.*, 2012, **41**, 192–200.

[6] R. A. Ghiladi, R. M. Kretzer, I. Guzei, A. L. Rheingold, Y.-M. Neuhold, K. R. Hatwell, A. D. Zuberbuhler and K. D. Karlin, *Inorg. Chem.*, 2001, **40**, 5754–5767.

[7] M. J. Frisch, G. W. T., H. B. Schlegel, G. E. Scuseria, M. A. Robb, J. R. Cheeseman, G. Scalmani, V. Barone, B. Mennucci, G. A. Petersson, H. Nakatsuji, M. Caricato, X. Li, H. P. Hratchian, A. F. Izmaylov, J. Bloino, G. Zheng, J. L. Sonnenberg, M. Hada, M. Ehara, K. Toyota, R. Fukuda, J. Hasegawa, M. Ishida, T. Nakajima, Y. Honda, O. Kitao, H. Nakai, T. Vreven, J. A. Montgomery, Jr., J. E. Peralta, F. Ogliaro, M. Bearpark, J. J. Heyd, E. Brothers, K. N. Kudin, V. N. Staroverov, R. Kobayashi, J. Normand, K. Raghavachari, A. Rendell, J. C. Burant, S. S. Iyengar, J. Tomasi, M. Cossi, N. Rega, J. M. Millam, M. Klene, J. E. Knox, J. B. Cross, V. Bakken, C. Adamo, J. Jaramillo, R. Gomperts, R. E. Stratmann, O. Yazyev, A. J. Austin, R. Cammi, C. Pomelli, J. W. Ochterski, R. L. Martin, K. Morokuma, V. G. Zakrzewski, G. A. Voth, P. Salvador, J. J. Dannenberg, S. Dapprich, A. D. Daniels, Ö. Farkas, J. B. Foresman, J. V. Ortiz, J. Cioslowski, and D. J. Fox, Gaussian 09 (Gaussian, Inc., Wallingford CT, 2009).

[8] (a) A. D. Becke, *Phys. Rev. A.*, 1988, **38**, 3098; (b) C. Lee, W. Yang and R. G. Parr, *Phys. Rev. B.*, 1988, **37**, 785.

[9] W. R. Wadt and P. J. Hay, *J. Chem. Phys.*, 1985, **82**, 284.

[10] M. Cossi, G. Scalmani, N. Rega and V. Barone, *J. Chem. Phys.*, 2002, **117**, 43.

[11] M. Bhadra, J. Y. C. Lee, R. E. Cowley, S. Kim, M. A. Siegler, E. I. Solomon and K. D. Karlin, *J. Am. Chem. Soc.*, 2018, **140**, 9042−9045.

[12] (a) K. Tajima, S. Oka, T. Edo, S. Miyake, H. Mano, K. Mukai, H. Sakurai and K. lshizua, *J. Chem. Soc., Chem. Commun.*, 1995, 1507; (b) K. Tajima, M. Shigematsu, J. Jinno, K. Ishizu and H. Ohya-Nishiguchic, *J. Chem. Soc., Chem. Commun.*, 1990, 144; (c) J.-G. Liu, T. Ohta, S. Yamaguchi, T. Ogura, S. Sakamoto, Y. Maeda and Y. Naruta, *Angew. Chem., Int. Ed.*, 2009, **48**, 9262–9267; (d) P. Nagaraju, T. Ohta, J.-G. Liu, T. Ogura and Y. Naruta, *Chem. Commun.*, 2016, **52**, 7213–7216; (e) R. Oliveira, W. Zouari, C. Herrero, F. Banse, B. Schöllhorn, C. Fave and E. Anxolabéhère-Mallart, *Inorg. Chem.*, 2016, **55**, 12204–12210; (f) J.-G. Liu, Y. Shimizu, T. Ohta and Y. Naruta, *J. Am. Chem. Soc.*, 2010, **132**, 3672–3673; (g) H. Kim, P. J. Rogler, S. K. Sharma, A. W. Schaefer, E. I. Solomon and K. D. Karlin, *J. Am. Chem. Soc.*, 2020, **142**, 3104–3116; (h) Y. Goto, S. Wada, I. Morishima and Y. Watanabe, *J. Inorg. Biochem.*, 1998, **69**, 241–247; (i) M. Selke, M. F. Sisemore and J. S. Valentine, *J. Am. Chem. Soc.*, 1996, **118**, 2008–2012; (j) M. Selke and J. S. Valentine, *J. Am. Chem. Soc.*, 1998, **120**, 2652–2653; (k) E. McCandlish, A. R. Mikszta, M. Nappa, A. Q. Sprenger, J. S. Valentine, J. D. Stong and T. G. Spiro, *J. Am. Chem. Soc.*, 1980, **102**, 4268–4271.
